# TSHZ3 and SOX9 Regulate the Timing of Smooth Muscle Cell Differentiation in the Ureter by Reducing Myocardin Activity

**DOI:** 10.1371/journal.pone.0063721

**Published:** 2013-05-06

**Authors:** Elise Martin, Xavier Caubit, Rannar Airik, Christine Vola, Ahmed Fatmi, Andreas Kispert, Laurent Fasano

**Affiliations:** 1 Aix-Marseille Université, CNRS, IBDM UMR7288, Marseille, France; 2 Institut für Molekularbiologie, Medizinische Hochschule Hannover, Hannover, Germany; William Harvey Research Institute, Barts and The London School of Medicine and Dentistry, Queen Mary University of London, United Kingdom

## Abstract

Smooth muscle cells are of key importance for the proper functioning of different visceral organs including those of the urogenital system. In the mouse ureter, the two transcriptional regulators TSHZ3 and SOX9 are independently required for initiation of smooth muscle differentiation from uncommitted mesenchymal precursor cells. However, it has remained unclear whether TSHZ3 and SOX9 act independently or as part of a larger regulatory network. Here, we set out to characterize the molecular function of TSHZ3 in the differentiation of the ureteric mesenchyme. Using a yeast-two-hybrid screen, we identified SOX9 as an interacting protein. We show that TSHZ3 also binds to the master regulator of the smooth muscle program, MYOCD, and displaces it from the coregulator SRF, thereby disrupting the activation of smooth muscle specific genes. We found that the initiation of the expression of smooth muscle specific genes in MYOCD-positive ureteric mesenchyme coincides with the down regulation of *Sox9* expression, identifying SOX9 as a possible negative regulator of smooth muscle cell differentiation. To test this hypothesis, we prolonged the expression of *Sox9* in the ureteric mesenchyme *in vivo*. We found that *Sox9* does not affect *Myocd* expression but significantly reduces the expression of MYOCD/SRF-dependent smooth muscle genes, suggesting that down-regulation of *Sox9* is a prerequisite for MYOCD activity. We propose that the dynamic expression of *Sox9* and the interaction between TSHZ3, SOX9 and MYOCD provide a mechanism that regulates the pace of progression of the myogenic program in the ureter.

## Introduction

Smooth muscle (SM) is a frequent investment of visceral organs, and is required to assure their rigidity and contractility. In the urinary tract, SM is present in the renal pelvis, the ureter, the bladder and the urethra and plays a crucial role in the functional and structural integrity of these organs.

*In vivo* characterization of visceral smooth muscle cell (SMC) development in the ureter has provided evidence for a role for Sonic hedgehog (SHH) and Bone morphogenetic protein 4 (BMP4) signaling in the differentiation of the mesenchymal cells that surround the urothelium [1], [2], [3], [4], [5], [6], [7], [8]. Targeted removal of *Shh* signal production in the ureteric epithelium resulted in a reduction of ureteral mesenchymal cell proliferation and delayed SM differentiation, which is likely to play a causal role in the observed hydroureter [8]. Moreover, removal of *Shh* resulted in a deficit of *Bmp4*-expressing peri-ureteral mesenchyme, while conditional deletion of *Bmp4* dramatically reduced expression of SM specific genes [1], [8]. Four transcription factors, the T-box protein TBX18, the zinc-finger protein Teashirt 3 (TSHZ3), the homeodomain-containing protein SIX1 and the high-mobility-group (HMG) domain transcription factor SOX9 are expressed in SMC precursors and are important for the cytodifferentiation of ureteric mesenchyme [9], [10], [11], [12]. Phenotypic and molecular analyses of *Tbx18* mutant ureters have indicated that *Tbx18* is required for specifying mesenchymal cell precursors to a ureteric fate [9]. *Six1* acts synergistically with *Tbx18* in regulating the proliferation and differentiation of SMC precursors [12]. Teashirt 3 (*Tshz3*) is expressed in precursors, and in differentiating and differentiated SMCs; its expression is maintained in adult ureteric SM. In *Tshz3* mutant ureters the myogenic program is not activated in the proximal region due to the absence of expression of *myocardin* (*Myocd*), a key regulator of SM differentiation [11], [13]. It was suggested that SM differentiation depends on a signaling axis starting with secreted SHH and ending with ureteric SMC differentiation, with *Tszh3* downstream of *Bmp4* but upstream of *Myocd* and SMC contractile protein synthesis [11], [14]. This work also provided some indication concerning the temporal control of SMC differentiation. In the wild type ureter, MYOCD and SMC markers are detected at E15.5, shortly before onset of urine production at E16.0 to E16.5. *Tshz3^−/−^* mice as well as other mutants with defects in ureteric SM differentiation consequently develop hydroureter at around E16.5 [11]. Recently, it was shown that the high mobility group domain transcription factor gene *Sox9* plays an important role both in kidney and ureter development in the mouse [10], [15]. *Sox9* is transiently expressed in the undifferentiated ureteric mesenchyme. Conditional inactivation of *Sox9* in this domain led to a significant down-regulation of *Myocd* expression and perturbed differentiation into SMC, which resulted in strong proximal hydroureter formation due to functional obstruction. The involvement of visceral SMCs in pelvi-ureteric junction obstruction (PUJO), a frequent cause for persisting dilatation of the upper urinary tract (hydronephrosis), underlies the significant interest in understanding the mechanisms that control the differentiation of visceral SMCs [16], [17], [18], [19].

Previous data suggested that both TSHZ3 and SOX9 critically regulate the onset of the myogenic program in the developing ureter. However it is not clear whether TSHZ3 and SOX9 act independently or are part of a regulatory network that controls cytodifferentiation of the ureteric mesenchyme. Here, we set out to characterize the molecular function of TSHZ3 in the development of the ureteric mesenchyme. Using a yeast-two-hybrid screen, we identified SOX9 as an interacting protein. We verify this interaction *in vitro* and *in vivo*, and define its temporal nature in the ureter. We show that TSHZ3 also binds to MYOCD, and that the ternary complex between SOX9, TSHZ3 and MYOCD disrupts the activation of SM specific genes. Therefore, despite controlling the onset of the myogenic program by inducing the expression of *Myocd*, TSHZ3 and SOX9 subsequently delay the differentiation of visceral SMCs by inhibiting Myocardin activity. We therefore propose that the dynamic expression of *Sox9* and the interaction between TSHZ3, SOX9 and MYOCD provide a mechanism that regulates the pace of progression of the myogenic program in the ureter.

## Materials and Methods

### Ethics Statement

All experiments were performed in accordance with the European legislation (86/609/CEE), the guidelines established by the French Ministry of Agriculture (Animal Rights Division) and approved by H. Hedrich, state head of the animal facility at Medizinische Hochschule Hannover and performed according to German legislation. The architecture and functioning rules of our animal house, as well as our experimental procedures have been approved by the “Direction Départementale des Services Vétérinaires” (ID number E-13-055-21). Experiments were not performed on living animals.

### Mouse strains

Wild–type and *Tshz3^lacZ^* mouse lines used in this study were maintained on a CD1 outbred background. The *Tshz3^lacZ^* allele carries an in-frame insertion of *lacZ* coding sequence within the second exon of *Tshz3* [11]. For conditional prolonged *Sox9* expression, females homozygous for an *Hprt^Sox9^*-allele were crossed with *Tbx18^Cre/+^* males as previously described [10].

### Cell lines

The murine C3H10T1/2 cell line was purchased from American Type Culture Collection (ATCC number CCL-226); the human HEK293T cell line was a gift from Dr. J. Iovanna (CRCM, Marseille, France). Cell lines were cultivated following the ATCC culture protocol and maintained in Dulbecco's Modified Eagle's Medium with supplement of 10% fetal bovine serum and 50 units/ml penicillin, 50 mg/ml streptomycin, 1% sodium pyruvate.

### Yeast Two-hybrid Screen (Y2H)

The screen has been previously reported in [20].

### Immunological and *in situ* hybridization analysis

Tissues were fixed in 4% paraformaldehyde/PBS. Immunostaining was performed either on 10 μm cryosections of tissues or on paraffin embedded sections after quenching endogenous peroxidase activity and antigen retrieval followed by reaction with secondary antibodies. Primary and secondary antibodies used were mouse anti-smooth muscle α actin (SMaA, 1A4, Sigma; 1/1000), guinea-pig anti-TSHZ3 antibody (1/5000) produced by A. Garratt's laboratory (Max-Delbrueck-Center, Berlin, Germany). Rabbit anti-SOX9 antibody (1/1000) was kindly provided by Dr Pascal de Santa Barbara (Inserm, ERI25, Montpellier, France). Other primary antibodies were rabbit anti-β-galactosidase (Cappel, 1/500), mouse anti-β-galactosidase (Sigma Aldrich, 1/1000), goat anti-MYOCD (sc-21559, Santa-Cruz, 1/200), rat anti-HA (3F10 Roche, 1/1000), mouse anti-Flag M2 (F3165, Sigma Aldrich, 1/1000). Secondary antibodies used were Alexa-Fluor-546 or 488 donkey anti-rabbit, Alexa-Fluor-546 or 488-IgG_1_ goat anti-mouse, Alexa-Fluor-488 goat anti-guinea pig and Alexa-Fluor-555 goat anti-rat (Molecular Probes). Secondary antibodies were diluted at [1/1000]. Cultured cells were fixed in 1% PFA for 10 min. Cryosections and cultured cells were washed with 0.1% Tween/PBS for 15 min and then blocked in 10% foetal calf serum, 0.1% Tween/PBS for 1 hour. Fixed samples were incubated with primary antibodies overnight at 4°C, followed by incubation with secondary antibodies for 1 hour (RT) and then counterstained with DAPI.

*In situ* hybridisation using digoxigenin-labeled probes was performed on sections as previously described [21]. The *Myocd* probe was kindly provided by Dr Eric Olson (University of Texas Southwestern Medical Center, Dallas, USA).

### Construction of plasmids

The mouse *Myocd 856*-Flag pcDNA3.1 (smooth muscle *Myocardin* isoform, [22]) was kindly provided by Dr Joseph Miano (Aab Cardiovascular Research Institute, University of Rochester, USA). The human *Sox9*-Flag expression vector was provided by Dr Andreas Schedl (U638, Inserm, Nice, France). The Flag-*Sox9ΔC* pcDNA3.1 was given by Dr Benoit de Crombrugghe (University of Texas M. D. Anderson Cancer Center, Houston, USA). The mouse mammalian expression plasmid for serum response factor (*Srf*) was given by Dr Zhenlin Li (University Pierre and Marie Curie, Paris VII, France). The mouse *Myod 856*-HA, *Srf*-HA, *Sox9*-HA, and Flag-*Sox9HMG* were generated by PCR amplification and cloned into pcDNA3.1. The pcDNA3.1-HA-*Tshz3* and pCX-*Tshz3* clones were produced by subcloning a 3.4 kbp PCR-generated fragment covering the complete HA-*Tshz3* open reading frame into the pcDNA3.1(+) (Invitrogen) and the pCX-MCS2 (gift of Dr Xavier Morin, ENS, Paris, France) plasmids, respectively. An N-terminal 6XHis tag was ligated to *Tshz3* by PCR. *N-Tshz3* and C-*Tshz3* were cloned by PCR and ligated to pcDNA3.1. Human *TSHZ3*-trunc pCMV was a gift from Pr Joseph D. Buxbaum (Laboratory of Molecular Neuropsychiatry, Mount Sinai School of Medicine, New York, USA). *Tshz3*-trunc was cloned into the pcDNA3.1 vector in frame with an amino-terminal HA epitope. HA-*Tshz3* dZNF was initially generated in pBS SK vector by a QuikChange multi-site mutagenesis kit (Stratagene) and then transferred to the pcDNA3.1 vector. All the resulting plasmids were sequenced to check the insert integrity.

### mRNAs extraction and qRT-PCR

Before qRT-PCR analysis on cells, C3H10T1/2 cells were nucleofected using Amaxa^TM^ nucleofector Kit V (Lonza) following the manufacturer's protocols with designed program T-020. For qRT-PCR on ureters, embryonic ureters were dissected from E16.5 *Tbx18^Cre/+^Hprt^Sox9/+^* and wild type embryos. Total RNA was isolated using TRIsure Reagent (Bioline) according to the manufacturer's instructions. RNA was treated with RQ1 RNase-free DNase1 (Promega). 750 ng of total RNA was reverse-transcribed using Superscript II reverse transcriptase (Invitrogen) with oligo dT(12–18mers) and random primers (15mers). cDNA was submitted to RNAseH treatment during 20′ at 37°C to remove the complementary RNA strand. Real-time PCR was performed on an IQ5 System (Bio-Rad Laboratories) using SYBR Green Supermix (Invitrogen). qRT-PCR was performed for 40 cycles of 95°C for 30 s, 60°C for 30 s, and 72°C for 30 s. Analyses were performed in triplicate on RNA samples from several independent experiments. Results were normalised to *Gadph* housekeeping gene [23] and then normalised to their respective control group. RQ = 2^−ΔΔCt^ and ΔΔC_t_ =  (C_t experimental_ – C_t Gadph_) – (C_t control_ – C_t Gadph_).

Primer sequences used for Sybr qRT-PCR are as follows:

Mouse Tshz3 S-GCGCGCAGCAGCCTATGTTTC and

AS-TCAGCCATCCGGTCACTCGTC;

Mouse Myocd S-CGGATTCGAAGCTGTTGTCTT and

AS-AAACCAGGCCCCCTCCC;

Human Sox9 S-AACGCCTTCATGGTGTGG and

AS-TCGCTCTCGTTCAGAAGTCTC;

Mouse Sox9 S- CGAACGCACATCAAGACGG and

AS- CGGCTGCGTGACTGTAGTAGG;

Mouse SMaA S-CGCTGTCAGGAACCCTGAGA and

AS-CGAAGCCGGCCTTACAGA;

Mouse SMMHC S-TGGACACCATGTCAGGGAAA and

AS-ATGGACACAAGTGCTAAGCAGTCT;

Mouse telokin S-GACACCGCCTGAGTCCAACCTCCG and

AS-GACCCTGTTGAAGATTTCCTGCCACTG;

Mouse SM22 S-AGCCAGTGAAGGTGCCTGAGAAC and

AS-TGCCCAAAGCCATTAGAGTCCTC.

### GST protein-binding assays

*pGEX* plasmids encoding a GST fusion with TSHZ3 full length (TSHZ3fl), truncated TSHZ3 (N-TSHZ3, C-TSHZ3), SOX9 full length (Dr Pascal de Santa Barbara, Inserm, ERI25, Montpellier, France), MYOCD full length protein (gift from Dr Eric N Olson, University of Texas Southwestern Medical Center, Dallas, USA) were transformed into *E. coli* BL21. GST fusion proteins were isolated from cell lysates using standard protocols. The production of GST fusion proteins was induced by a 4-hour induction with 0.1 M IPTG at 25°C. Bacteria were lysed in STE (150 mM NaCl, 10 mM Tris pH 8, 1 mM EDTA), 1 mM DTT and 50 mg lysozyme, 1% Triton X-100. Resin-bound GST fusion proteins were pelleted, washed in the same buffer containing 0.5% Triton X-100. *Tshz3*fl, N-*Tshz3*, C-*Tshz3*, *Sox9* and *Myocd* were translated *in vitro* in presence of (^35^S) methionine. Glutathione beads conjugated with GST fusion protein pre-incubated in 1% BSA were incubated overnight at 4°C with radiolabeled proteins. Proteins were analyzed by SDS-polyacrylamide gel electrophoresis (PAGE) and subsequent autoradiography.

### Coimmunoprecipitation assays and Western blot

HEK293T cells were transiently transfected with plasmids encoding the Flag or HA-tagged MYOCD, HA-tagged TSHZ3, Flag-tagged SOX9, HA-SRF with Promofectin (Promocell). pcDNA3.1 parental plasmid was transfected to equalize the amount of DNA in each condition. For protein isolation, after 24 hours of culture, 100 mm plates of cells were scraped into 1 ml of PBS and centrifuged at 2000 rpm for 5 min. The cell pellet was immediately resuspended in 500 μl of lysis buffer containing 50 mM Tris-HCl pH 7.6, 200 mM NaCl, 1 mM EDTA, 0.25% Na-deoxycholate, 1 mM PMSF, 1 mM Na_3_VO_4_ and protease inhibitors (Complete; Roche). Cell extracts were incubated with anti-Flag agarose M2 beads (Sigma) or anti-HA (3F10, Roche) and protein G beads prewashed in lysis buffer according to the manufacturer's protocol. Samples were incubated overnight at 4°C on a rotator. For Western blot, total cellular protein was subjected to SDS/8% PAGE and then transferred to nylon membranes. 4% total extract was loaded as input. Immunoblotting was carried out using rat anti-HA (3F10, Roche) or mouse anti-Flag M2 (F1804, Sigma) antibodies, followed by secondary anti-rat HRP (Molecular Probes) or anti Mouse TrueBlot® ULTRA HRP (eBioscience) antibodies and detection using the Western Lightning plusTM ECL (Perkin Elmer).

### Mammalian expression and reporter gene assays

The mouse *telokin* promoter – luciferase reporter gene including nucleotides from −190 to + 181 (T370, telokin-Luc) of the *telokin* gene was a gift from Dr Paul Herring (Indiana University School of Medicine, Indianapolis, USA). The rat *SM α-actin* promoter fragment extends from nucleotide 2,555 to 2,813 and the rabbit *SMMHC* promoter from 4,200 to 11,600, the *SM22*–luciferase reporter gene includes nucleotides 475 to 61 of mouse *SM22* were generously provided by Pr Gary Owens (Molecular Physiology and Biological Physics, University of Virginia, USA). Plasmids were transfected into mouse C3H10T1/2 fibroblasts cells using FuGENE6 (Roche). 10T1/2 cells were seeded at 25000 cells/well in 24-well plates. Half an hour post-seeding, each dish was incubated with a total of 1 µg of plasmid DNA (0.125 µg of promoter-luciferase plasmid, 0.25 µg of expression plasmid, and 0.025 µg of phRLTK plasmid as an internal control) and 2 µl of FuGENE in 45 ml of Dulbecco's modified Eagle's medium. 24 h later, extracts (100 µl/well) were prepared for measurement of luciferase activity with Tristar LB941 multiplate reader (Berthold Technologies). The level of promoter activity was evaluated by determining the level of firefly luciferase activity relative to the control Renilla using the dual luciferase assay system essentially as described by the manufacturer (Promega). Results are reported as the mean and SEM.

### Statistical analyses

Statistical analyses were carried out using StatEL software for Mac OSX (www.adscience.eu). The non parametric Wilcoxon test was used to evaluate the significance of differences between control and tested samples. In all these analyses, P<0.05 or P<0.01 were regarded as significant.

## Results

### TSHZ3 and SOX9 form a molecular complex

**Figure 1:**
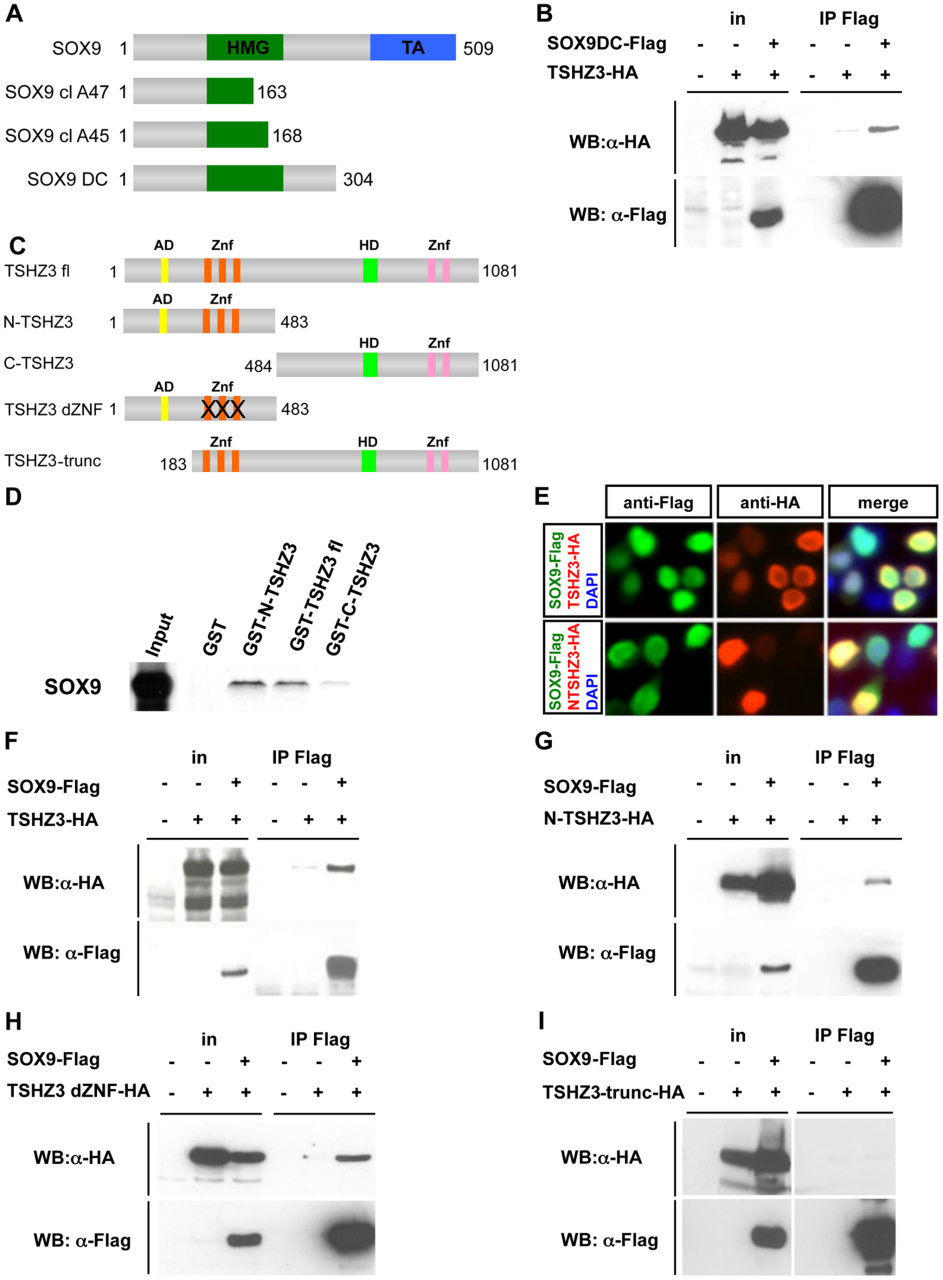
TSHZ3 and SOX9 physically interact *in vitro* and *in vivo*. (A–I) Mapping of the TSHZ3 interaction domain with SOX9. (A) Sequence analysis of the two *Sox9* clones clA47 (amino acids 1–163) and clA45 (amino acids 1–168) showed that the selected interaction domain corresponds to amino acids 1 to 163 of SOX9 that contains part of the HMG domain. The SOX9DC construct contains the HMG domain but not the transactivation (TA) domain (B) Coimmunoprecipitation experiment shows SOX9DC interacting with TSHZ3 protein. (C) Schematic structure of the TSHZ3 full length (TSHZ3 fl) and TSHZ3 truncated proteins used in this study. N-TSHZ3 harbours the N-terminal half of TSHZ3 (amino acid: 1–483), C-TSHZ3 harbours the C-terminal half of TSHZ3 (amino acid: 484–1081), TSHZ3 dZNF arbours N-terminal half of TSHZ3 (amino acid: 1–483) and mutated zinc finger motifs and, TSHZ3-trunc lacks the amino acids 1–182. AD  =  acidic domain; Znf  =  zinc finger domain; HD  =  homeodomain. (D) GST pulldown assays show that TSHZ3 interacts with *in vitro* translated SOX9. (E) TSHZ3-HA, N-TSHZ3-HA and SOX9-Flag localize to the nucleus in HEK293T transfected cells. Cells were counterstained with DAPI to detect nuclei. (F–I) HEK293T cells were transfected with HA-tagged TSHZ3 constructs and Flag-tagged SOX9 or control empty plasmids. Proteins were immunoprecipitated with a Flag antibody, followed by immunoblotting as indicated; in: input.

We recently reported on the important role of the transcription factor TSHZ3 for ureteric SM differentiation [11], but the molecular function of TSHZ3 in this developmental context remained unclear. Data obtained in invertebrate and vertebrate models showed that TSHZ-mediated gene regulation depends on interaction with different protein binding partners [24], [25], [26], [27]. To get insight into the molecular function of TSHZ3, we searched for protein interaction partners using a yeast-2-hybrid screen [20]. We identified 84 independent clones among which 47 (56%) clones encode for high mobility group (HMG) containing proteins, corresponding to SMARCE1/BAF57 (40 clones) [20] and five SOX proteins (7 clones). Sequence analysis of the 47 clones revealed that the selected interaction region contained an HMG domain. Two of the cDNAs (cl A47 and cl A45; Figure 1A) coded for SOX9 (Genbank accession number NM_011448.3), which has been implicated in SMC differentiation in the proximal ureter [10]. Consistent with the yeast two-hybrid data, co-immunoprecipitation assays performed in HEK293T cells using HA-epitope tagged TSHZ3 and Flag-epitope tagged N-terminal SOX9 (Sox9 DC; Figure 1A) showed that a fragment of SOX9 containing the HMG domain interacts with TSHZ3 (Figure 1B).

To delineate the region of TSHZ3 that accounts for SOX9 binding, we generated various *Tshz3* deletion constructs (Figure 1C) and performed a series of *in vitro* and *in vivo* binding assays (Figure 1D, F–I). We found that *in vitro* translated SOX9 binds to a fusion protein of full-length TSHZ3 (TSHZ3fl) with GST (GST-TSHZ3fl) but not to GST alone (Figure 1D). In a reciprocal manner, TSHZ3fl bound to GST-SOX9 fusion protein (Figure S1A). In these assays, SOX9 strongly interacted with GST-N-TSHZ3 harbouring the N-terminal half of TSHZ3 (amino acid: 1–483; N-TSHZ3) and weakly with the C-terminal half of TSHZ3 (amino acid: 484–1081; C-TSHZ3) (Figure 1D); N-TSHZ3 interacted with GST-SOX9 (Figure S1A). Next we performed coimmunoprecipitation (co-IP) assays in HEK293T cells transfected with a HA-tagged TSHZ3 construct alone, or in the presence of Flag-tagged SOX9. Tagged versions of both SOX9 and TSHZ3 localized to the nucleus of HEK cells (Figure 1E). An enrichment of HA-tagged TSHZ3 protein was detected only upon co-transfection of the *Sox9* expression construct (Figure 1F). The specificity of the association was confirmed by performing reciprocal co-IP assay with anti-HA antibody (Figure S1B). Co-IP assays confirmed that N-TSHZ3 interacts with SOX9 *in vivo* (Figure 1G) and showed that the zinc finger motif was dispensable for interaction (Figure 1H). The N-terminal deletion mutant of TSHZ3 (amino acid: 183–1081; TSHZ3-trunc) did not interact with SOX9 (Figure 1I). These data demonstrate that the interaction with SOX9 takes place within the first 182 amino acids of the TSHZ3 protein.

### SOX9 and TSHZ3 show overlapping expression in SM progenitors of the developing ureter

**Figure 2:**
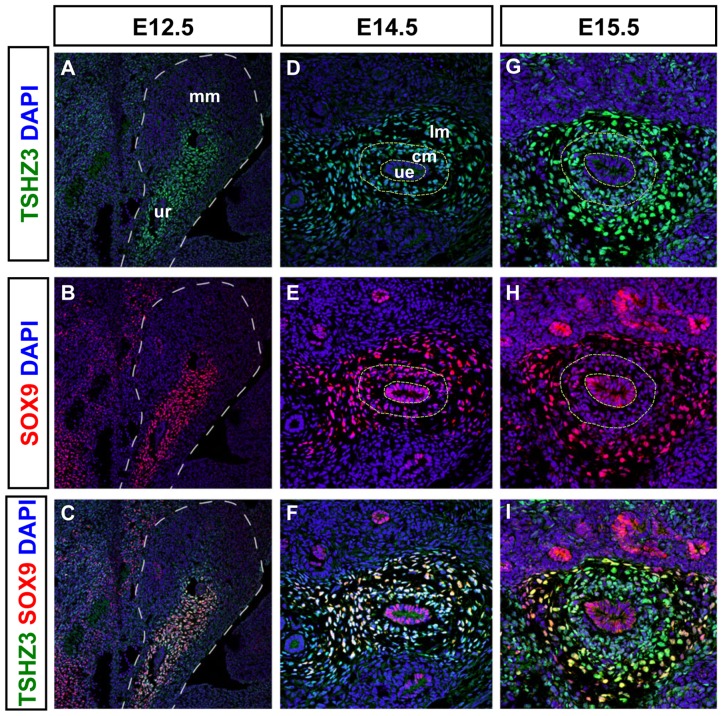
Comparative expression analysis of SOX9 and TSHZ3 in the developing ureter. (A–I) Immunostainings for SOX9 (red), TSHZ3 (green) on sagittal sections at E12.5, and on transverse sections of the ureter at E14.5 and E15.5. Nuclei were stained with DAPI (blue). (A, D, G) TSHZ3 antibody staining reveals mesenchymal expression in the ureter. (B, E, H) SOX9 antibody staining reveals a highly dynamic expression pattern in the developing ureter. (C, F, I) SOX9 expression overlaps with that of TSHZ3 in undifferentiated mesenchymal precursors from early stages whereas SOX9 signal decreases from E15.5 in the cm of the ureter. Broken line in A, B, C, D, E, G, H delineates metanephric mesenchyme (mm), ureter (ur), ureteric epithelium (ue), condensed ureteric mesenchyme (cm), loose ureteric mesenchyme (lm).

Genetic and molecular studies suggested that *Tshz3* and *Sox9* are required for the cytodifferentiation of the proximal ureteric mesenchyme into SM [10], [11]. These data together with our *in vitro* binding studies suggested that TSHZ3 and SOX9 interact molecularly in ureteric mesenchymal cells *in vivo*. To determine when this interaction takes place in the developing ureter, we performed a detailed analysis of the expression of TSHZ3 and SOX9 from E12.5 to E16.5 in wild-type (WT) ureters. From E12.5 to E14.5, TSHZ3 (Figure 2A, D) and SOX9 (Figure 2B, E) were co-expressed (Figure 2C, F) in the condensed mesenchyme (cm) around the epithelium (ue) as well in the outer ring of loose mesenchymal cells (lm). SOX9 was also detected in the epithelium, until E14.5 (Figure 2E). From E15.5 onwards, SOX9 expression was down-regulated in the condensed mesenchyme (Figure 2H, I) that undergoes SM differentiation [8], [28].

### Downregulation of SOX9 coincides with the activation of the expression of MYOCD target genes

**Figure 3:**
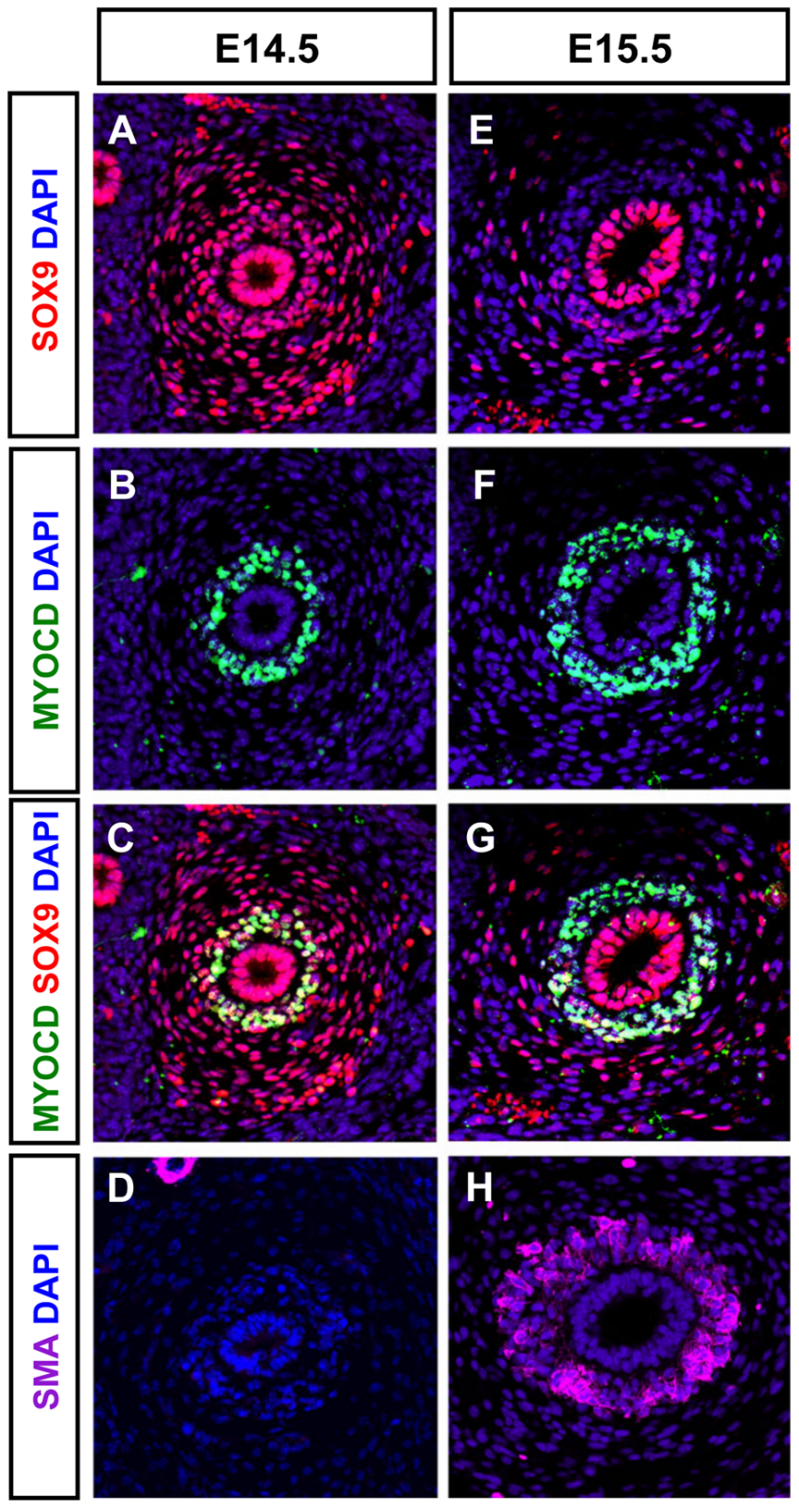
Expression of SOX9 in SM progenitors. (A–G) Transverse tissue sections of E14.5 (A–C) and E15.5 (E–G) proximal ureters showing co-immunostaining of SOX9 (red) and MYOCD (green) in the condensed mesenchyme at E14.5. (D, H) Smooth muscle α actin (SMaA, magenta) immunostaining is absent in E14.5 ureteric tissue section but is markedly expanded in the condensed mesenchymal compartment at E15.5.

The decrease of SOX9 protein at the onset of SMC differentiation prompted us to compare the expression of SOX9 with that of SM markers and MYOCD, a key transcriptional activator of several SM specific genes [13], [29], [30], [31]. In wild-type ureters at E14.5, we found that SOX9 and MYOCD proteins were co-detected in the SMC progenitors of WT ureters (Figure 3A–C), as supported by the absence of SMaA (Figure 3D). One day later at E15.5, the SOX9 level decreased and the protein was excluded from the MYOCD expression domain (Figure 3E–G); SMaA was now detected (Figure 3H). At E16.5, SOX9 was detected in the loose mesenchyme that will mature into connective tissue but SOX9 was absent from the SM layer (Figure S2H–J). In contrast to SOX9, expression of *Tshz3* was maintained from E15.5 onwards in the SM layer ([11]; Figure S2 C, D, F, G). As the expression of SM genes depends on the presence of the serum response factor (SRF), we confirmed that SRF was expressed at E14.5 and E15.5 in ureteric mesenchymal cells (Figure S2K–P). In the urogenital tract, the initiation of SM development is first detected in the presumptive bladder and 48 h later, it occurs in the developing ureter [28]. Accordingly, immunostaining of mouse embryonic urogenital tracts at E13.5 showed Myocardin staining in the bladder but not in the ureter (Figure S2 Q, R). This *in vivo* analysis temporally delineates the multistep process of the SM progenitor cell differentiation.

### TSHZ3 and SOX9 repress the myogenic activity of MYOCD

**Figure 4:**
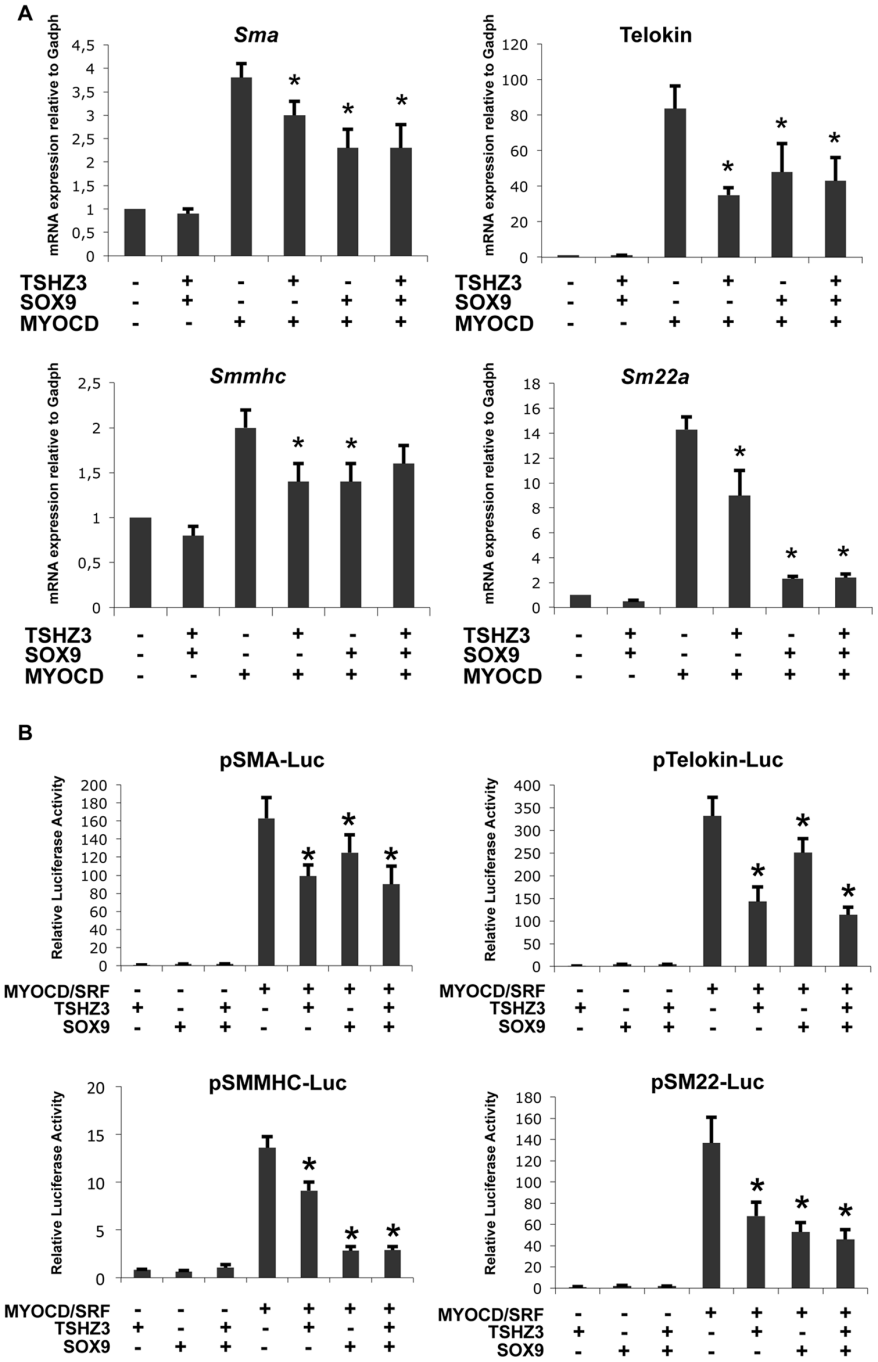
TSHZ3 and SOX9 modulate the transcriptional and myogenic activity of Myocardin. (A) Overexpression of *Sox9* and *Tshz3* suppresses the induction of endogenous SM gene expression by MYOCD. 10T1/2 cells were cotransfected with indicated combination of expression constructs. Cells were harvested 24 hours post nucleofection for qRT-PCR analysis of transcripts of endogenous *SMaA*, *telokin, SMMHC* and *SM22α? Gadph* was used to normalize (n = 6, mean ± S.EM). (B) SOX9 and TSHZ3 suppress the MYOCD and SRF-induced activation of SM specific promoters. 10T1/2 cells were cotransfected with indicated combination of expression constructs, luciferase reporters controlled by *SMaA*, *telokin, SMMHC and SM22α?* The amount of each plasmid was 250 ng for *Myocd*, *Sox9* and *Tshz3*, 10 ng for *Srf*, 125 ng for promoter reporter plasmid and 25 ng for PhRLTK renilla luciferase (n = 8, mean ± S.EM). *Asterisk* indicates statistical significance as determined by a Wilcoxon test (*p*<0.05).

A large number of studies established that MYOCD activates SM genes directly through an obligate interaction with SRF [32], [33]. Thus, we sought to analyse the consequence of the expression of TSHZ3 and SOX9 on the MYOCD/SRF-mediated transcriptional activation of SM-specific genes in more detail. This analysis was performed in the C3H10T1/2 fibroblastic cell line, which represents a relevant cellular model for studying SM differentiation [34], [35]. *Sox9* or *Tshz3* expression plasmids were transfected individually or in combination with or without *Myocd* expression plasmid in 10T1/2 cells. Evaluation of mRNA levels of SM genes by quantitative real-time PCR (qRT-PCR) showed that forced expression of TSHZ3 and SOX9 significantly diminished the MYOCD/SRF-mediated transcriptional activation of SM genes (Figure 4A). qRT-PCR analysis showed that the relative levels of exogenous *Myocd* mRNA were comparable in the different conditions, indicating that the observed repression was not related to variation of *Myocd* expression (Figure S3). Based on the above findings, we examined the effect of TSHZ3 and SOX9 on the luciferase activity of reporter plasmids containing the minimal promoters of a series of SM specific genes. We found that TSHZ3 and SOX9 potently repressed MYOCD/SRF-mediated luciferase activity of reporter plasmids (Figure 4B). We conclude that TSHZ3 and SOX9 interfere with the transcriptional activity of MYOCD/SRF.

### TSHZ3 interacts with MYOCD

**Figure 5:**
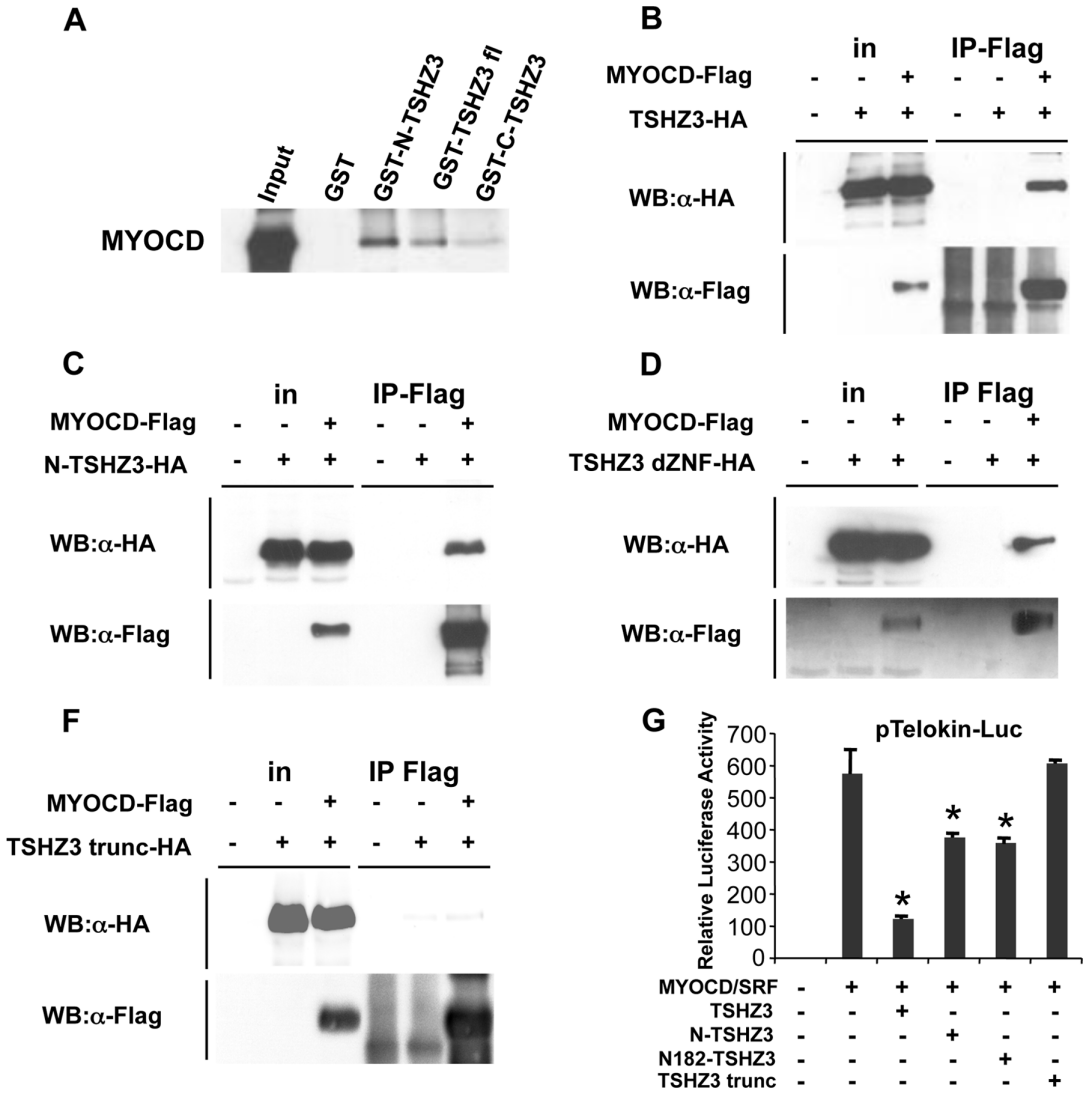
TSHZ3 and MYOCD physically interact *in vitro* and *in vivo*. (A–E) Mapping of the TSHZ3 interaction domain with MYOCD. (A) GST pulldown assays show TSHZ3 constructs interacting with *in vitro* translated MYOCD. (B–E) HEK293T cells transfected with HA-tagged TSHZ3 constructs and Flag-tagged MYOCD or control empty plasmids and then immunoprecipitated with a Flag antibody, followed by immunoblotting as indicated; in: input. (G) 10T1/2 cells were cotransfected with a luciferase reporter controlled by the *telokin* promoter and TSHZ3 constructs. TSHZ3-trunc-HA lost the ability to suppress the transcriptional activity of MYOCD/SRF (n = 8, mean ± SEM). *Asterisk* indicates statistical significance as determined by a Wilcoxon test (*p*<0.05).

The regulation of SM gene expression depends on the interaction of SRF with cofactors and the displacement of MYOCD from SRF can lead to the repression of SM genes [13], [36], [37]. We hypothesised that TSHZ3 and SOX9 modulate the transcriptional activity of MYOCD/SRF by interacting with either MYOCD or SRF. To test this hypothesis, we performed GST pull-down and coimmunoprecipitation assays. As shown in Figure 5A, TSHZ3 interacted efficiently with MYOCD. In contrast, SRF neither bound to SOX9 nor to TSHZ3 (not shown); and SOX9 did not bind to MYOCD (Figure S4A).

We mapped the MYOCD-binding domain of TSHZ3 in coimmunoprecipitation and GST pull-down assays using truncated constructs of TSHZ3. GST pull-down experiments showed that MYOCD interacts with the N-terminal half of TSHZ3 (Figure 5A and Figure S4B). To investigate the interaction of MYOCD and TSHZ3 further, we performed coimmunoprecipitation assays. HEK cells were transfected with expression plasmids for the SM isoform of MYOCD (Flag-tagged MYOCD-856, [22]), and TSHZ3-HA. We verified that MYOCD and TSHZ3 transfected proteins predominantly localized to the nucleus of HEK cells (Figure S4C). Specific interaction of MYOCD and TSHZ3 was observed by co-IP with anti-Flag antibody followed by immunoblotting with anti-HA (Figure 5B and Figure S4D). Co-IP assays showed that N-TSHZ3-HA and a TSHZ3 truncated protein lacking the three zinc finger domains (TSHZ3 dZNF-HA) bound to MYOCD-Flag (Figure 5C, D). Co-IP experiments revealed, in contrast, that a TSHZ3 N-terminal deletion mutant lacking residues 1–182 (TSHZ3-trunc-HA) lost both the ability to interact with MYOCD and to repress the transcriptional activity of MYOCD/SRF (Figure 5, E, F). Thus, the ability of TSHZ3 to inhibit SM gene promoter correlated with the ability to interact with MYOCD and was probably specific for MYOCD activity, as TSHZ3 did not affect SRF trans-activation properties (Figure S4E).

### TSHZ3 and SOX9 interfere with MYOCD/SRF complex formation

**Figure 6:**
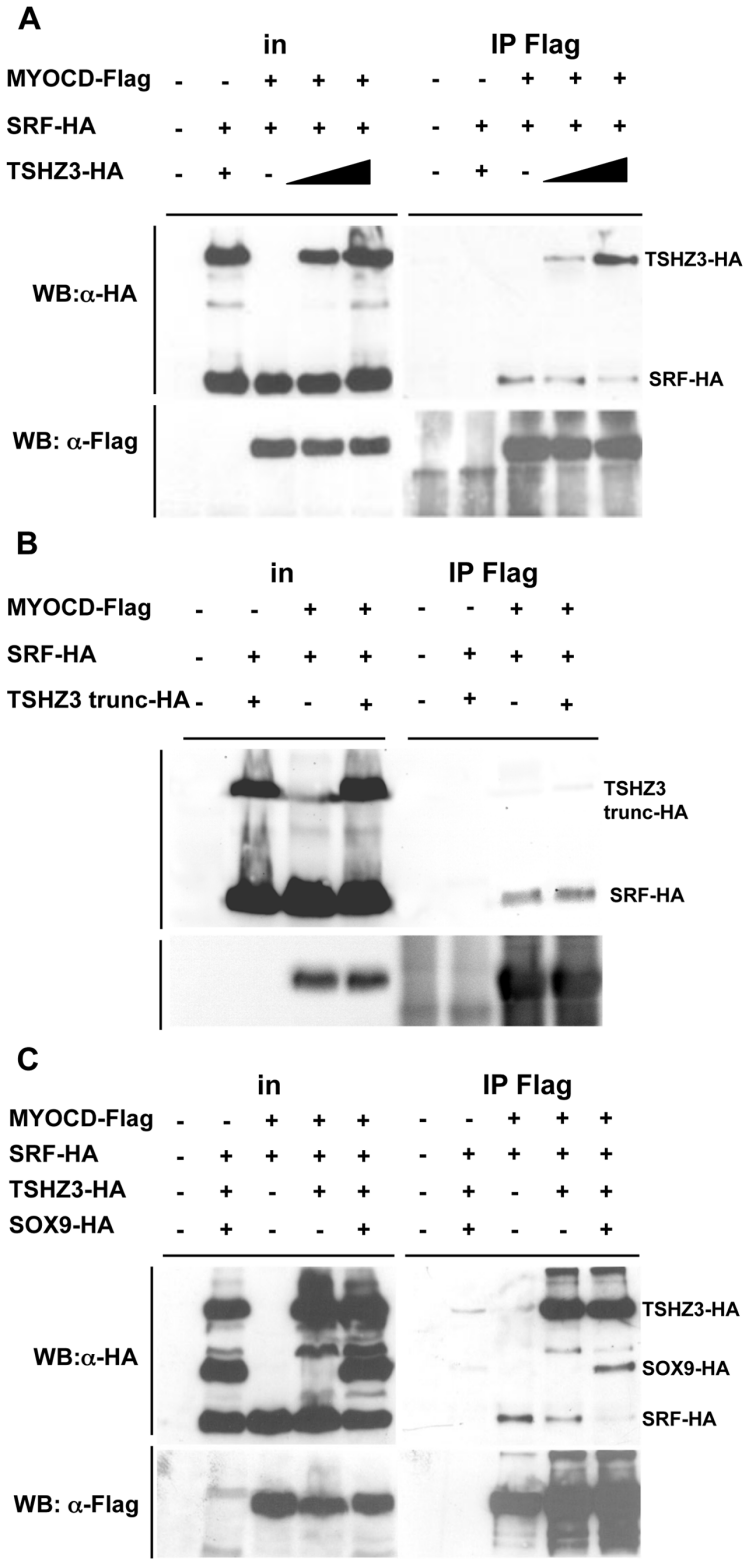
TSHZ3 and SOX9 compete with SRF for binding to MYOCD. (A–C) HEK293T cells were transfected with vectors encoding HA-SRF, HA-SOX9, HA-TSHZ3, HA-TSHZ3-trunc with Flag-MYOCD or control empty plasmid as indicated. HA-tagged proteins were identified by their molecular weight. Flag-MYOCD was immunoprecipitated (IP) from nuclear extracts of the transfected cells and co-precipitating HA-tagged proteins were detected by Western blotting.

As the displacement of MYOCD from SRF can lead to the repression of SM genes, we analysed whether TSHZ3 and SOX9 could affect the interaction of MYOCD with SRF [37]. We tested this hypothesis by performing co-IP experiments. We first determined the effect of TSHZ3 and found that the amount of immunoprecipitated SRF decreased with increasing amounts of TSHZ3 (Figure 6A). In addition, we found that the TSHZ3-trunc protein lacking the MYOCD interaction domain did not affect SRF binding to MYOCD (Figure 6B), suggesting that the direct interaction between TSHZ3 and MYOCD prevents the association of SRF with MYOCD. We next analyzed the effect of both SOX9 and TSHZ3 on MYOCD/SRF complex formation. We found that MYOCD was less efficient to immunoprecipitate SRF. In SOX9 transfected cells, SOX9-HA was detected in the immunoprecipitate, probably via its interaction with TSHZ3 (Figure 6C). Together, these results suggest that the presence SOX9 and TSHZ3 can displace MYOCD from SRF, and as a consequence, prevents the activation of SM genes.

### Prolonged expression of SOX9 in the ureteric mesenchyme does not prevent initiation of *Myocd* expression but reduces expression levels of SM genes

**Figure 7:**
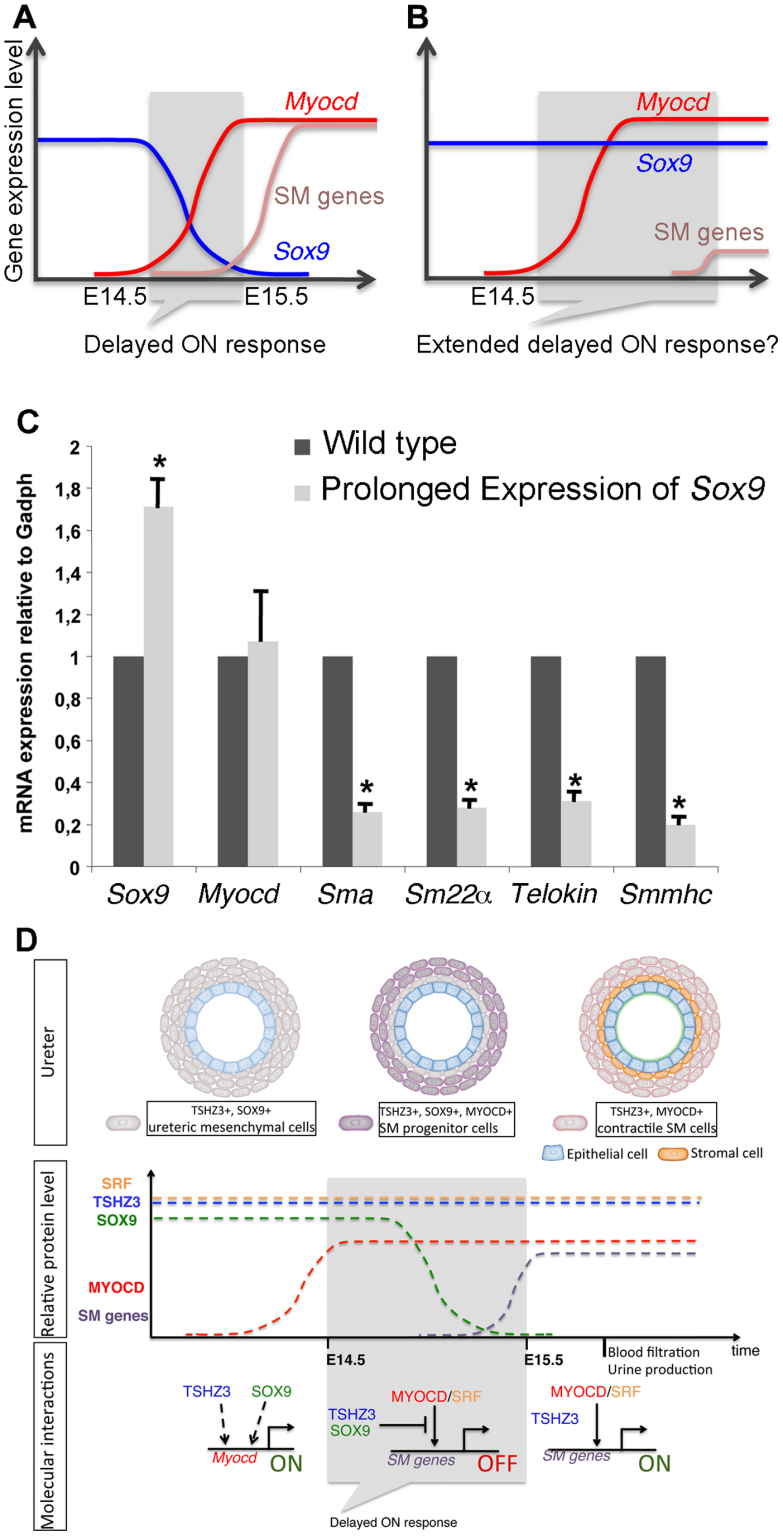
Model of timing of SM differentiation in the developing ureter. (A) Activation of the expression of SM genes correlates with the downregulation of *Sox9*. (B) *In vivo* and *in vitro* data suggest that prolonged expression of *Sox9* affects the transcriptional activation of SM genes but not the expression of *Myocd*. (C) Quantitative analysis of transcripts of *Sox9*, *Myocd* and SM markers in E16.5 wild-type and *Tbx18^Cre/+^;Hprt^Sox9/+^* (prolonged *Sox9*) dissected ureters. mRNA transcripts were normalized to *glyceraldehyde 3-phosphate dehydrogenase* (*Gadph*) (n = 7, mean ± SEM). (D) The upper panel shows the correlation between the status of ureteric mesenchymal cell differentiation, and the expression of the SM regulatory factors *Tshz3*, *Sox9* and *Myocd*. The middle panel schematizes the expression levels of these regulatory genes during ureter development. The bottom panel summarizes the molecular interactions and the molecular switch that precedes the expression of SM genes. In the undifferentiated mesenchyme *Tshz3* and *Sox9* are required for the expression of *Myocd*. SM progenitors express TSHZ3, SOX9 and MYOCD. Downregulation of SOX9 allows expression of SM genes, thus, a “functional switch” from embryonic ureteric tube to the contractile ureter.

Our *in vitro* data suggest that MYOCD is displaced from SRF in the presence of SOX9 and TSHZ3 (Figure 6C), and that expression of SM genes correlates with the downregulation of *Sox9 in vivo* (Figure 3 and 7A). If this was of functional importance, prolonged expression of *Sox9* should affect the transcriptional activation of MYOCD/SRF target genes without affecting the expression of *Myocd* (Figure 7B). To test this, *Sox9* expression was maintained in the ureteric mesenchyme beyond E14.5 using a conditional Cre/loxP-based *Sox9* misexpression approach [10], and expression of MYOCD/SRF target genes was measured. E16.5 ureters from wild-type and *Tbx18^Cre/+^;Hprt^Sox9/+^* mice were collected and the levels of *Myocd* and SM gene transcripts measured by qRT-PCR. We found that prolonged expression of *Sox9* significantly affects SM gene but not *Myocd* expression (Figure 7C), suggesting that down-regulation of *Sox9* is important to trigger the expression of SM genes and the progression of myogenic program.

## Discussion

*Myocd* encodes a transcriptional coactivator that physically associates with the MADS-box transcription factor, SRF, to promote myogenic differentiation. Expression and activity of *Myocd/*MYOCD is tightly controlled in space and time by a multitude of signaling pathways and transcriptional moieties in different SMC programs. Here, we have shown that in the ureteric mesenchyme the two transcription factors TSHZ3 and SOX9 control MYOCD transcriptional activity, thus, SMC differentiation, by ternary complex formation. Together with the previous findings that TSHZ3 and SOX9 are required for *Myocd* expression, this points to a crucial and dynamic function of these two transcription factors in ureteric SM development.

### A TSHZ/SOX9/MYOCD pathway controls SM differentiation of progenitor cells

The differentiation of ureteric mesenchyme into SMCs is sensitive to extracellular cues. In response to SHH from the ureteric epithelium, surrounding mesenchymal cells activate *Bmp4* [8]. TSHZ3 and SOX9 are likely to act downstream of BMP4 to trigger the initiation of the myogenic program through the transcriptional activation of *Myocd* [10], [11]. To date, a functional requirement for *Myocd* or *Srf* has not been established, but the absence of *Myocd* expression in *Tshz3*- and *Sox9*-mutant ureters, that show perturbed SMC differentiation, argue for a key role for *Myocd* in the initiation of the myogenic program [10], [11]. However, the precise nature of the molecular pathways regulating *Myocd* expression in the ureter remain poorly described and the regulatory regions responsible for transcriptional activation of *Myocd* have not been identified [22], [38]. In *Sox9*-deficient ureters, the expression of *Tshz3* is not affected [10], and in *Tshz3*-mutant ureters *Sox9* expression is unchanged (Figure S5), indicating that *Tshz3* and *Sox9* do not regulate each other. Physical interaction between TSHZ3 and SOX9 as identified in this study, may provide a clue for an upstream module for transcriptional activation of *Myocd*. This heterodimeric complex may directly activate *Myocd* transcription, alternatively it may provide a permissive environment for the expression of *Myocd*. Further characterization of *Tshz3-* and *Sox9*-mutant ureters may contribute to our understanding of the transcriptional regulation of *Myocd* in visceral SMC progenitors and the initiation of their differentiation program.

The results of this study indicate that TSHZ3 and SOX9 regulate the progression of the SM differentiation program by controlling the activity of MYOCD. The following observations support this conclusion. (1) TSHZ3 and SOX9 reduce the ability of MYOCD to activate expression of endogenous SM genes, as well as luciferase reporter gene controlled by SM gene promoter. (2) TSHZ3 and SOX9 form a ternary complex with MYOCD that strongly reduces the ability of MYOCD to interact with SRF. (3) Prolonged expression of *Sox9* prevents MYOCD to activate expression of endogenous SM genes. (4) TSHZ3 interacts directly with MYOCD and there is a positive correlation between the domain of TSHZ3 required for interaction with MYOCD and repression of SM gene expression. These data add to the notion that regulation of MYOCD activity provides another layer in the control mechanisms of SMC differentiation. They also explain why both ablation and overexpression of *Sox9* in ureteric mesenchymal cells affect SMC differentiation.

Based on these results, we propose a mechanistic model on the initiation and progression of ureteric SM differentiation by TSHZ3 and SOX9 (Figure 7C). TSHZ3 and SOX9 regulate the expression of *Myocd*, which is a critical step for the initiation of the myogenic program. Then TSHZ3 and SOX9 form a ternary complex with MYOCD, which prevents MYOCD from activating SM specific genes. The subsequent down-regulation of *Sox9* expression allows the interaction between MYOCD and SRF to activate the expression of SM genes. Interestingly, analysis of *Shh*-mutant ureters has led to the hypothesis that SM progenitors require a signal for differentiation, suggesting that in absence of this signal SM progenitors do not differentiate [8]. One possibility could be that the differentiation signal triggers the downregulation of *Sox9* expression. In absence of the signal, inhibition of *MYOCD* activity by TSHZ3 and SOX9 may maintain a progenitor-like state. Clearly, identification of this signal through which downregulation of *Sox9* occurs is critical and would represent a major advance in understanding the mechanisms underpinning ureteric SM differentiation.

Cellular and biochemical experiments suggest that despite the presence of the potent activator MYOCD, TSHZ3 and SOX9 impair the activation of SM specific genes, favoring the maintenance of a progenitor state. In vivo, there is a close correlation between the downregulation of *Sox9* and the initiation of the expression of SRF/MYOCD target genes. Prolonged expression of S*ox9* clearly provided *in vivo* evidence for the inhibitory role of *Sox9* in visceral myogenesis. Of note, the correlation between the downregulation of *Sox9* and the mesenchymal cell differentiation has previously been described in osteoblast differentiation [39]. In skeletal muscles, it has been proposed that *Tshz3* and *Sox9* might be responsible for maintaining myoblasts in an undifferentiated state and prevent differentiation into myotubes. Notably, in the presence of TSHZ3 or SOX9, MYOD is unable to activate the *myogenin* promoter [40], [41].

These data suggest that *Tshz3* and *Sox9* direct ureteric mesenchymal cells to the visceral SM lineage but *Sox9* inhibits differentiation of SM progenitor cells into mature SM.

### Similar strategies in vascular and visceral myogenic programs

We identified a dynamic transcriptional network, in which transcription factors are required in a first step to initiate the expression of *Myocd* and in a second step to trigger the progression of the myogenic program by controlling the activity of MYOCD. This two-step mechanism bears similarity with a mechanism previously described for vascular smooth and cardiac muscles, where the FOXO4 factor participates in the transcriptional activation of *Myocd* and FOXO4 can interact with and repress the transcriptional activity of MYOCD [22], [42]. It has been proposed that the interaction between FOXO4 and MYOCD might help to fine tune the potent transcriptional activity of MYOCD [22]. The capacity of TSHZ3 to modulate the activity of MYOCD/SRF suggests that in visceral SMC TSHZ3 might help to fine-tune the transcriptional activity of MYOCD at late stages of ureter development.

Based on current knowledge this model might not occur in other context of visceral SM development. In the future, it would be important to consider whether the combinatorial action of TSHZ3 and SOX9 could apply to other TSHZ and SOX factors.

## Supporting Information

Figure S1 **TSHZ3 and SOX9 form a complex** ***in vivo*** **and** ***in vitro*** **.** (A) GST-SOX9 or GST alone were incubated with N-TSHZ3, TSHZ3fl, or C-TSHZ3 *in vitro* expressed in reticulocyte. (B) HEK cells were transfected with Flag-tagged SOX9 and HA-tagged TSHZ3. TSHZ3 proteins were immunoprecipitated using anti-HA antibody, as indicated. Immunoprecipitated proteins were identified by Western blotting using antibodies against the HA and Flag epitopes.
(TIF)

Figure S2 **Maintenance of TSHZ3 protein in ureteric SM progenitors and SM cells, and loss of SOX9 detection in SM cells at E16.5.** (A–D) Transverse sections of proximal ureters showing β-galactosidase immunostaining (brown), which recapitulates TSHZ3 expression, and expression of *myocardin* by *in situ* hybridization (purple signal) at E14.5 and E15.5. (E–J) Detection of THSZ3 protein (green, F, G) contrasting with absence of SOX9 (red, I, J) in SM cells stained with the SM marker: SMA (magenta, E, H) on transverse sections of ureters at E16.5. (K–P) Detection of SRF (green, K, M, N, P) and SMA (magenta, L, M, O, P) on transverse sections of ureters at E14.5 (K–M) and E15.5 (N–P). (Q, R) Expression of Myocardin in the ureter and the bladder at E13.5. Q and R, Immunostaining for Myocardin (red) and nuclei (DAPI, Blue). Myocd was not detected in the ureteral mesenchyme (A), in contrast to the bladder (B). Dotted line in A separates the epithelial layer and the mesenchymal layer. Asterisk indicates the lumen of the ureter.
(TIF)

Figure S3 **Myocd expression is stable.** 10T1/2 cells were cotransfected with the indicated constructs. 24 hours post nucleofection, total RNA was harvested from cells and expression levels of exogenous *Myocd* were measured by qRT-PCR.
(TIF)

Figure S4 **TSHZ3 interacts with MYOCD.** (A) HEK cells were transfected with expression vectors encoding Flag-tagged SOX9 and HA-tagged MYOCD. Cell lysates were incubated with anti-Flag (SOX9) antibody, and the immunoprecipitates were probed with anti-HA (MYOCD) or anti-Flag (SOX9) antibodies by Western analysis. (B) Aliquots of in vitro translated 35S-labelled full-length (TSHZ3 fl) and deletion mutants of TSHZ3 (N-TSHZ3, C-TSHZ3) were incubated with glutathionne-Sepharose beads loaded with bacterially expressed GST alone or GST-MYOCD. The retained 35S-labeled TSHZ3 proteins were separated on SDS-PAGE followed by autoradiography. 10% of input (in) is shown on the left. In control experiments, no binding of TSHZ3 proteins to the GST beads was observed. (C) TSHZ3-HA (red, anti-HA epitope tag), N-TSHZ3-HA (red) and MYOCD-Flag (green, anti-Flag epitope tag) localize to the nucleus in HEK293T transfected cells. Cells were counterstained with DAPI to detect nuclei. (D) HEK cells were transfected with expression vectors encoding Flag-tagged MYOCD and HA-tagged TSHZ3. Cell lysates were incubated with anti-HA (TSHZ3) antibody, and the immunoprecipitates were probed with anti-HA (TSHZ3) or anti-Flag (MYOCD) antibodies by Western analysis. (E) 10T1/2 with indicated combination of expression constructs and a luciferase reporter controlled by *SMaA*. Luciferase activity was measured. SRF significantly activated SMA luciferase promoter around 1,5 fold relative to the basal activity of SMA promoter alone. This activation was unaffected by co-transfection with TSHSZ3. An arbitrary value of 1 was assigned to the basal activity of SMA promoter alone. (n = 12, mean ± S.EM).
(TIF)

Figure S5 **Expression of** ***Sox9*** **is not affected in** ***Tshz3*** **mutant ureters.** (A, B) *Sox9* expression in wild type ureter. (C, D) *Sox9* expression in *Tshz3* mutant ureter.
(TIF)

## References

[1] Brenner-Anantharam A, Cebrian C, Guillaume R, Hurtado R, Sun TT, et al (2007) Tailbud-derived mesenchyme promotes urinary tract segmentation via BMP4 signaling. Development 134: 1967–1975. 17442697 10.1242/dev.004234

[2] Cain JE, Hartwig S, Bertram JF, Rosenblum ND (2008) Bone morphogenetic protein signaling in the developing kidney: present and future. Differentiation 76: 831–842. 18331343 10.1111/j.1432-0436.2008.00265.x

[3] Carroll TJ, Park JS, Hayashi S, Majumdar A, McMahon AP (2005) Wnt9b plays a central role in the regulation of mesenchymal to epithelial transitions underlying organogenesis of the mammalian urogenital system. Dev Cell 9: 283–292. 16054034 10.1016/j.devcel.2005.05.016

[4] Michos O, Goncalves A, Lopez-Rios J, Tiecke E, Naillat F, et al (2007) Reduction of BMP4 activity by gremlin 1 enables ureteric bud outgrowth and GDNF/WNT11 feedback signalling during kidney branching morphogenesis. Development 134: 2397–2405. 17522159 10.1242/dev.02861

[5] Miyazaki Y, Oshima K, Fogo A, Hogan BL, Ichikawa I (2000) Bone morphogenetic protein 4 regulates the budding site and elongation of the mouse ureter. J Clin Invest 105: 863–873. 10749566 10.1172/JCI8256 PMC377476

[6] Raatikainen-Ahokas A, Hytonen M, Tenhunen A, Sainio K, Sariola H (2000) BMP-4 affects the differentiation of metanephric mesenchyme and reveals an early anterior-posterior axis of the embryonic kidney. Dev Dyn 217: 146–158. 10706139 10.1002/(SICI)1097-0177(200002)217:2<146::AID-DVDY2>3.0.CO;2-I

[7] Wang GJ, Brenner-Anantharam A, Vaughan ED, Herzlinger D (2009) Antagonism of BMP4 signaling disrupts smooth muscle investment of the ureter and ureteropelvic junction. J Urol 181: 401–407. 19010499 10.1016/j.juro.2008.08.117 PMC4471861

[8] Yu J, Carroll TJ, McMahon AP (2002) Sonic hedgehog regulates proliferation and differentiation of mesenchymal cells in the mouse metanephric kidney. Development 129: 5301–5312. 12399320 10.1242/dev.129.22.5301

[9] Airik R, Bussen M, Singh MK, Petry M, Kispert A (2006) Tbx18 regulates the development of the ureteral mesenchyme. J Clin Invest 116: 663–674. 16511601 10.1172/JCI26027 PMC1386107

[10] Airik R, Trowe MO, Foik A, Farin HF, Petry M, et al (2010) Hydroureternephrosis due to loss of Sox9-regulated smooth muscle cell differentiation of the ureteric mesenchyme. Hum Mol Genet 19: 4918–4929. 20881014 10.1093/hmg/ddq426

[11] Caubit X, Lye CM, Martin E, Core N, Long DA, et al (2008) Teashirt 3 is necessary for ureteral smooth muscle differentiation downstream of SHH and BMP4. Development 135: 3301–3310. 18776146 10.1242/dev.022442

[12] Nie X, Sun J, Gordon RE, Cai CL, Xu PX (2010) SIX1 acts synergistically with TBX18 in mediating ureteral smooth muscle formation. Development 137: 755–765. 20110314 10.1242/dev.045757 PMC2827686

[13] Wang D, Chang PS, Wang Z, Sutherland L, Richardson JA, et al (2001) Activation of cardiac gene expression by myocardin, a transcriptional cofactor for serum response factor. Cell 105: 851–862. 11439182 10.1016/s0092-8674(01)00404-4

[14] Lye CM, Fasano L, Woolf AS (2010) Ureter myogenesis: putting Teashirt into context. J Am Soc Nephrol 21: 24–30. 19926888 10.1681/ASN.2008111206

[15] Reginensi A, Clarkson M, Neirijnck Y, Lu B, Ohyama T, et al (2011) SOX9 controls epithelial branching by activating RET effector genes during kidney development. Hum Mol Genet 20: 1143–1153. 21212101 10.1093/hmg/ddq558 PMC3809456

[16] dell'Agnola CA, Carmassi LM, Merlo D, Tadini B (1990) Duration and severity of congenital hydronephrosis as a cause of smooth muscle deterioration in pyelo-ureteral junction obstruction. Z Kinderchir 45: 286–290. 2284874 10.1055/s-2008-1042602

[17] Gunn TR, Mora JD, Pease P (1995) Antenatal diagnosis of urinary tract abnormalities by ultrasonography after 28 weeks' gestation: incidence and outcome. Am J Obstet Gynecol 172: 479–486. 7856673 10.1016/0002-9378(95)90560-x

[18] Ismaili K, Hall M, Piepsz A, Wissing KM, Collier F, et al (2006) Primary vesicoureteral reflux detected in neonates with a history of fetal renal pelvis dilatation: a prospective clinical and imaging study. J Pediatr 148: 222–227. 16492433 10.1016/j.jpeds.2005.09.037

[19] Zhang PL, Peters CA, Rosen S (2000) Ureteropelvic junction obstruction: morphological and clinical studies. Pediatr Nephrol 14: 820–826. 10955936 10.1007/s004679900240

[20] Faralli H, Martin E, Core N, Liu QC, Filippi P, et al (2011) Teashirt-3, a novel regulator of muscle differentiation, associates with BRG1-associated factor 57 (BAF57) to inhibit myogenin gene expression. The Journal of biological chemistry 286: 23498–23510. 21543328 10.1074/jbc.M110.206003 PMC3123113

[21] Caubit X, Tiveron MC, Cremer H, Fasano L (2005) Expression patterns of the three Teashirt-related genes define specific boundaries in the developing and postnatal mouse forebrain. J Comp Neurol 486: 76–88. 15834955 10.1002/cne.20500

[22] Creemers EE, Sutherland LB, McAnally J, Richardson JA, Olson EN (2006) Myocardin is a direct transcriptional target of Mef2, Tead and Foxo proteins during cardiovascular development. Development 133: 4245–4256. 17021041 10.1242/dev.02610

[23] Iwanaga Y, Kihara Y, Takenaka H, Kita T (2006) Down-regulation of cardiac apelin system in hypertrophied and failing hearts: Possible role of angiotensin II-angiotensin type 1 receptor system. J Mol Cell Cardiol 41: 798–806. 16919293 10.1016/j.yjmcc.2006.07.004

[24] Gallet A, Angelats C, Kerridge S, Therond PP (2000) Cubitus interruptus-independent transduction of the Hedgehog signal in Drosophila. Development 127: 5509–5522. 11076770 10.1242/dev.127.24.5509

[25] Kajiwara Y, Akram A, Katsel P, Haroutunian V, Schmeidler J, et al (2009) FE65 binds Teashirt, inhibiting expression of the primate-specific caspase-4. PLoS One 4: e5071. 19343227 10.1371/journal.pone.0005071 PMC2660419

[26] Onai T, Matsuo-Takasaki M, Inomata H, Aramaki T, Matsumura M, et al (2007) XTsh3 is an essential enhancing factor of canonical Wnt signaling in Xenopus axial determination. EMBO J 26: 2350–2360. 17431396 10.1038/sj.emboj.7601684 PMC1864982

[27] Taghli-Lamallem O, Gallet A, Leroy F, Malapert P, Vola C, et al (2007) Direct interaction between Teashirt and Sex combs reduced proteins, via Tsh's acidic domain, is essential for specifying the identity of the prothorax in Drosophila. Dev Biol 307: 142–151. 17524390 10.1016/j.ydbio.2007.04.028

[28] McHugh KM (1995) Molecular analysis of smooth muscle development in the mouse. Dev Dyn 204: 278–290. 8573719 10.1002/aja.1002040306

[29] Chen J, Kitchen CM, Streb JW, Miano JM (2002) Myocardin: a component of a molecular switch for smooth muscle differentiation. Journal of molecular and cellular cardiology 34: 1345–1356. 12392995 10.1006/jmcc.2002.2086

[30] Huang J, Cheng L, Li J, Chen M, Zhou D, et al (2008) Myocardin regulates expression of contractile genes in smooth muscle cells and is required for closure of the ductus arteriosus in mice. J Clin Invest 118: 515–525. 18188448 10.1172/JCI33304 PMC2176191

[31] Li S, Wang DZ, Wang Z, Richardson JA, Olson EN (2003) The serum response factor coactivator myocardin is required for vascular smooth muscle development. Proc Natl Acad Sci U S A 100: 9366–9370. 12867591 10.1073/pnas.1233635100 PMC170924

[32] Wang DZ, Li S, Hockemeyer D, Sutherland L, Wang Z, et al (2002) Potentiation of serum response factor activity by a family of myocardin-related transcription factors. Proc Natl Acad Sci U S A 99: 14855–14860. 12397177 10.1073/pnas.222561499 PMC137508

[33] Wang Z, Wang DZ, Pipes GC, Olson EN (2003) Myocardin is a master regulator of smooth muscle gene expression. Proc Natl Acad Sci U S A 100: 7129–7134. 12756293 10.1073/pnas.1232341100 PMC165841

[34] Hirschi KK, Rohovsky SA, D'Amore PA (1998) PDGF, TGF-beta, and heterotypic cell-cell interactions mediate endothelial cell-induced recruitment of 10T1/2 cells and their differentiation to a smooth muscle fate. J Cell Biol 141: 805–814. 9566978 10.1083/jcb.141.3.805 PMC2132737

[35] Zhou J, Herring BP (2005) Mechanisms responsible for the promoter-specific effects of myocardin. J Biol Chem 280: 10861–10869. 15657056 10.1074/jbc.M411586200

[36] Du KL, Ip HS, Li J, Chen M, Dandre F, et al (2003) Myocardin is a critical serum response factor cofactor in the transcriptional program regulating smooth muscle cell differentiation. Molecular and Cellular Biology 23: 2425–2437. 12640126 10.1128/MCB.23.7.2425-2437.2003 PMC150745

[37] Wang Z, Wang DZ, Hockemeyer D, McAnally J, Nordheim A, et al (2004) Myocardin and ternary complex factors compete for SRF to control smooth muscle gene expression. Nature 428: 185–189. 15014501 10.1038/nature02382

[38] Zacharias WJ, Madison BB, Kretovich KE, Walton KD, Richards N, et al.. (2011) Hedgehog signaling controls homeostasis of adult intestinal smooth muscle. Dev Biol. 10.1016/j.ydbio.2011.04.025 PMC3118277 21545794

[39] Karsenty G (2008) Transcriptional control of skeletogenesis. Annu Rev Genomics Hum Genet 9: 183–196. 18767962 10.1146/annurev.genom.9.081307.164437

[40] Schmidt K, Glaser G, Wernig A, Wegner M, Rosorius O (2003) Sox8 is a specific marker for muscle satellite cells and inhibits myogenesis. J Biol Chem 278: 29769–29775. 12782625 10.1074/jbc.M301539200

[41] Faralli H, Martin E, Core N, Liu QC, Filippi P, et al.. (2011) Teashirt-3, a novel regulator of muscle differentiation, associates with BRG-1 associated factor 57 (BAF57) to inhibit myogenin gene expression. J Biol Chem. 10.1074/jbc.M110.206003 PMC3123113 21543328

[42] Liu ZP, Wang Z, Yanagisawa H, Olson EN (2005) Phenotypic modulation of smooth muscle cells through interaction of Foxo4 and myocardin. Dev Cell 9: 261–270. 16054032 10.1016/j.devcel.2005.05.017

