# Expression of Concern: TSHZ3 and SOX9 Regulate the Timing of Smooth Muscle Cell Differentiation in the Ureter by Reducing Myocardin Activity

**DOI:** 10.1371/journal.pone.0211924

**Published:** 2019-02-11

**Authors:** The PLOS ONE Editors

Following publication of this article [1], several concerns were raised about the Western blots in Figs 1, 5, and 6. The authors confirmed that in preparing these figures they had spliced image fragments to remove empty lanes, rearrange the sample order, and in some cases to combine lanes from short and long exposures of a given blot. The Director of the Institute for Developmental Biology of Marseille discussed this matter with the corresponding author and examined the original data underlying the results in question. The Director concluded that the images were modified for the purpose of presentation, and that the scientific results presented in the article and underlying data are sound.

**Fig 1:**
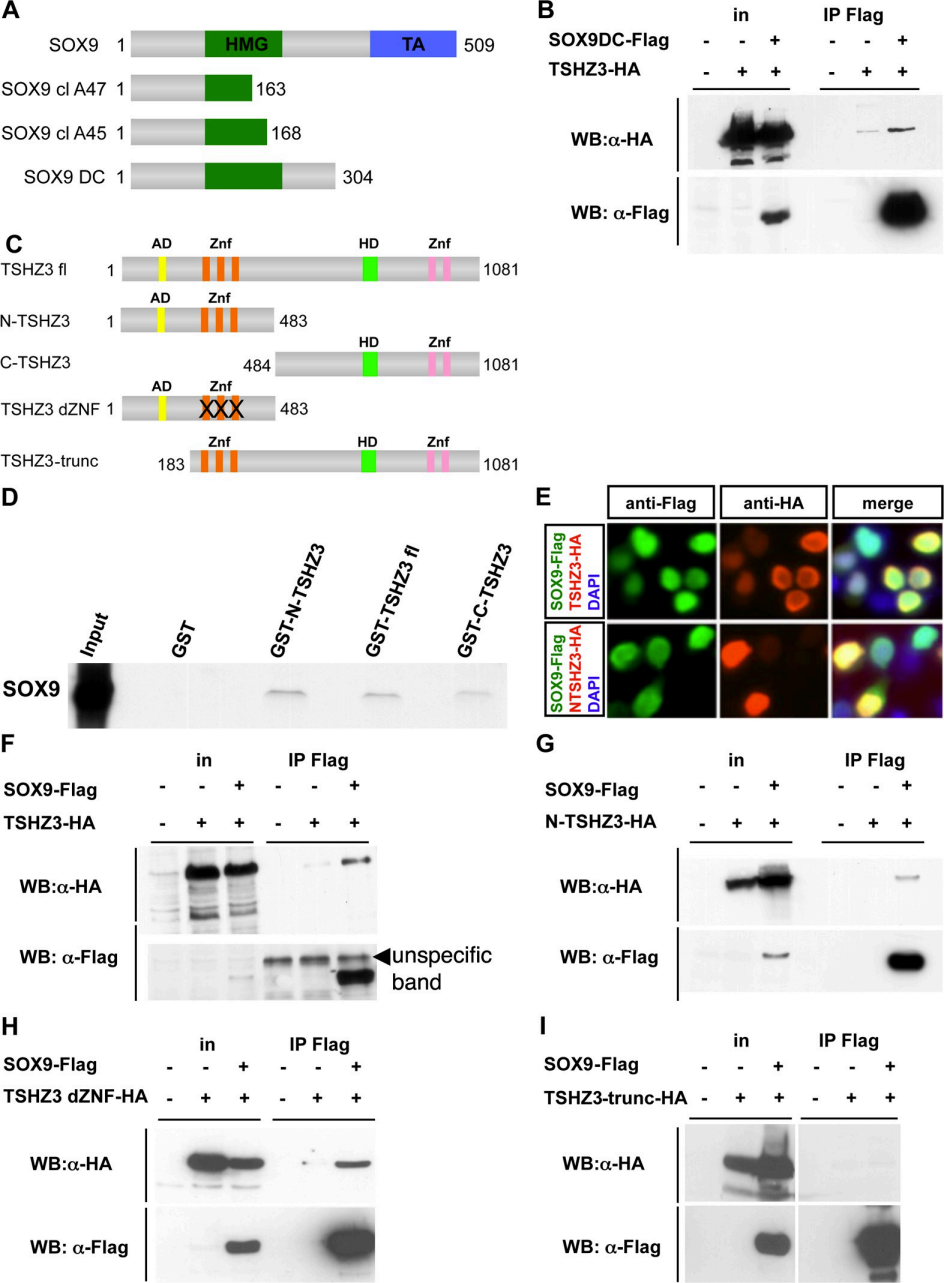
TSHZ3 and SOX9 physically interact *in vitro* and *in vivo*. (A-I) Mapping of the TSHZ3 interaction domain with SOX9. (A) Sequence analysis of the two *Sox9* clones clA47 (amino acids 1–163) and clA45 (amino acids 1–168) showed that the selected interaction domain corresponds to amino acids 1 to 163 of SOX9 that contains part of the HMG domain. The SOX9DC construct contains the HMG domain but not the transactivation (TA) domain (B) Coimmunoprecipitation experiment shows SOX9DC interacting with TSHZ3 protein. (C) Schematic structure of the TSHZ3 full length (TSHZ3 fl) and TSHZ3 truncated proteins used in this study. N-TSHZ3 harbours the N-terminal half of TSHZ3 (amino acid: 1–483), C-TSHZ3 harbours the C-terminal half of TSHZ3 (amino acid: 484–1081), TSHZ3 dZNF harbours N-terminal half of TSHZ3 (amino acid: 1–483) and mutated zinc finger motifs and, TSHZ3-trunc lacks the amino acids 1–182. AD = acidic domain; Znf = zinc finger domain; HD = homeodomain. (D) GST pulldown assays show that TSHZ3 interacts with *in vitro* translated SOX9. (E) TSHZ3-HA, N-TSHZ3-HA and SOX9-Flag localize to the nucleus in HEK293T transfected cells. Cells were counterstained with DAPI to detect nuclei. (F-I) HEK293T cells were transfected with HA-tagged TSHZ3 constructs and Flag-tagged SOX9 or control empty plasmids. Proteins were immunoprecipitated with a Flag antibody, followed by immunoblotting as indicated; in: input.

**Fig 5:**
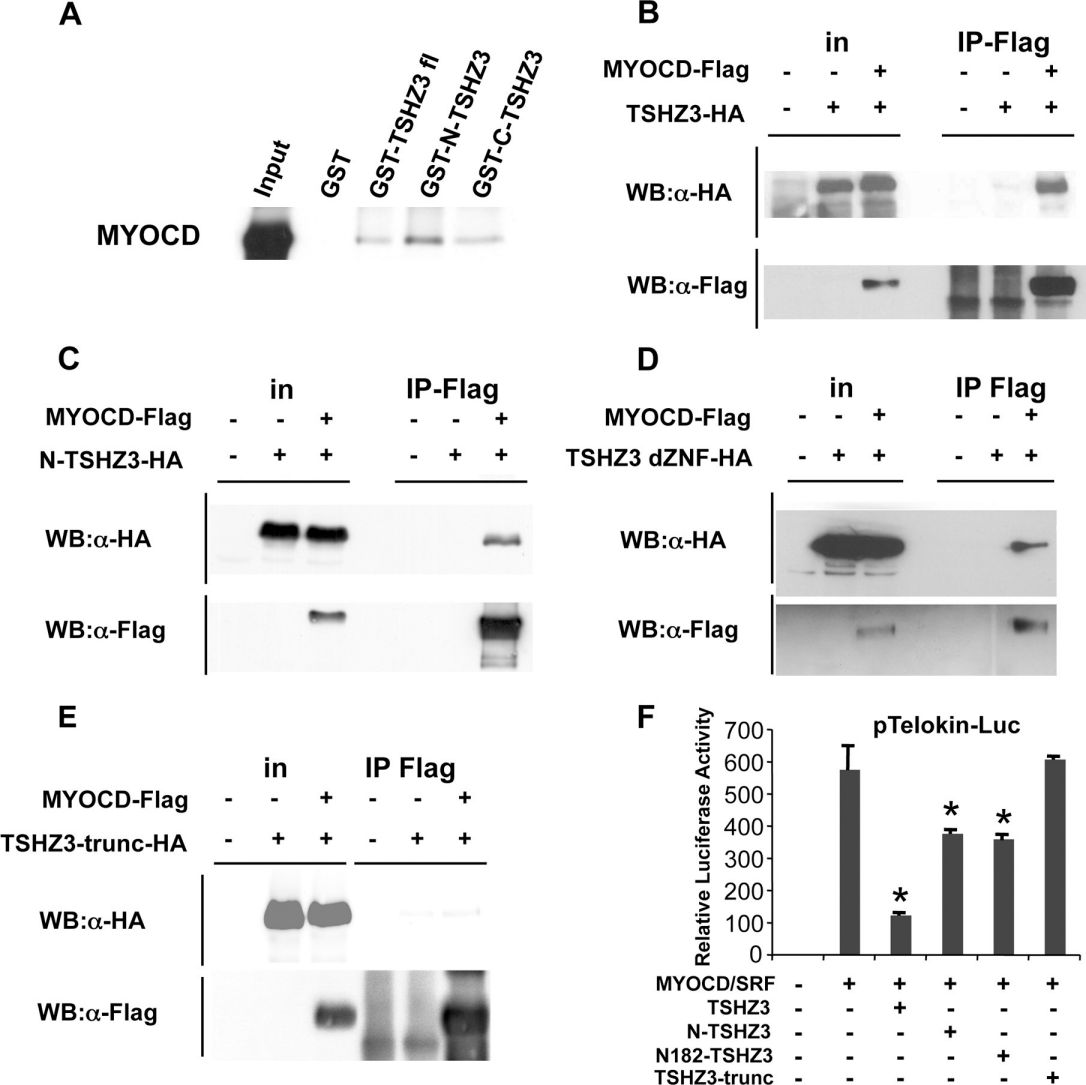
TSHZ3 and MYOCD physically interact *in vitro* and *in vivo*. (A-E) Mapping of the TSHZ3 interaction domain with MYOCD. (A) GST pulldown assays show TSHZ3 constructs interacting with *in vitro* translated MYOCD. (B-E) HEK293T cells transfected with HA-tagged TSHZ3 constructs and Flag-tagged MYOCD or control empty plasmids and then immunoprecipitated with a Flag antibody, followed by immunoblotting as indicated; in: input. (F) 10T1/2 cells were cotransfected with a luciferase reporter controlled by the *telokin* promoter and TSHZ3 constructs. TSHZ3-trunc-HA lost the ability to suppress the transcriptional activity of MYOCD/SRF (n = 8, mean ± SEM). *Asterisk* indicates statistical significance as determined by a Wilcoxon test (*p*<0.05).

**Fig 6:**
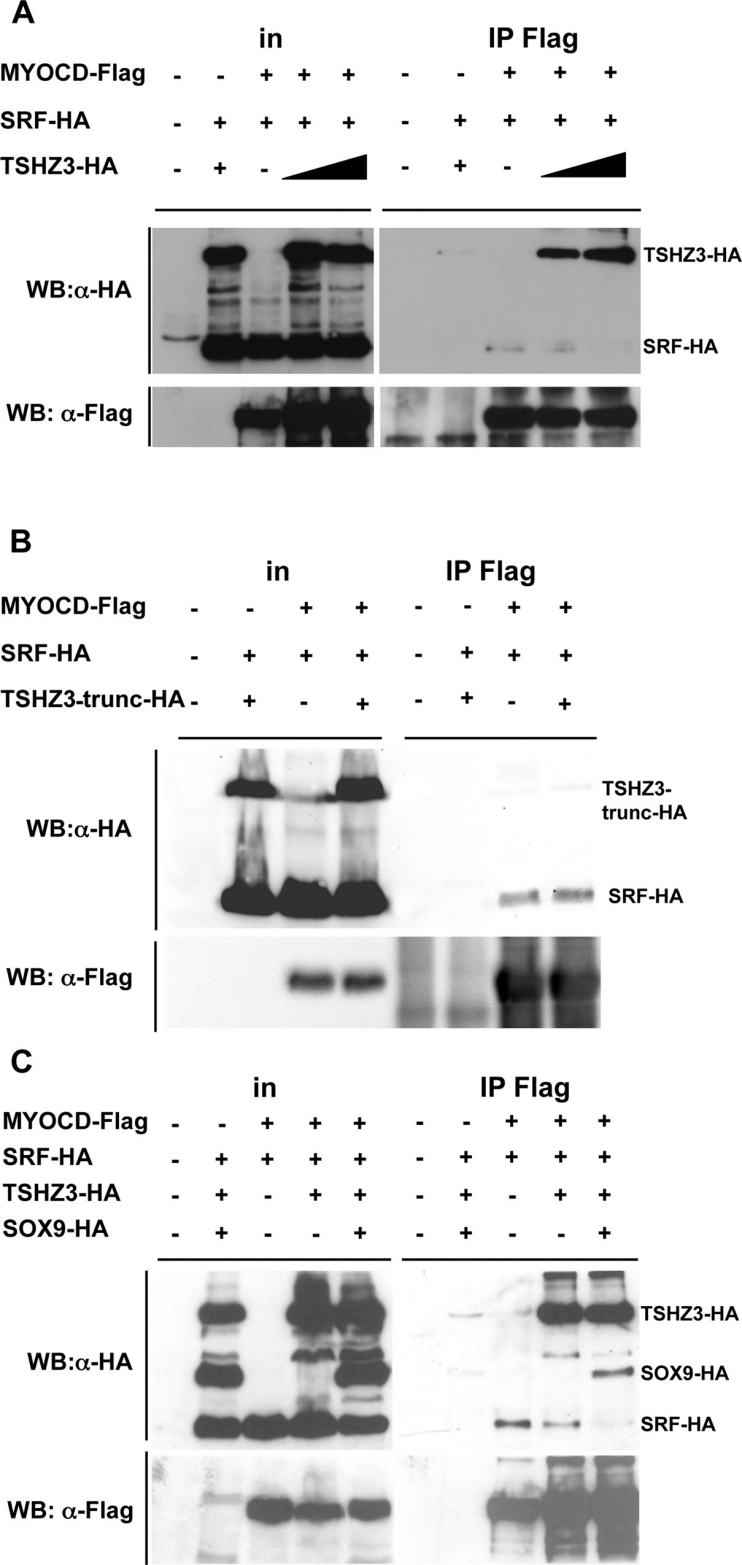
TSHZ3 and SOX9 compete with SRF for binding to MYOCD. (A-C) HEK293T cells were transfected with vectors encoding HA-SRF, HA-SOX9, HA-TSHZ3, HA-TSHZ3-trunc with Flag-MYOCD or control empty plasmid as indicated. HA-tagged proteins were identified by their molecular weight. Flag-MYOCD was immunoprecipitated (IP) from nuclear extracts of the transfected cells and co-precipitating HA-tagged proteins were detected by Western blotting.

The authors provided the following explanations as to how the original figures were prepared; updated figures are included with this notice, and the original raw Western blot data underlying most of the affected figure panels are included in S1 File.

- Fig 1B: The original α-HA panel was comprised of input data (lanes 1–3) from a short exposure and IP Flag data (lanes 4–6) from a longer exposure. In addition, an empty lane was removed from the α-Flag Western blot panel. The new Fig 1B uses only the long exposure of the α-HA blot, and the empty lane (lane 4) has not been removed in either panel.
- Fig 1D: In the original blot, the experimental samples were separated by empty lanes. In generating the published figure, the empty lanes were removed and experimental samples were spliced next to one another. The new figure presents the original image with the empty spacer lanes intact.
- Fig 1F: To generate the published figure, the authors removed empty lanes from the IP Flag lanes of the α-HA blot. For the α-Flag blot, the authors spliced together lanes to remove empty lanes and to combine data from different exposures. The images were cropped so as not to show unspecific bands in IP lanes. In the new figure, the authors present data from a replicate experiment. Raw images used to generate both the original figure and the revised figure are provided with this notice.
- Fig 1G: In the published figure, a blank lane was removed between the input and IP Flag lanes of both panels. Lanes 4–6 in the α-HA blot appear to have been inverted vertically in preparing the figure. The new figure presents the intact blot images, with the empty lanes in each blot (lane 4) intact.
- Fig 5A: In the published figure, lanes 3 and 4 were flipped horizontally. The new panel presents the same data in a direct representation of the original blot image. Note that the loading order in the new figure differs from that in the original version of the figure.
- Fig 5B: Concerns were raised regarding an irregularity in lane 6 of the α-HA blot, and potential splicing between lanes 1 and 2 of the α-Flag blot. An empty lane was also excised between the input and IP Flag lanes of the α-Flag blot in the published figure. The original α-HA blot is no longer available, and so the authors provide data from a replicate experiment in the new figure.
- Fig 5C: In the published figure, empty lanes were removed from each panel between lanes 3 and 4. The new figure represents the original blot with the empty lane intact in each panel.
- Fig 5D: In the published figure, an empty lane was removed between lanes 3 and 4. The new figure represents the original blot with the empty lane intact.
- Fig 6A: The published α-HA panel is comprised of lanes from a short exposure (input lanes) and long exposure (IP Flag lanes) of the same experiment. The IP Flag lanes were inverted horizontally, and empty lanes between experimental samples were removed. The new figure presents the intact image from the long exposure, with empty lanes included. Note that the loading order for the new panel differs from the loading order of the α-Flag blot shown in Fig 6A of the original publication.
- Figure S4A: The raw data image provided for the input lanes of the α-Flag blot panel (S2 File) does not match the image in the published figure. Raw data supporting the α-HA blot in this figure panel are in S3 File.

Panels F and G in the original version of Fig 5 [1] were mislabeled and should have been labeled panels E and F. The corresponding figure legend in [1] likewise contained an error in referencing these panels. These errors have been corrected in the updated version of Fig 5 provided here; the corrected labels (E, F) align with the in-text citation to these figure panels in the Results section of [1].

Raw data supporting other results reported in the article are provided in S4–S20 Files.

The underlying raw data are no longer available for the α-Flag blot in Fig 5D, or for Figs 1H/I, 2D/E/F, 4, 5G, 6B, 6C, 7C, S1, S2Q/R, S3, S4B/E, or S5. For Figure S5 in [1], the authors provided an updated figure that reports replication data. The new version of this figure is included with this notice as S21 File, and the raw data from the replication experiments are in S22–S25 Files.

Given the unavailability of these data and the extent of concerns with the images in the published article [1], the *PLOS ONE* Editors issue this Expression of Concern.

## Supporting information

S1 File Raw blots and author comments regarding Figs 1, 5, and 6. (PDF)

S2 File Original blot underlying the IP HA lanes of the α-Flag blot shown in Figure S4A. (TIF)

S3 File Original blot underlying the α-HA blot shown in Figure S4A. (TIF)

S4 File Original blots underlying the α-HA and α-Flag blots shown in Figure S4D. (PDF)

S5 File Original data supporting Fig 1E, upper panel. (ZIP)

S6 File Original data supporting Fig 1E, lower panel. (ZIP)

S7 File Original data supporting Figure 2A-C. (TIF)

S8 File Original data supporting Figure 2G-I. (TIF)

S9 File Original data supporting Figure 3A-C. (TIF)

S10 File Original data stack supporting Figure 3D. (TIF)

S11 File Original data stack supporting Figure 3E-G. (TIF)

S12 File Original data supporting Figure 3H. (TIF)

S13 File Original data supporting Figure S2A. Panel B in this figure shows a higher magnification view of this same image.
(TIF)

S14 File Original data supporting Figure S2C and S2D. (TIF)

S15 File Original data stack supporting Figure S2E-G. (TIF)

S16 File Original data stack supporting Figure S2HIJ. (TIF)

S17 File Original data stack supporting Figure S2K-M. (TIF)

S18 File Original data stack supporting Figure S2N-P. (TIF)

S19 File Original data supporting Figure S4C, upper panel. (ZIP)

S20 File Original data supporting Figure S4C, lower panel. (ZIP)

S21 File Figure S5. Expression of *Sox9* is not affected in *Tshz3* mutant ureters. (A, B) *Sox9* expression in wild type ureter. (C, D) *Sox9* expression in *Tshz3* mutant ureter.
(TIF)

S22 File Replication data presented in updated Figure S5A. (TIF)

S23 File Replication data presented in updated Figure S5B. (TIF)

S24 File Replication data presented in updated Figure S5C. (TIF)

S25 File Replication data presented in updated Figure S5D. (TIF)
