# Supplementary figures and images for: TSHZ3 and SOX9 Regulate the Timing of Smooth Muscle Cell Differentiation in the Ureter by Reducing Myocardin Activity
Source: PLoS One. 2013 May 6;8(5):e63721. doi: 10.1371/journal.pone.0063721 (PMC3646048; doi:10.1371/journal.pone.0063721)

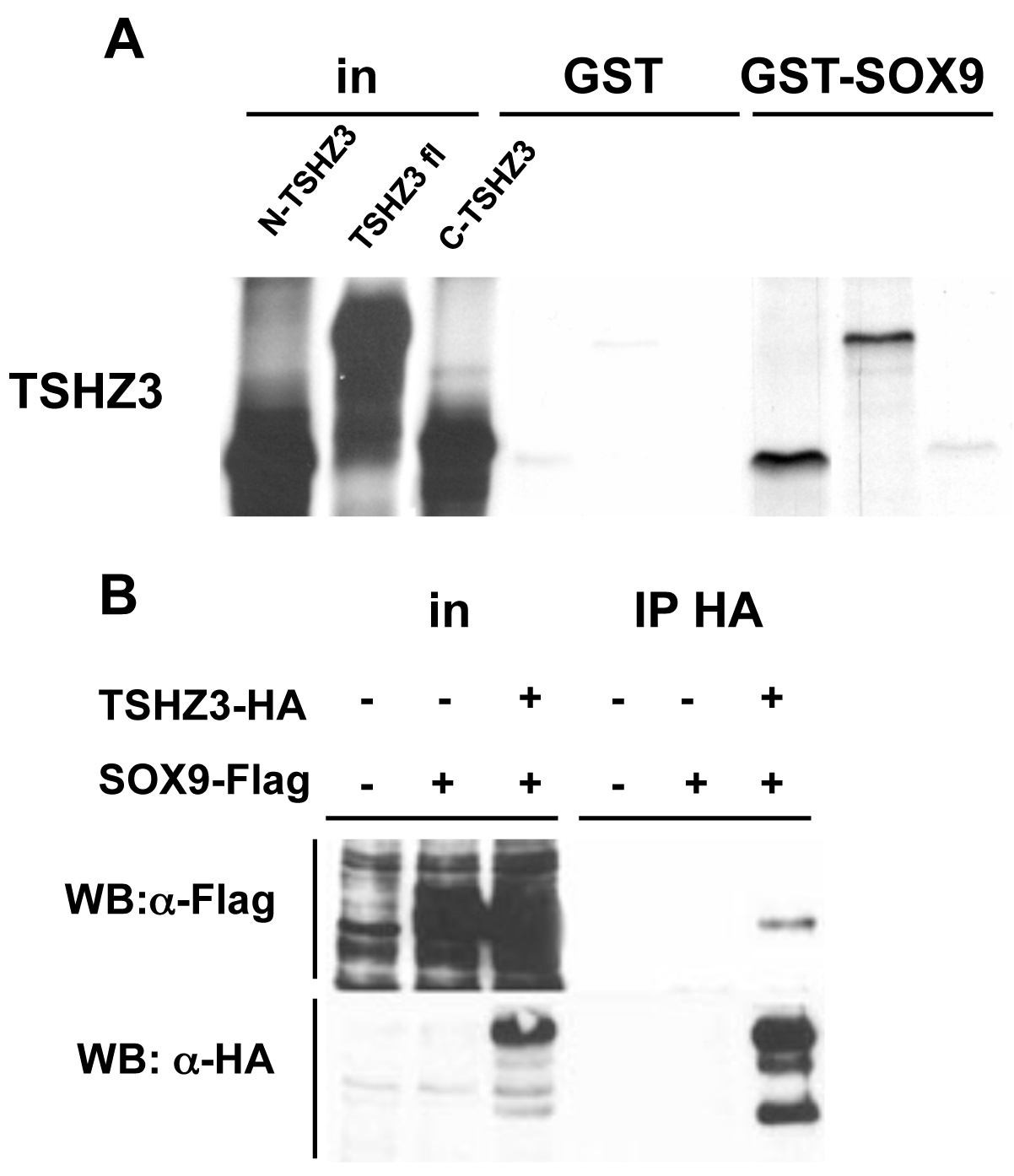

Supplement: Figure S1 — TSHZ3 and SOX9 form a complex in vivo and in vitro . (A) GST-SOX9 or GST alone were incubated with N-TSHZ3, TSHZ3fl, or C-TSHZ3 in vitro expressed in reticulocyte. (B) HEK cells were transfected with Flag-tagged SOX9 and HA-tagged TSHZ3. TSHZ3 proteins were immunoprecipitated using anti-HA antibody, as indicated. Immunoprecipitated proteins were identified by Western blotting using antibodies against the HA and Flag epitopes. (TIF) [file pone.0063721.s001.tif]

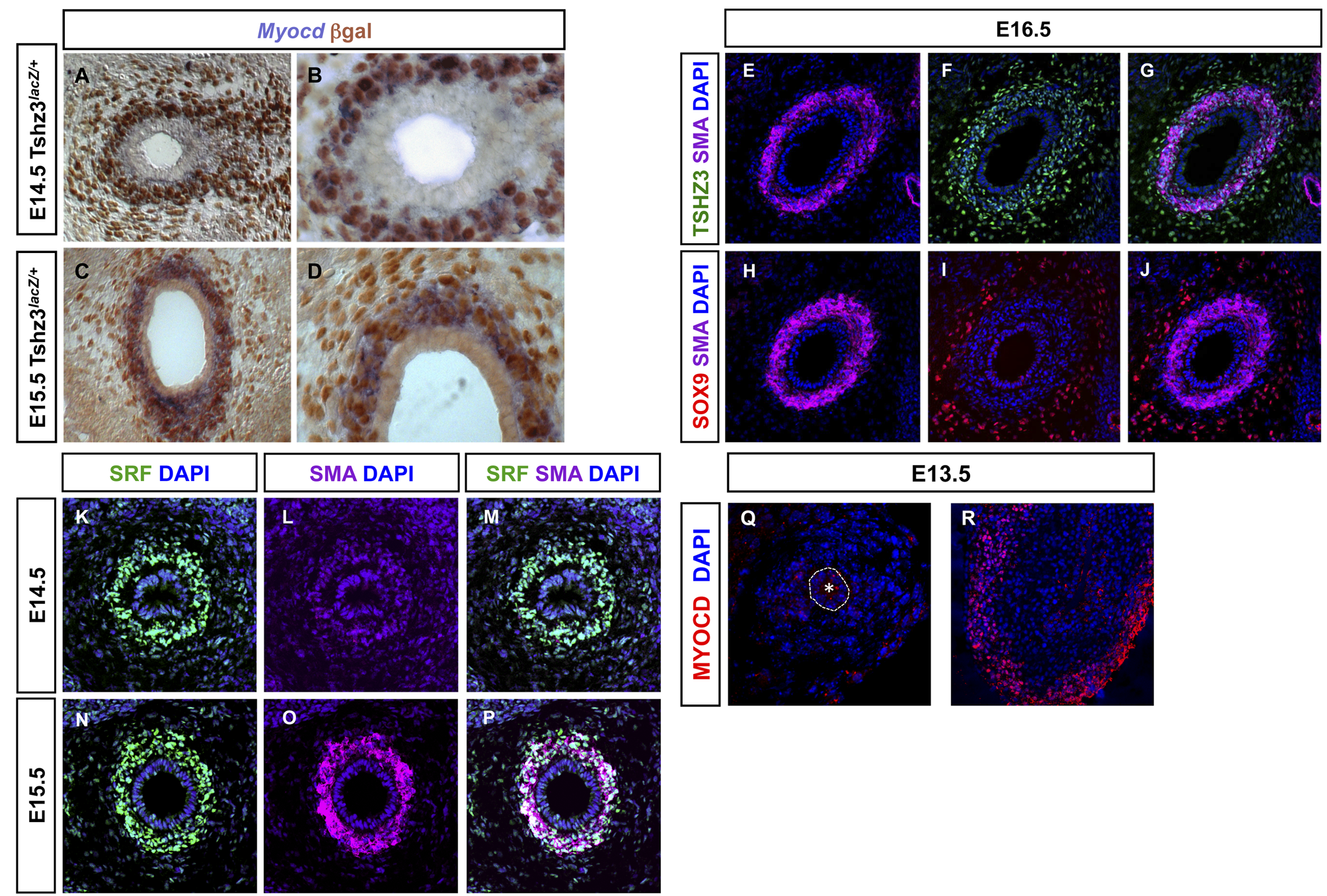

Supplement: Figure S2 — Maintenance of TSHZ3 protein in ureteric SM progenitors and SM cells, and loss of SOX9 detection in SM cells at E16.5. (A–D) Transverse sections of proximal ureters showing β-galactosidase immunostaining (brown), which recapitulates TSHZ3 expression, and expression of myocardin by in situ hybridization (purple signal) at E14.5 and E15.5. (E–J) Detection of THSZ3 protein (green, F, G) contrasting with absence of SOX9 (red, I, J) in SM cells stained with the SM marker: SMA (magenta, E, H) on transverse sections of ureters at E16.5. (K–P) Detection of SRF (green, K, M, N, P) and SMA (magenta, L, M, O, P) on transverse sections of ureters at E14.5 (K–M) and E15.5 (N–P). (Q, R) Expression of Myocardin in the ureter and the bladder at E13.5. Q and R, Immunostaining for Myocardin (red) and nuclei (DAPI, Blue). Myocd was not detected in the ureteral mesenchyme (A), in contrast to the bladder (B). Dotted line in A separates the epithelial layer and the mesenchymal layer. Asterisk indicates the lumen of the ureter. (TIF) [file pone.0063721.s002.tif]

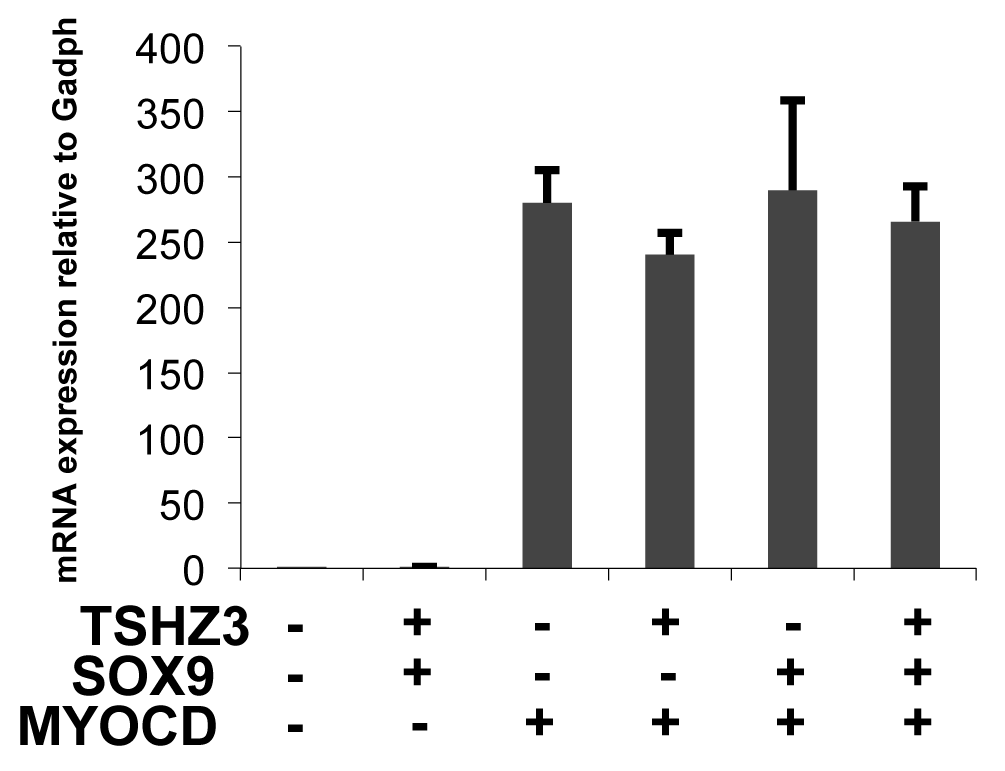

Supplement: Figure S3 — Myocd expression is stable. 10T1/2 cells were cotransfected with the indicated constructs. 24 hours post nucleofection, total RNA was harvested from cells and expression levels of exogenous Myocd were measured by qRT-PCR. (TIF) [file pone.0063721.s003.tif]

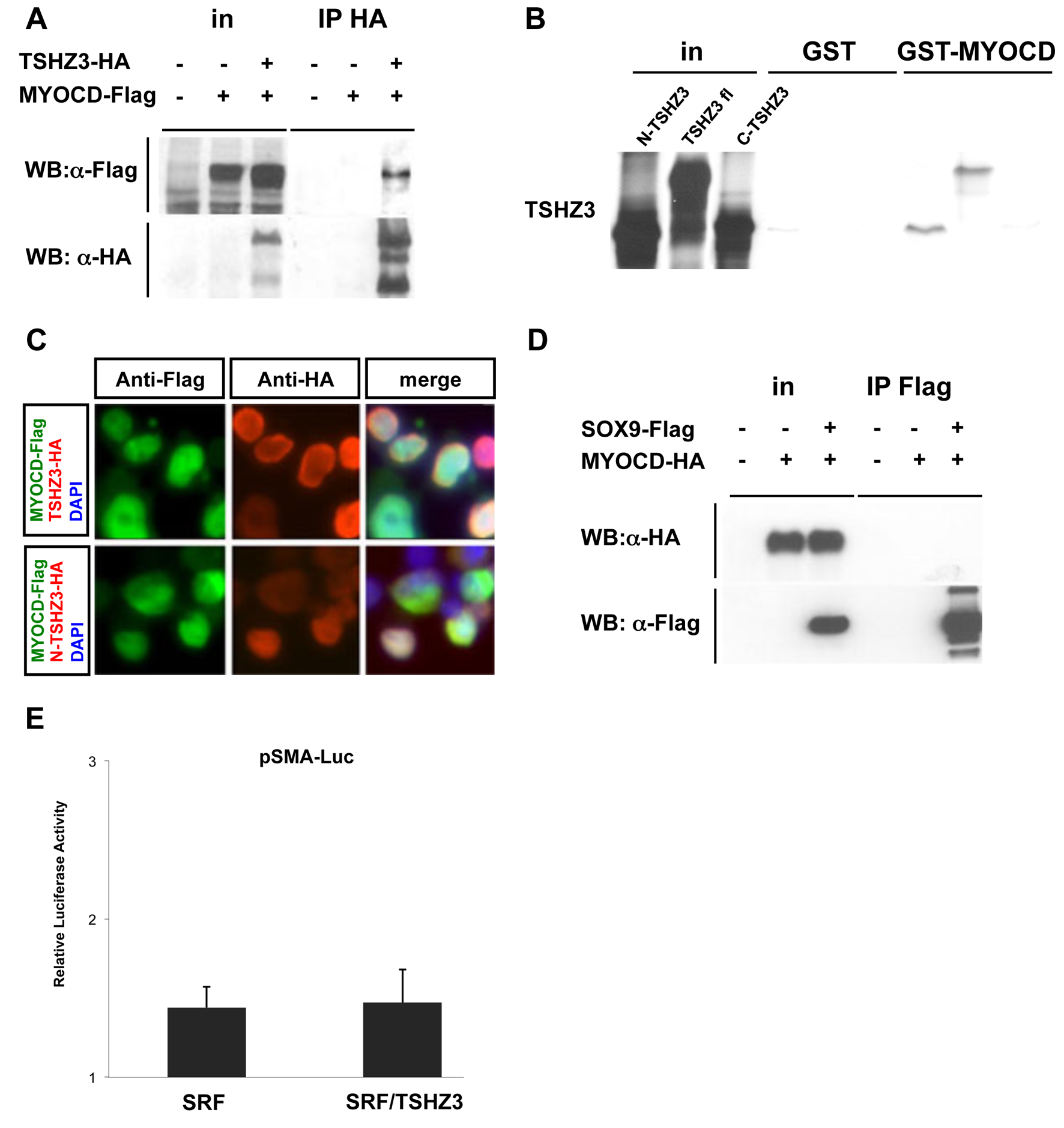

Supplement: Figure S4 — TSHZ3 interacts with MYOCD. (A) HEK cells were transfected with expression vectors encoding Flag-tagged SOX9 and HA-tagged MYOCD. Cell lysates were incubated with anti-Flag (SOX9) antibody, and the immunoprecipitates were probed with anti-HA (MYOCD) or anti-Flag (SOX9) antibodies by Western analysis. (B) Aliquots of in vitro translated 35S-labelled full-length (TSHZ3 fl) and deletion mutants of TSHZ3 (N-TSHZ3, C-TSHZ3) were incubated with glutathionne-Sepharose beads loaded with bacterially expressed GST alone or GST-MYOCD. The retained 35S-labeled TSHZ3 proteins were separated on SDS-PAGE followed by autoradiography. 10% of input (in) is shown on the left. In control experiments, no binding of TSHZ3 proteins to the GST beads was observed. (C) TSHZ3-HA (red, anti-HA epitope tag), N-TSHZ3-HA (red) and MYOCD-Flag (green, anti-Flag epitope tag) localize to the nucleus in HEK293T transfected cells. Cells were counterstained with DAPI to detect nuclei. (D) HEK cells were transfected with expression vectors encoding Flag-tagged MYOCD and HA-tagged TSHZ3. Cell lysates were incubated with anti-HA (TSHZ3) antibody, and the immunoprecipitates were probed with anti-HA (TSHZ3) or anti-Flag (MYOCD) antibodies by Western analysis. (E) 10T1/2 with indicated combination of expression constructs and a luciferase reporter controlled by SMaA. Luciferase activity was measured. SRF significantly activated SMA luciferase promoter around 1,5 fold relative to the basal activity of SMA promoter alone. This activation was unaffected by co-transfection with TSHSZ3. An arbitrary value of 1 was assigned to the basal activity of SMA promoter alone. (n = 12, mean ± S.EM). (TIF) [file pone.0063721.s004.tif]

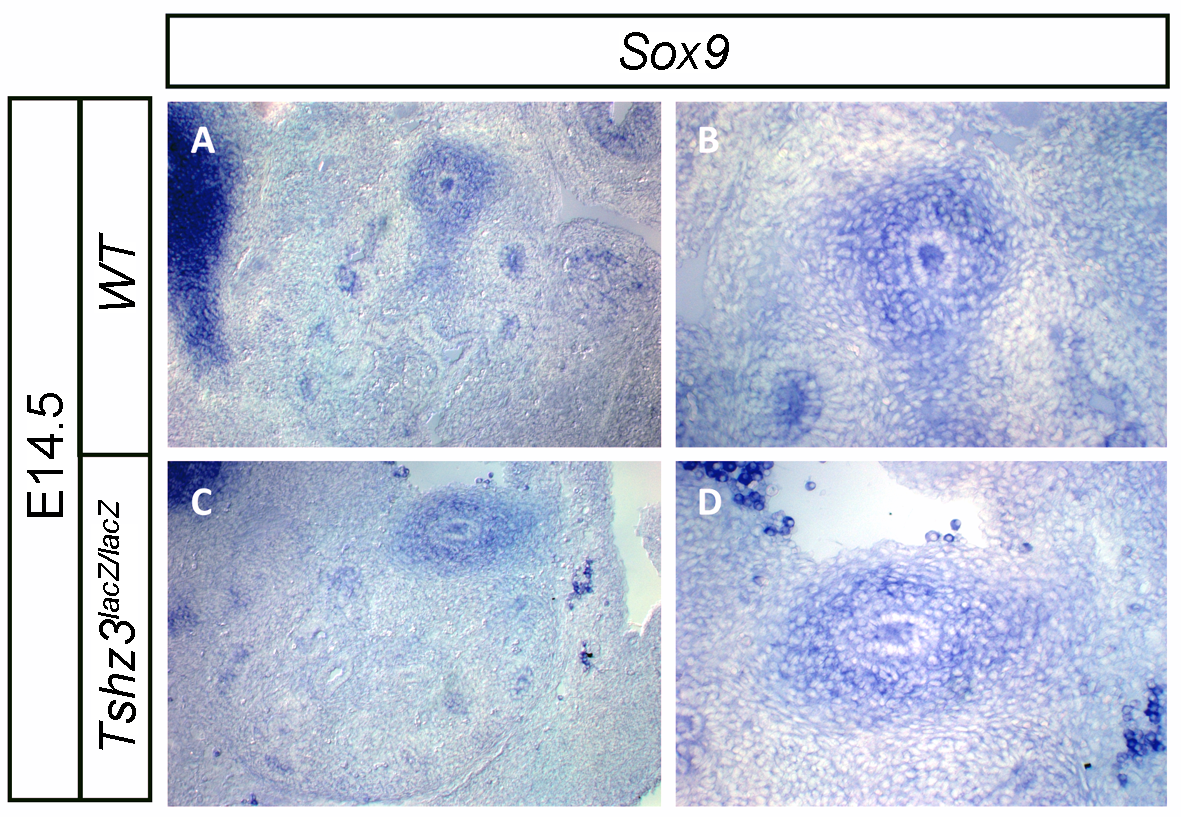

Supplement: Figure S5 — Expression of Sox9 is not affected in Tshz3 mutant ureters. (A, B) Sox9 expression in wild type ureter. (C, D) Sox9 expression in Tshz3 mutant ureter. (TIF) [file pone.0063721.s005.tif]
