# Supplementary material for: Expression of Concern: TSHZ3 and SOX9 Regulate the Timing of Smooth Muscle Cell Differentiation in the Ureter by Reducing Myocardin Activity
Source: PLoS One. 2019 Feb 11;14(2):e0211924. doi: 10.1371/journal.pone.0211924 (PMC6370214; doi:10.1371/journal.pone.0211924)
Supplement: S1 File — (PDF) [file pone.0211924.s001.pdf]

Published Figure 1B :

- **PlosOne concern**: In **Figure 1B**, the right two lanes in the top panel (WB: **alpha-HA**) appear to have been spliced in.

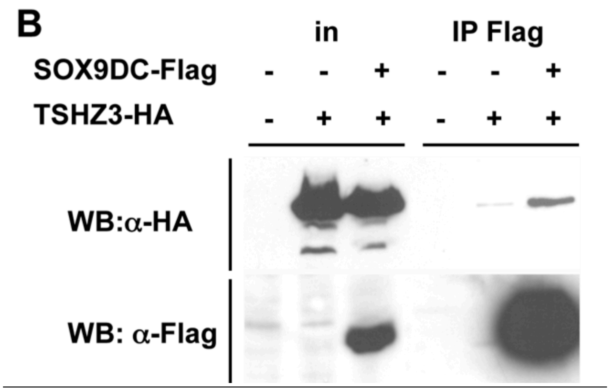

Proposition: The data used to build the original figure are framed in red. **For the Anti HA,** We combined a **long** exposure (left) and **a short** exposure (middle). Anti Flag (right).

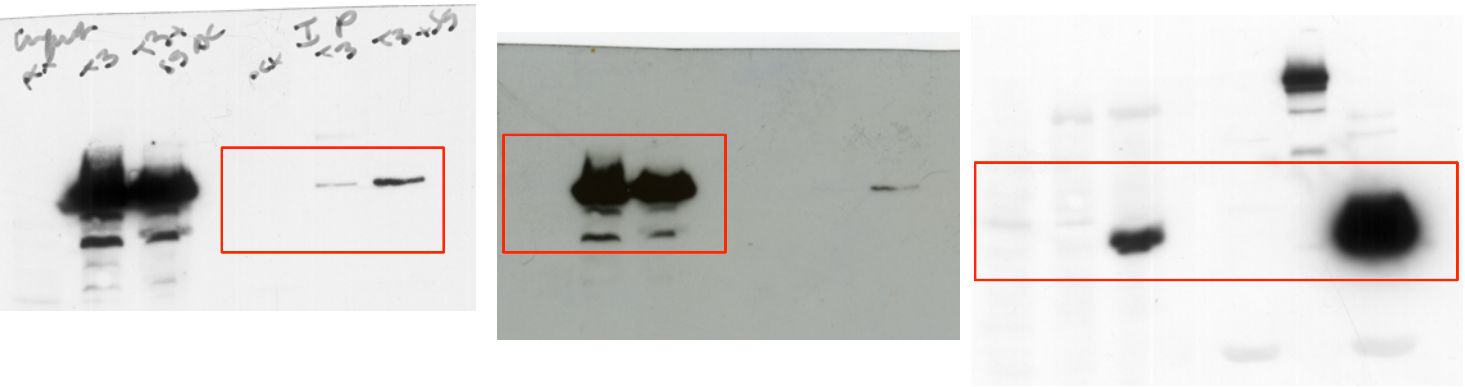

New Figure 1B using only the long exposure and without removing the empty lines:

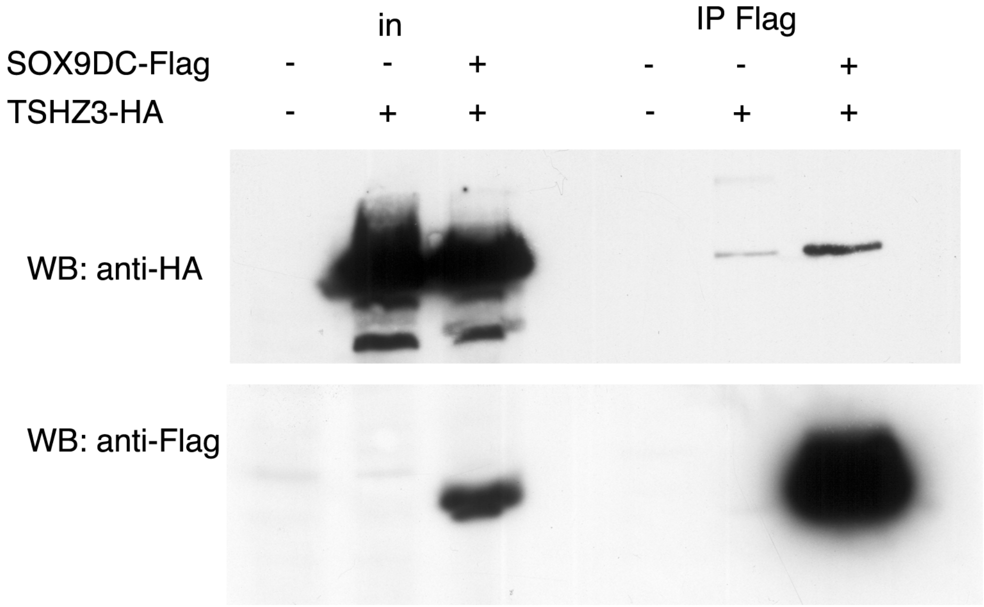

The Figure legend is unchanged

Published Figure 1D :

- **PlosOne concern**: In Figure 1D, the middle three lanes appear to have been spliced in. There appears to be a cut after the Input lane and after the GST-TSHZ3 fl lane.

D

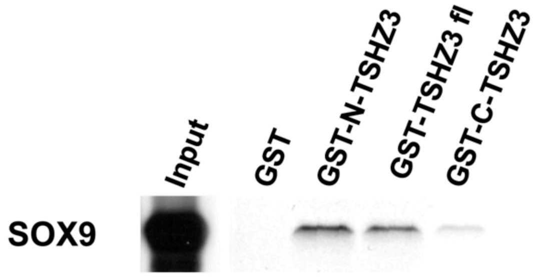

Proposition: **Original gel below with empty lanes.**

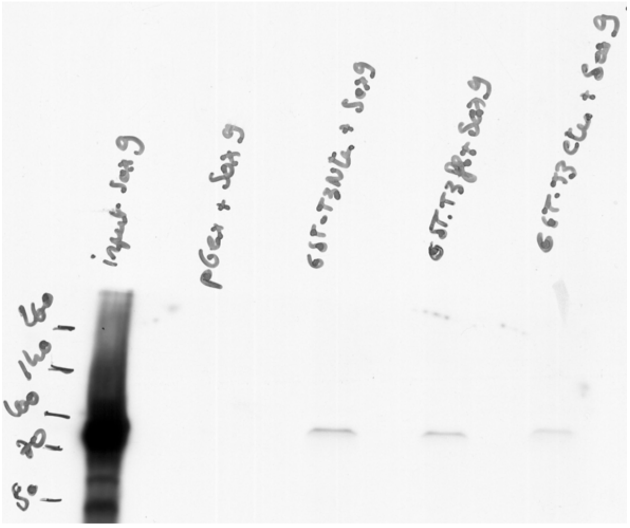

NEW Figure 1B without removing the empty lines:

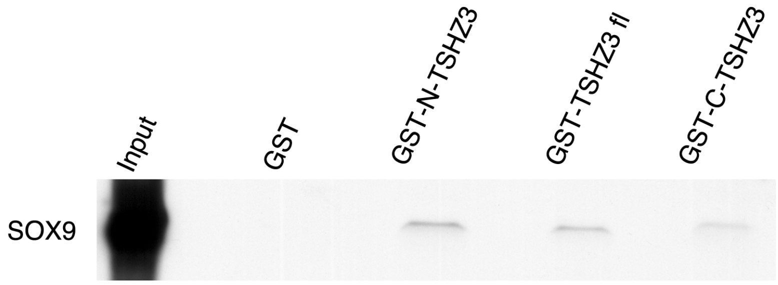

The Figure legend is unchanged

Published Figure 1F :

- **PlosOne concern**: In **Figure 1F** for WB:

1. **alpha-HA**, there appears to be a cut between lanes 1 and 2 and a cut between lanes 3 and 4. There also appear to be two modified patches on the right two lanes.
2. **alpha-Flag**, there appears to be a cut between lanes 4 and 5 and a cut between lanes 5 and 6. There also appear to be two modified patches: one in lane 3 and one between lanes 5 and 6.

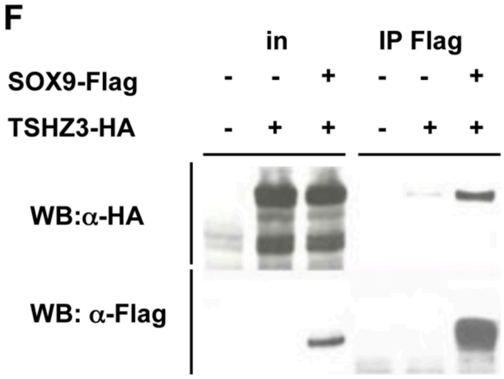

Proposition: To build a new figure with no cut, showing the same results, we propose to use the original gels below that come from another experiment.

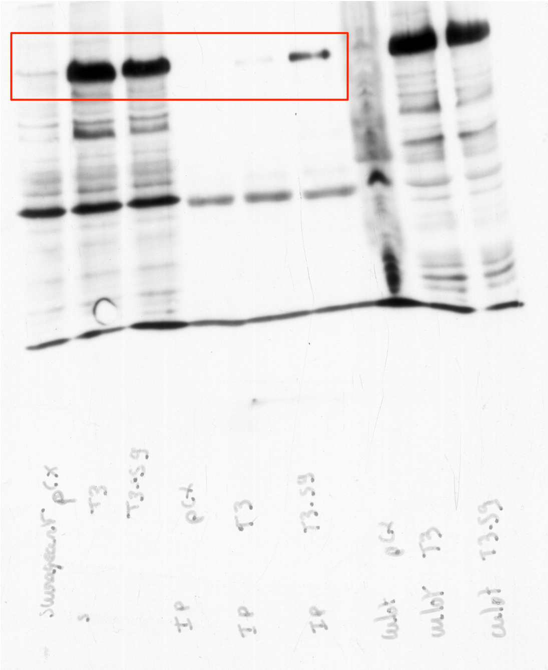

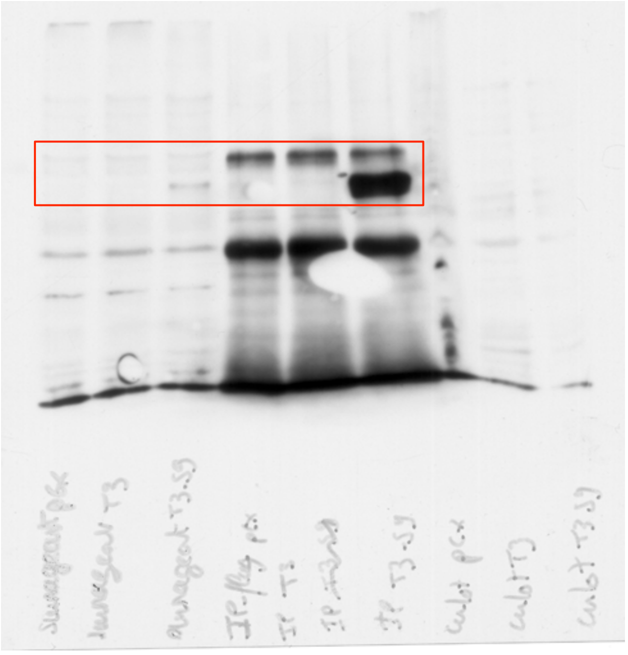

NEW Figure 1F without removing the empty lines:

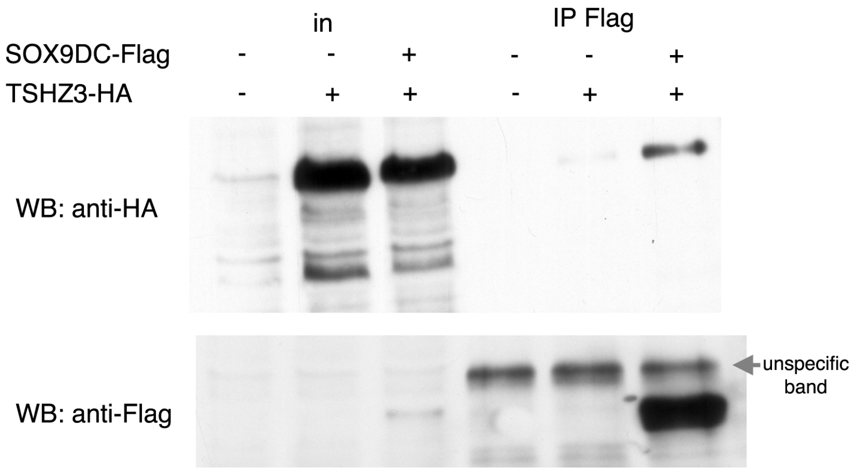

The Figure legend is unchanged

Published Figure 1G :

PlosOne concern:
alpha-HA, the first three lanes appear to have been spliced in. The band in the 6th lane also appears to have been spliced in.
alpha-Flag, there appears to be a modified patch in the top of lane 4.

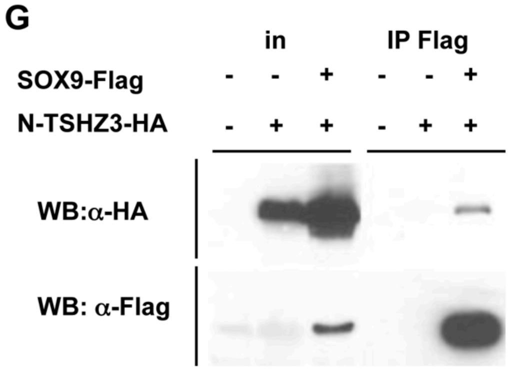

Proposition:

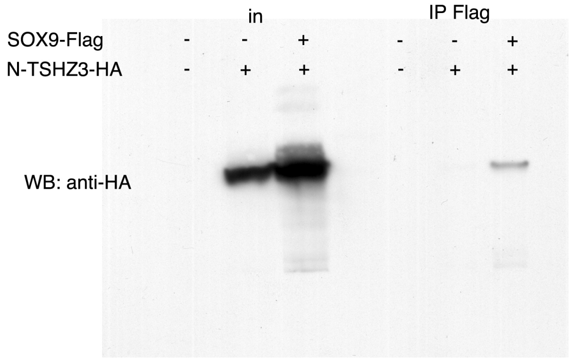

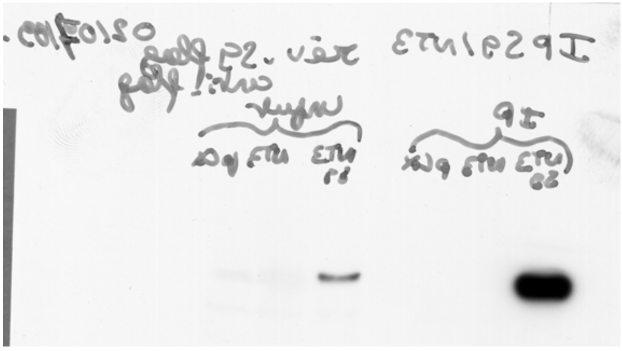

NEW Figure 1G without removing the empty lines:

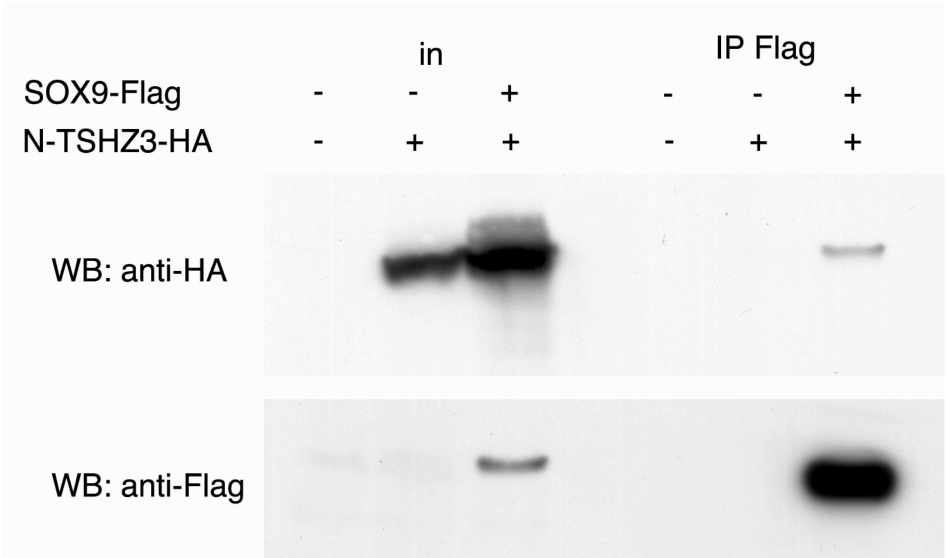

The Figure legend is unchanged

Published Figure 5A :

- **PlosOne concern**: In Figure 5A, there appears to be a modified patch in the second lane (GST). Lane 5 also appears to have been spliced in.

A

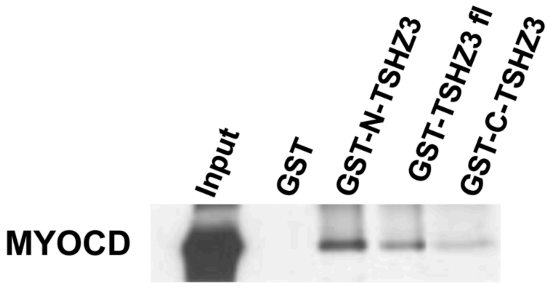

Proposition:

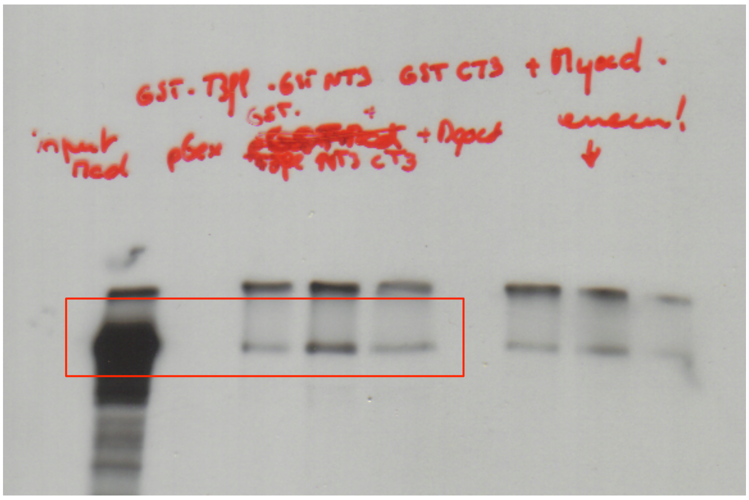

The original blot is shown here. For convenience, in the published figure we cut and flipped horizontally the lanes 3 and 4.
The data used to build the figure are framed in red.

NEW Figure 5A, showing the same result without any cut

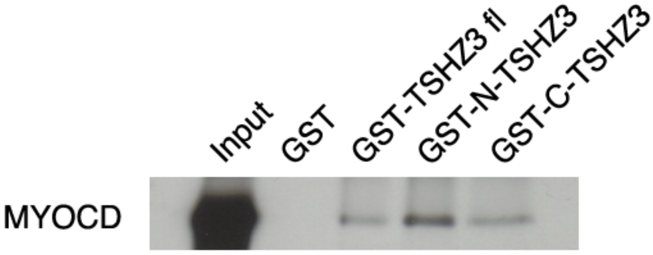

The Figure legend can remain unchanged

Published Figure 5B :

- **PlosOne concern**: In Figure 5B, for WB: **alpha-HA**, there appears to be a modified patch in lane 6**. alpha-Flag**, lane 1 appears to have been spliced in.

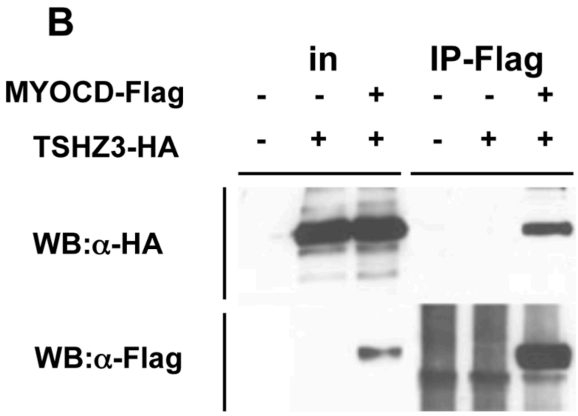

Proposition: The original gels below come from another experiment. The results are identical to the one we published; there is no cut. The data used to build the NEW figure are framed in red.

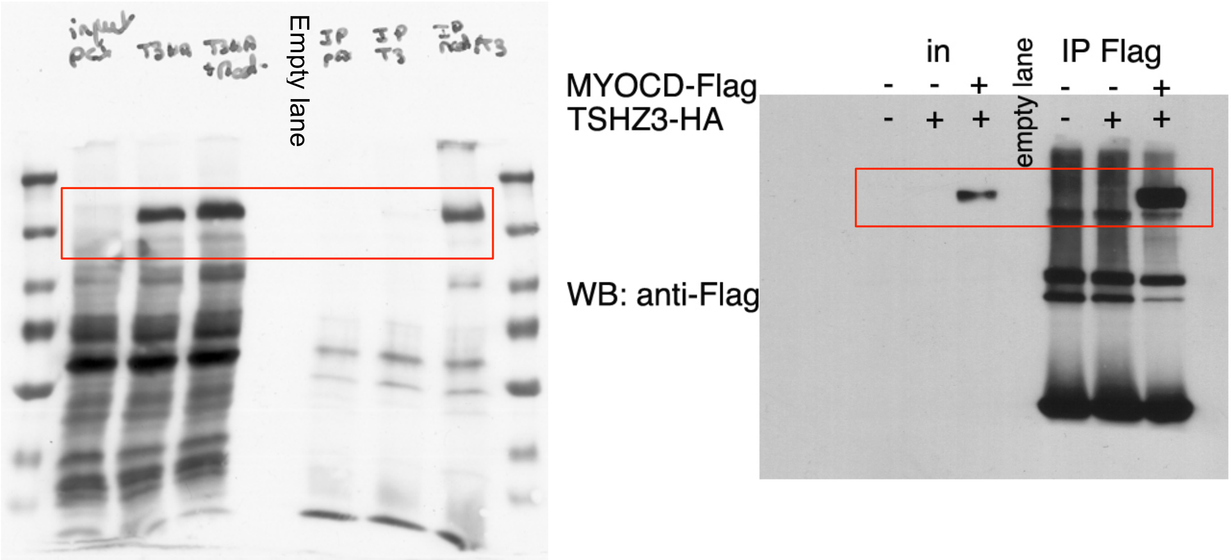

NEW Figure 5B, showing the results without any cut

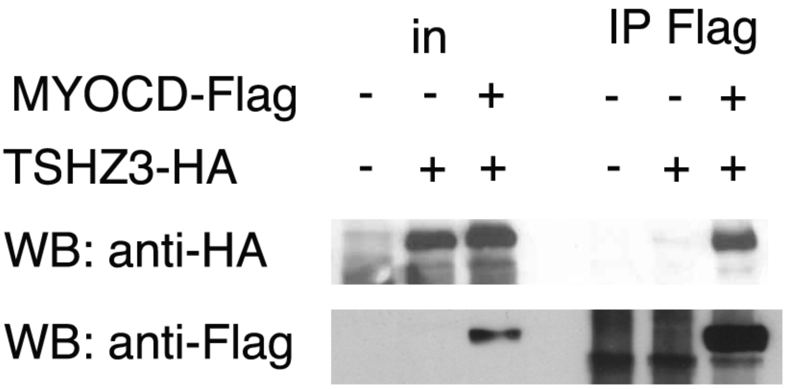

The Figure legend is unchanged

Published Figure 5C :

- **PlosOne concern**: In Figure 5C, there appears to be a cut in between lanes 3 and 4.

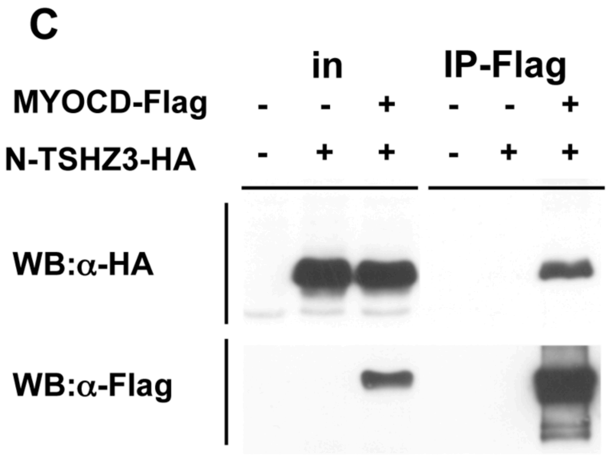

Proposition: **the original gels are shown below;** The data used to build the NEW figure are framed in red.

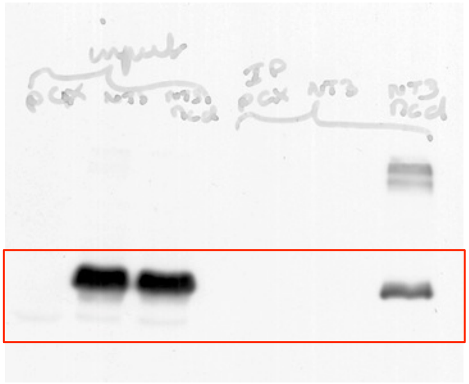

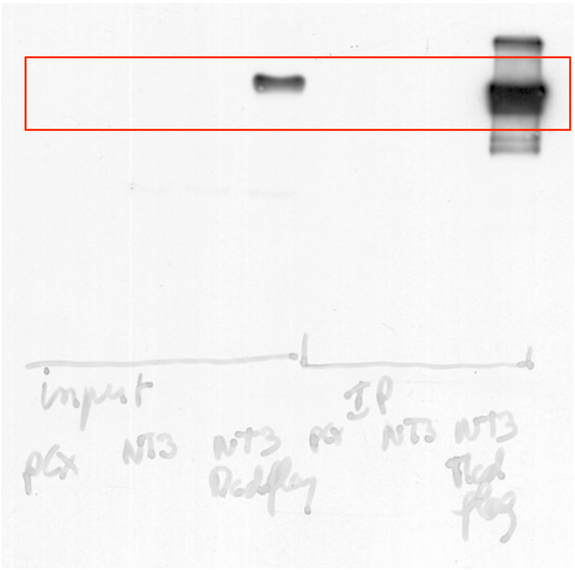

NEW Figure 5C, showing the results without any cut

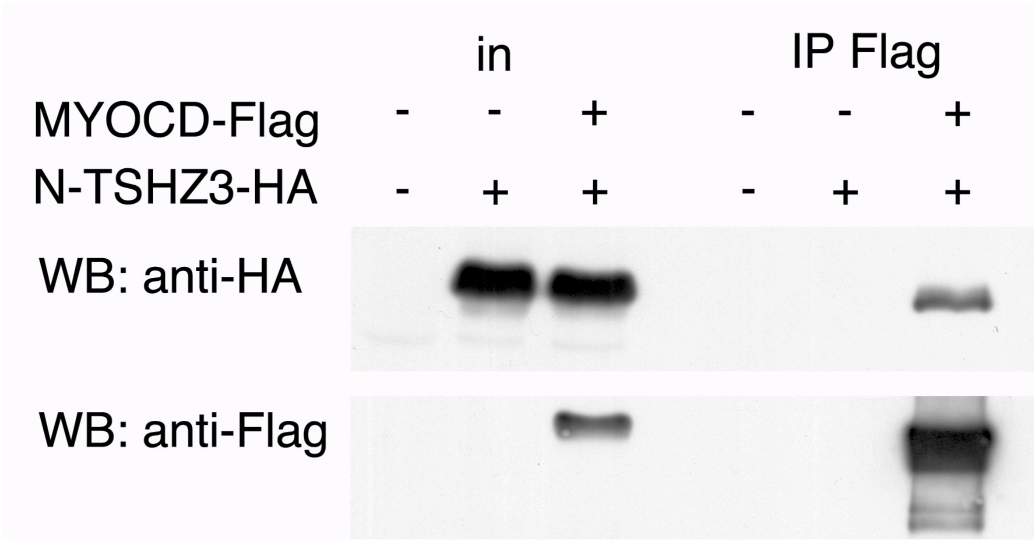

The Figure legend can remain unchanged

Published Figure 5D :

PlosOne concern: In Figure 5D, there appears to be a cut in between lanes 5 and 6 for WB: **alpha-HA**

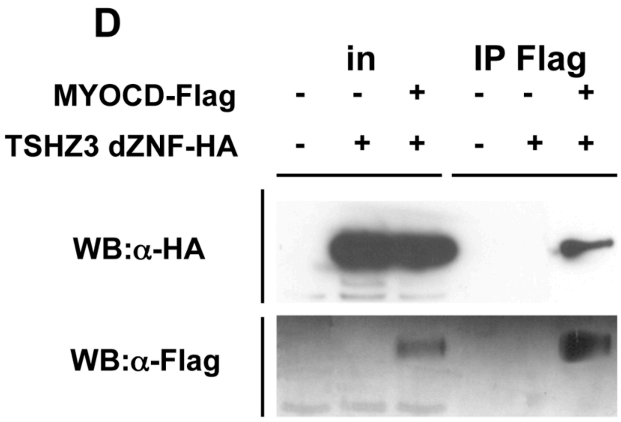

Proposition: The original gels are shown below. The data used to build the figure are framed in red.

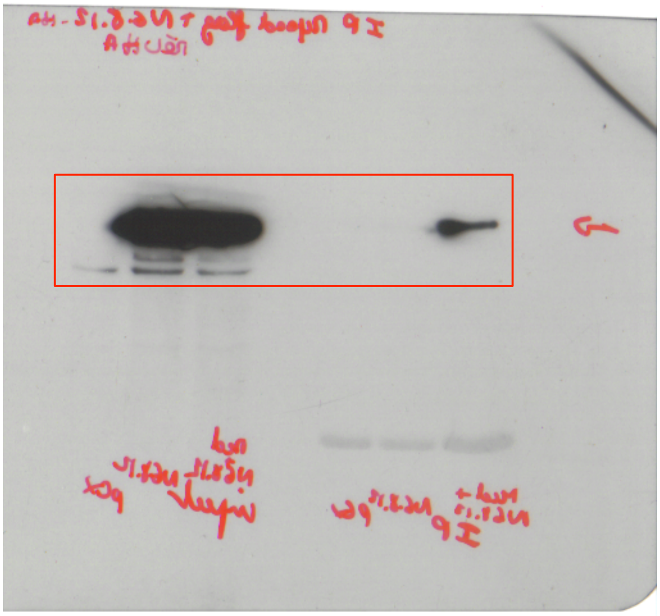

NEW Figure 5D α-HA

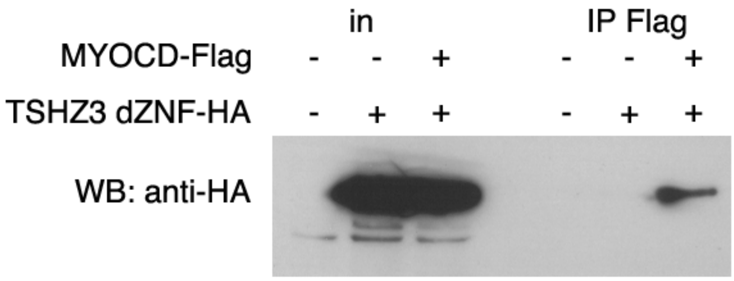

The Figure legend can remain unchanged

Published Figure 6A :

- **PlosOne concern**: In Figure 6A for WB: **alpha-HA**, there appears to be a cut between the 5th and 6th panels. There also appear to be several areas in this figure where the lanes were spliced in.

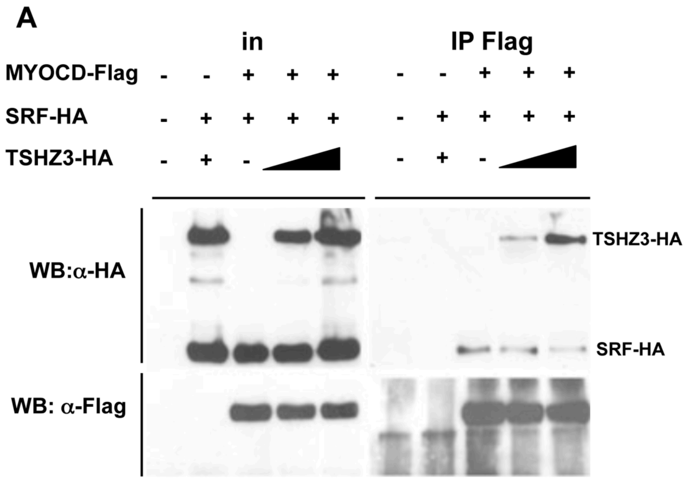

In the published figure, we combined a **short** (top) and **long** (bottom) exposure. The data used to build the WB: anti-HA are framed in red. The empty lanes that were cut to build the published Fig. 6A are visible. Unfortunately, the blot for the WB: anti-Flag has been lost.

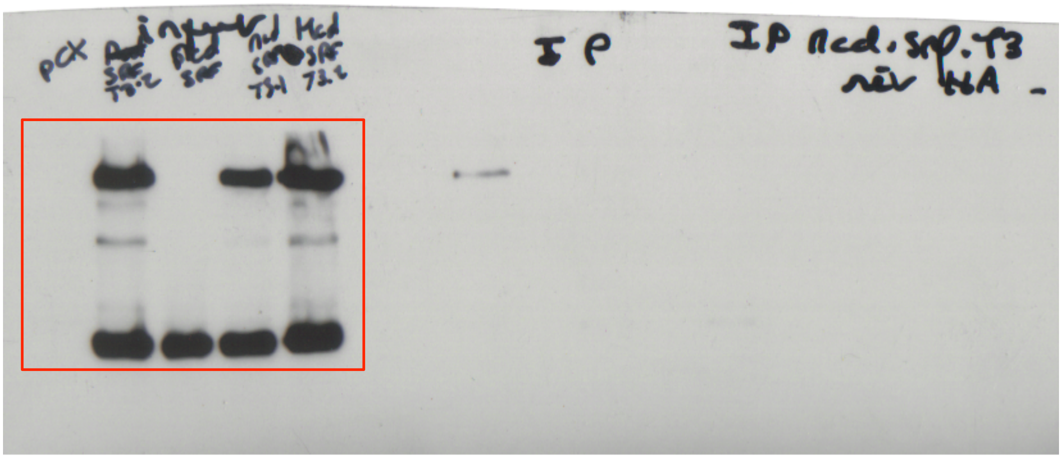

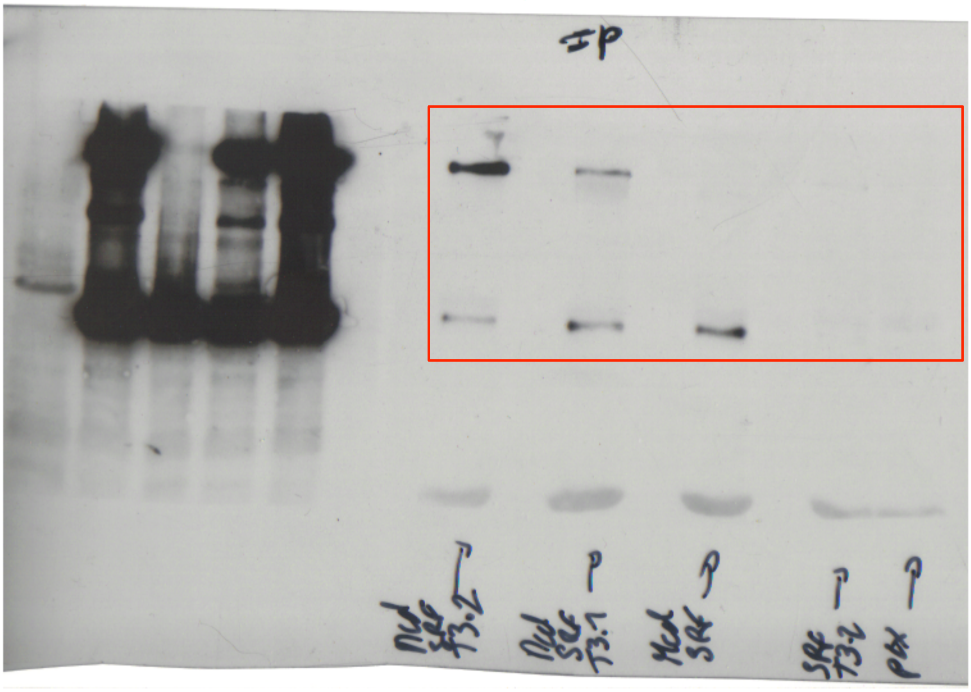

<u>Proposition:</u> We propose to build a new **Figure 6A** using the original blots shown below:

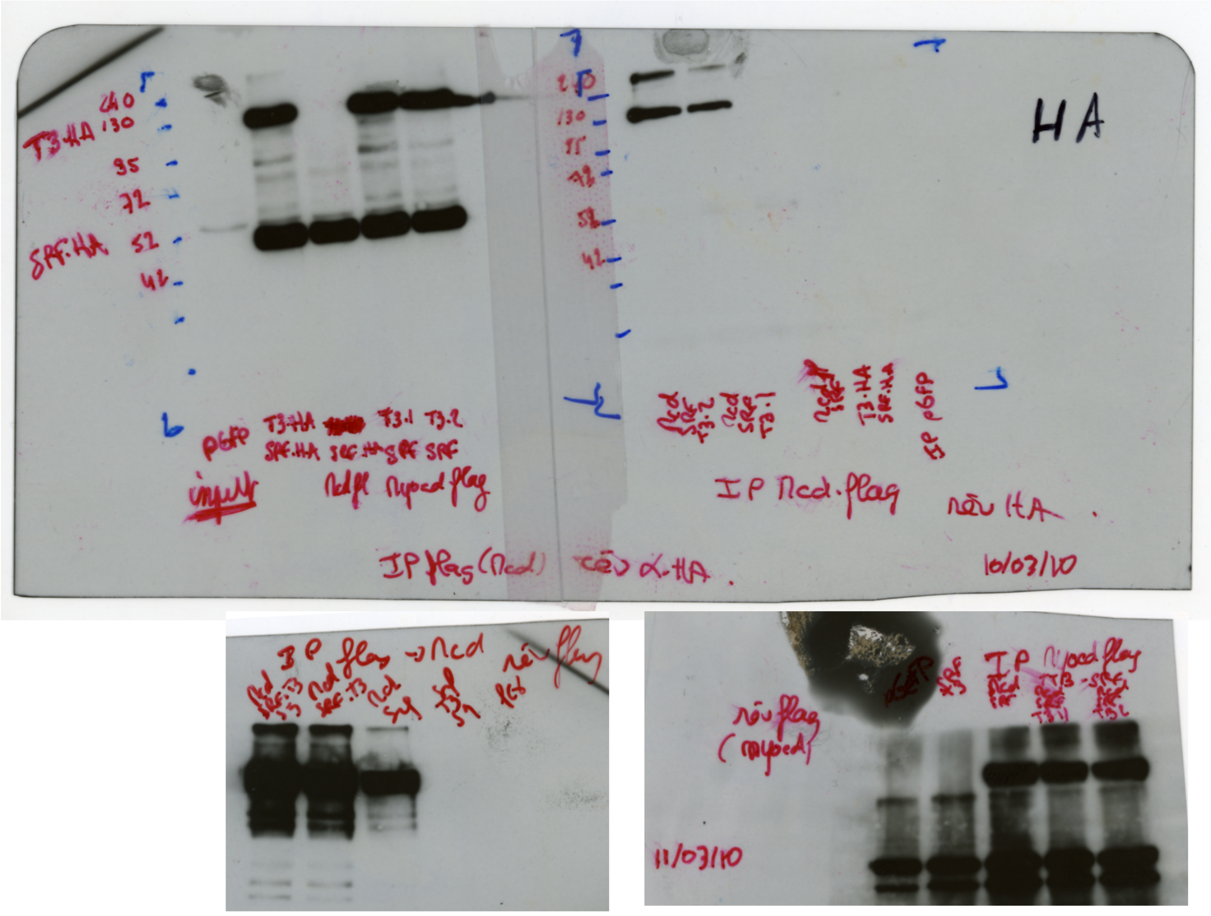

To build the figure, the top right and bottom left panels have to be flipped horizontally:

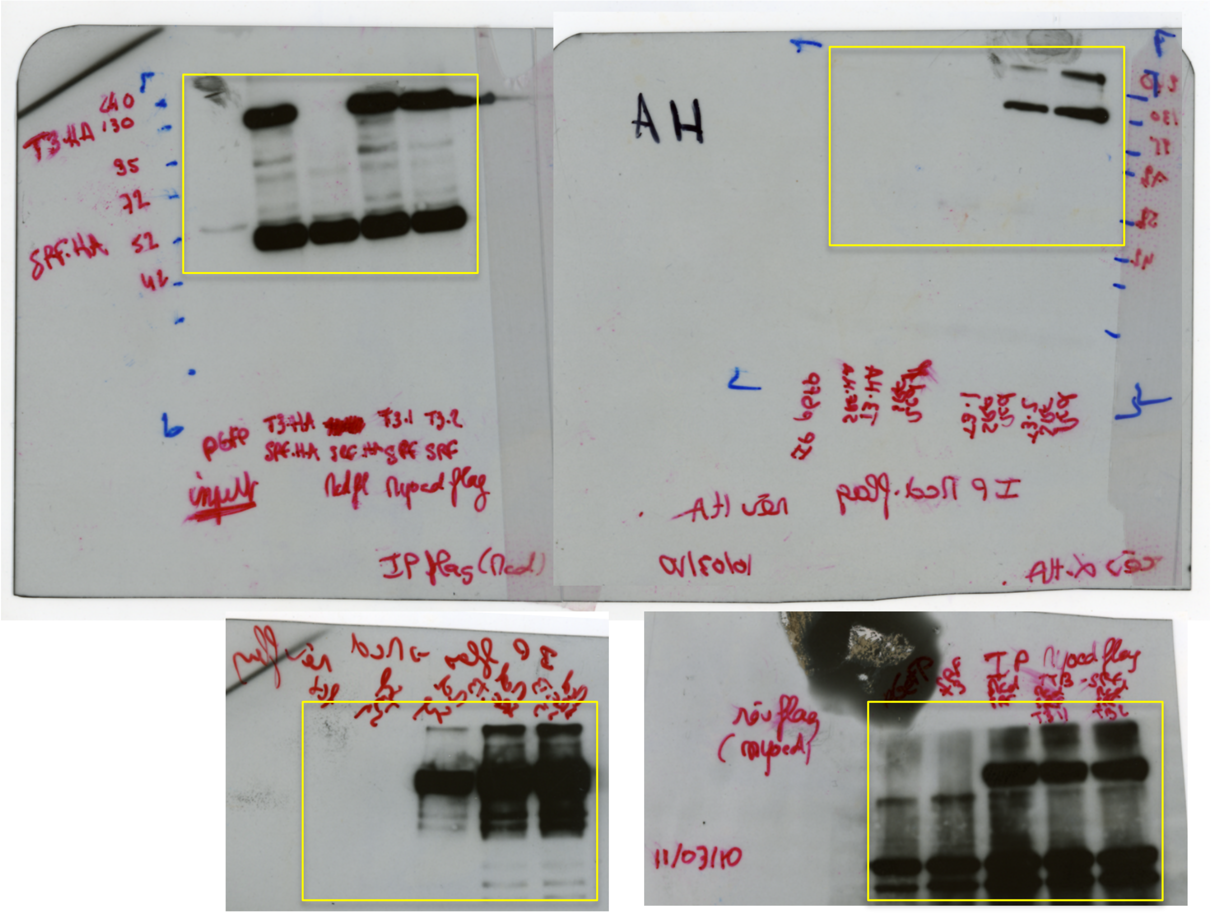

Last to build the new **Fig. 6A**, we use a longer exposure for the anti-HA blot:

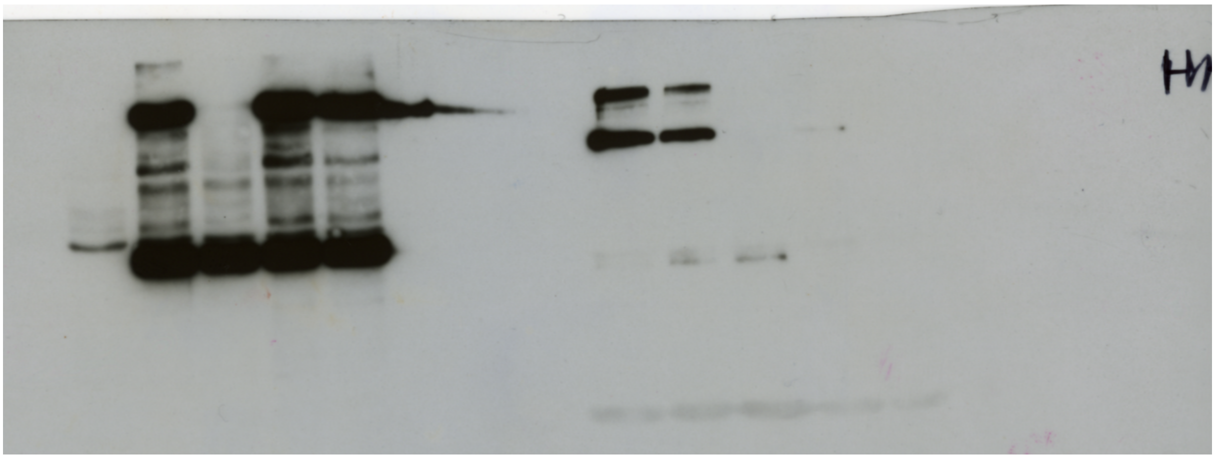

Below the proposed Figure 6A:

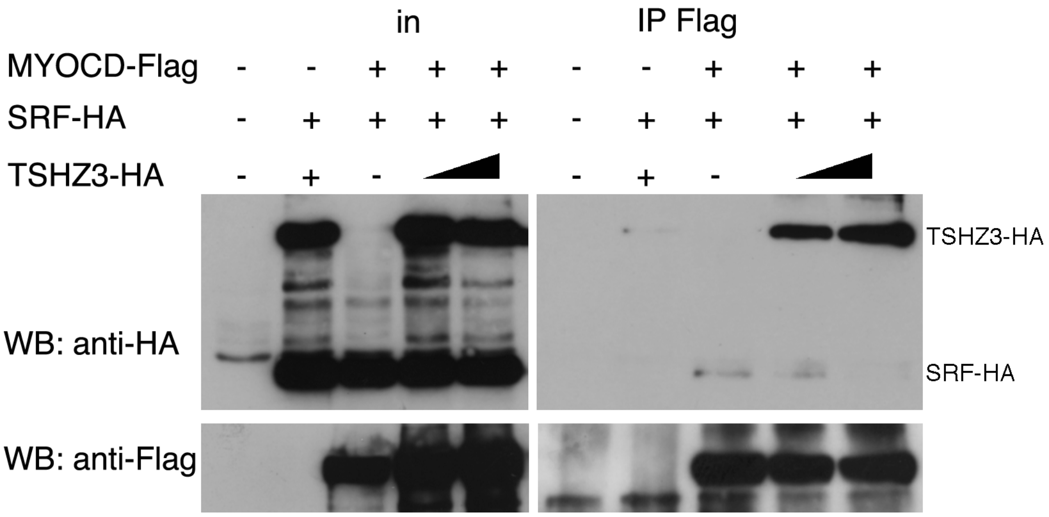

The Figure legend is unchanged
