# Supplementary figures and images for: Expression of Concern: TSHZ3 and SOX9 Regulate the Timing of Smooth Muscle Cell Differentiation in the Ureter by Reducing Myocardin Activity
Source: PLoS One. 2019 Feb 11;14(2):e0211924. doi: 10.1371/journal.pone.0211924 (PMC6370214; doi:10.1371/journal.pone.0211924)

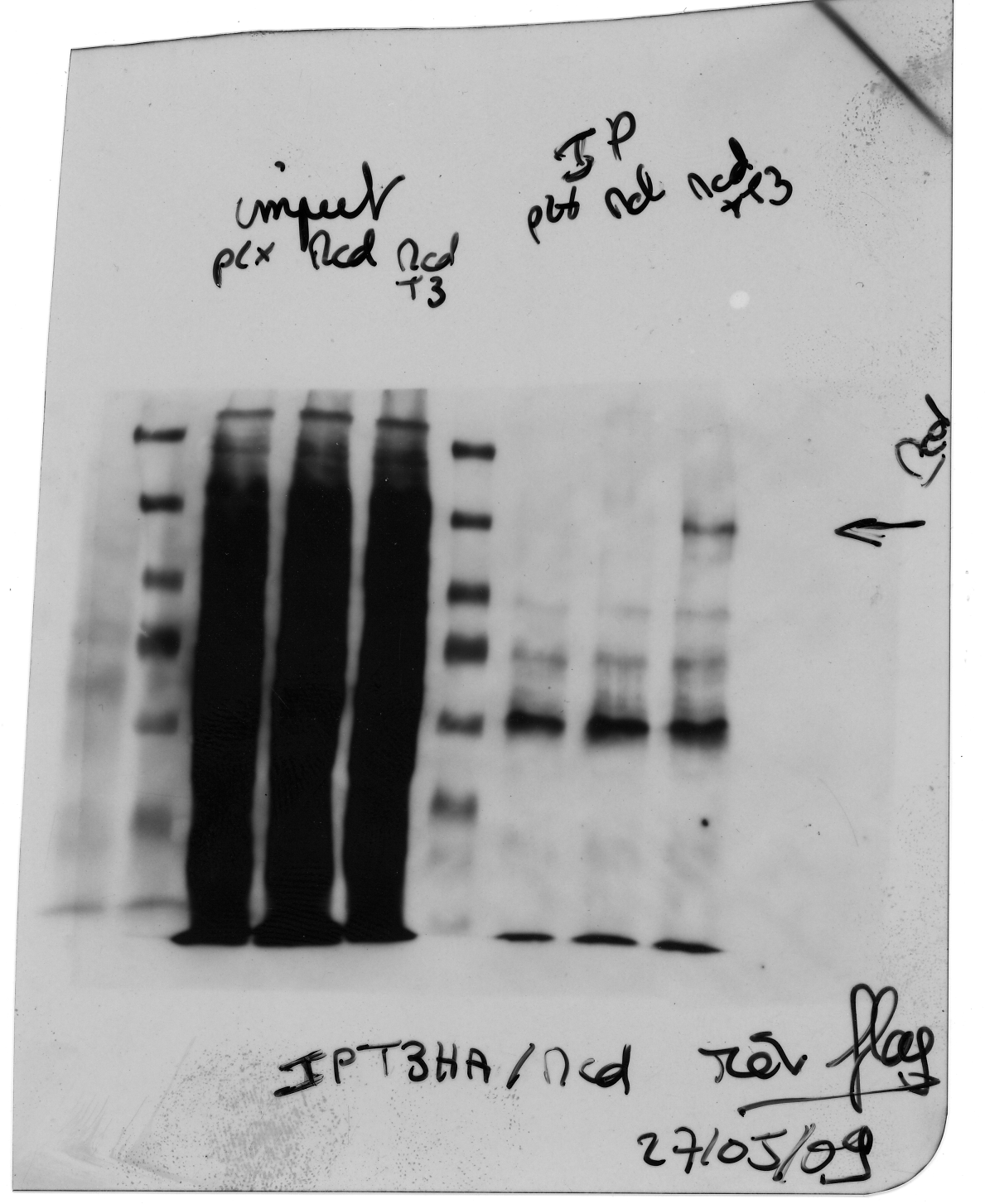

Supplement: S2 File — (TIF) [file pone.0211924.s002.tif]

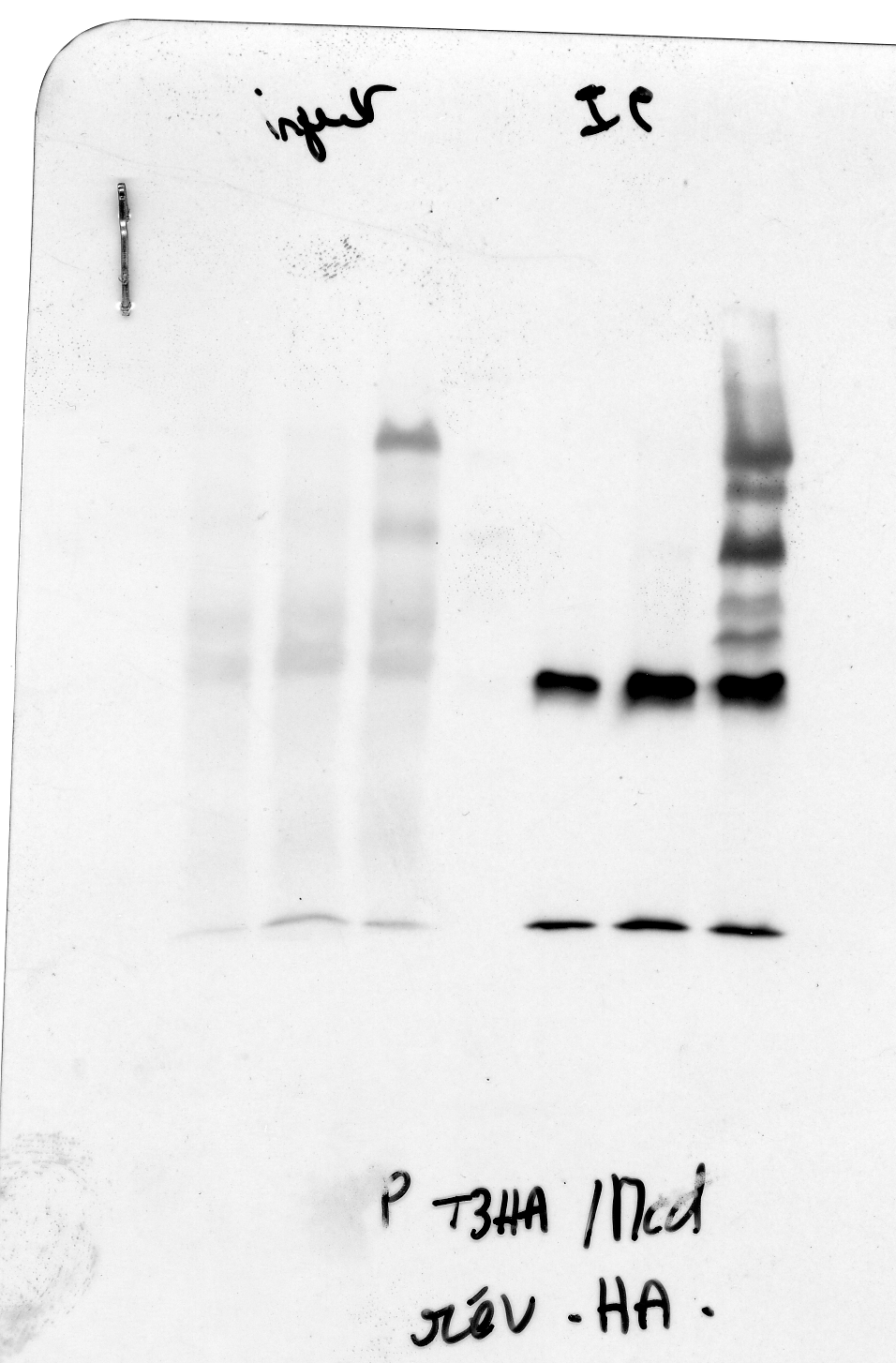

Supplement: S3 File — (TIF) [file pone.0211924.s003.tif]

Figure Sup 4D

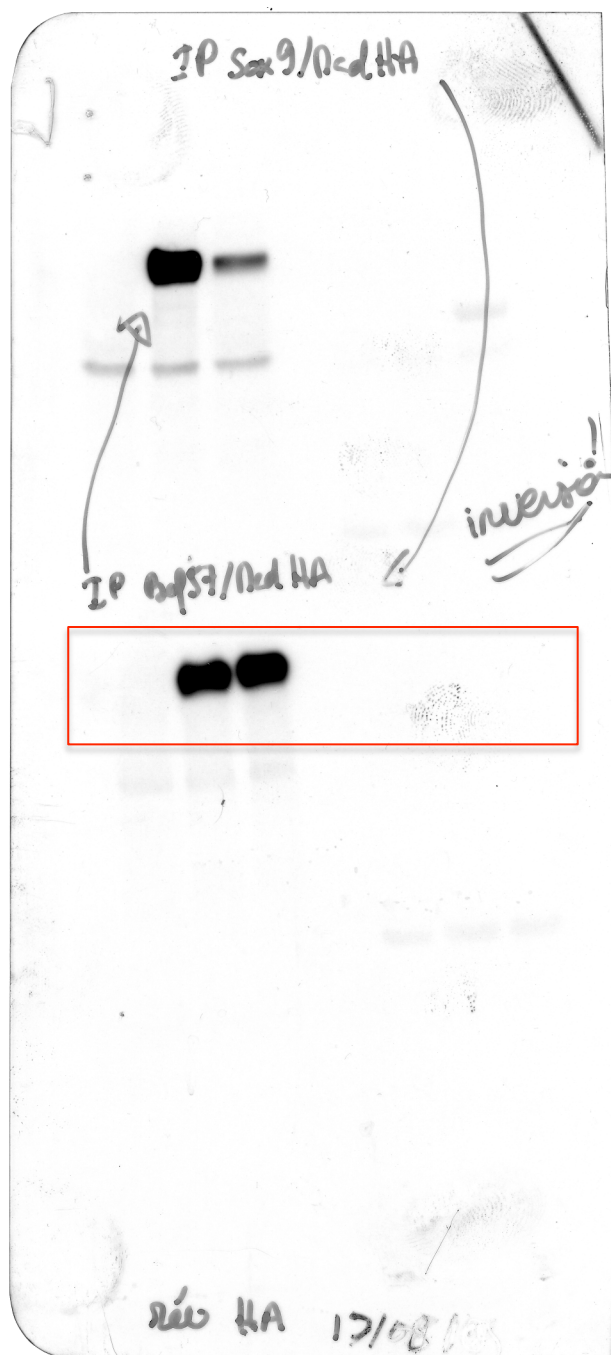

ANTI - HA

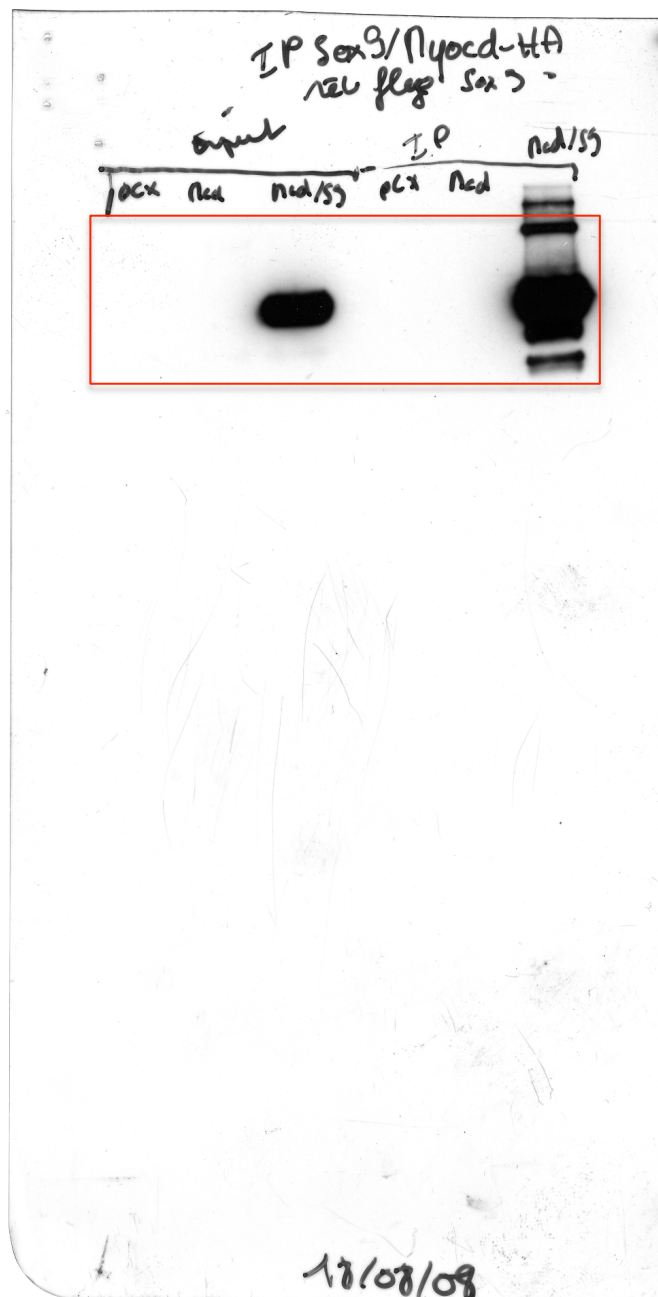

ANTI-FLAG

Supplement: S4 File — (PDF) [file pone.0211924.s004.pdf]

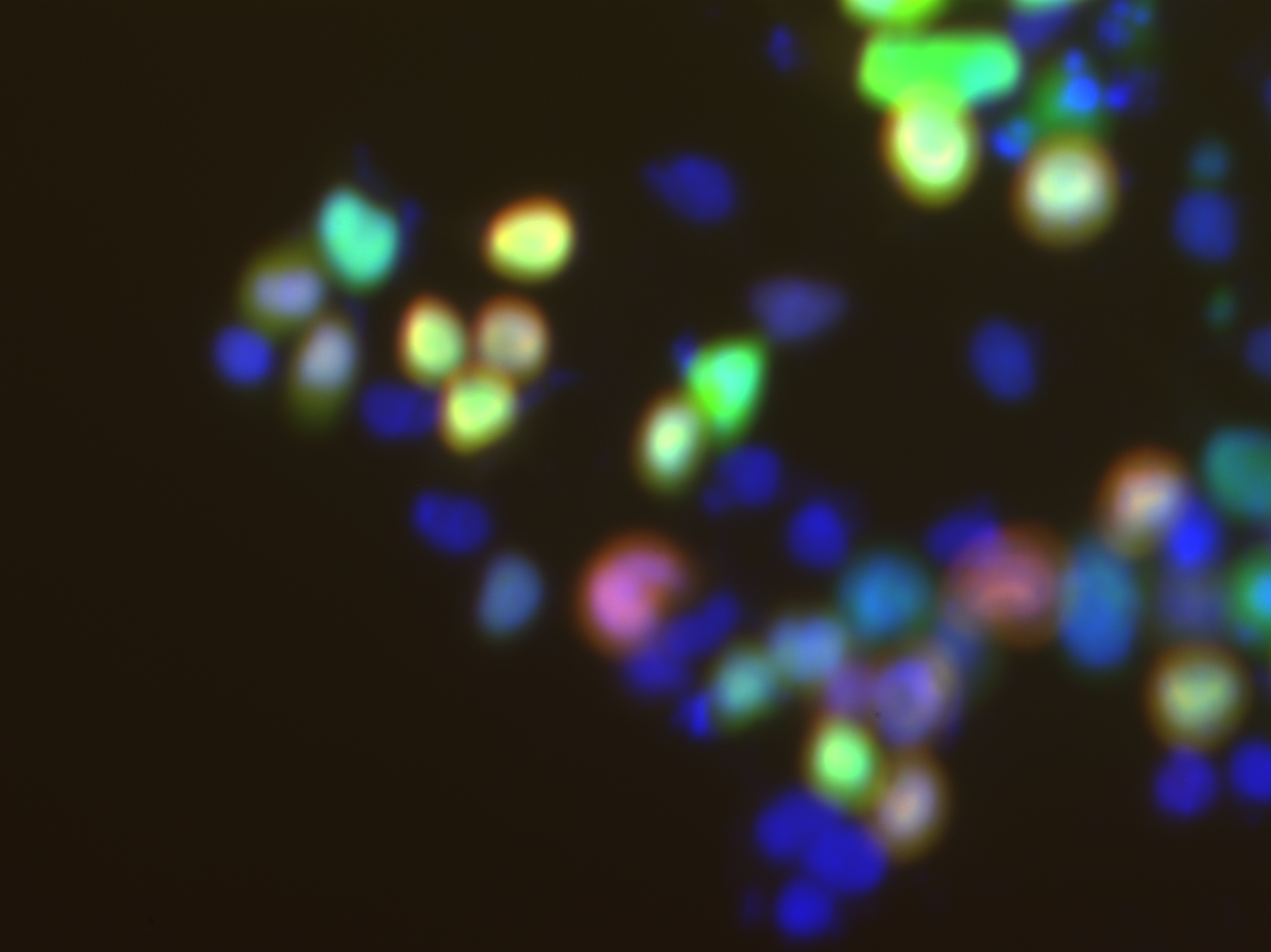

Supplement: S5 File — (ZIP) [file pone.0211924.s005.zip › Fig 1E top pannel/S9 T3fl 1_z01(DAPI+FITC+Cy3 5).TIF]

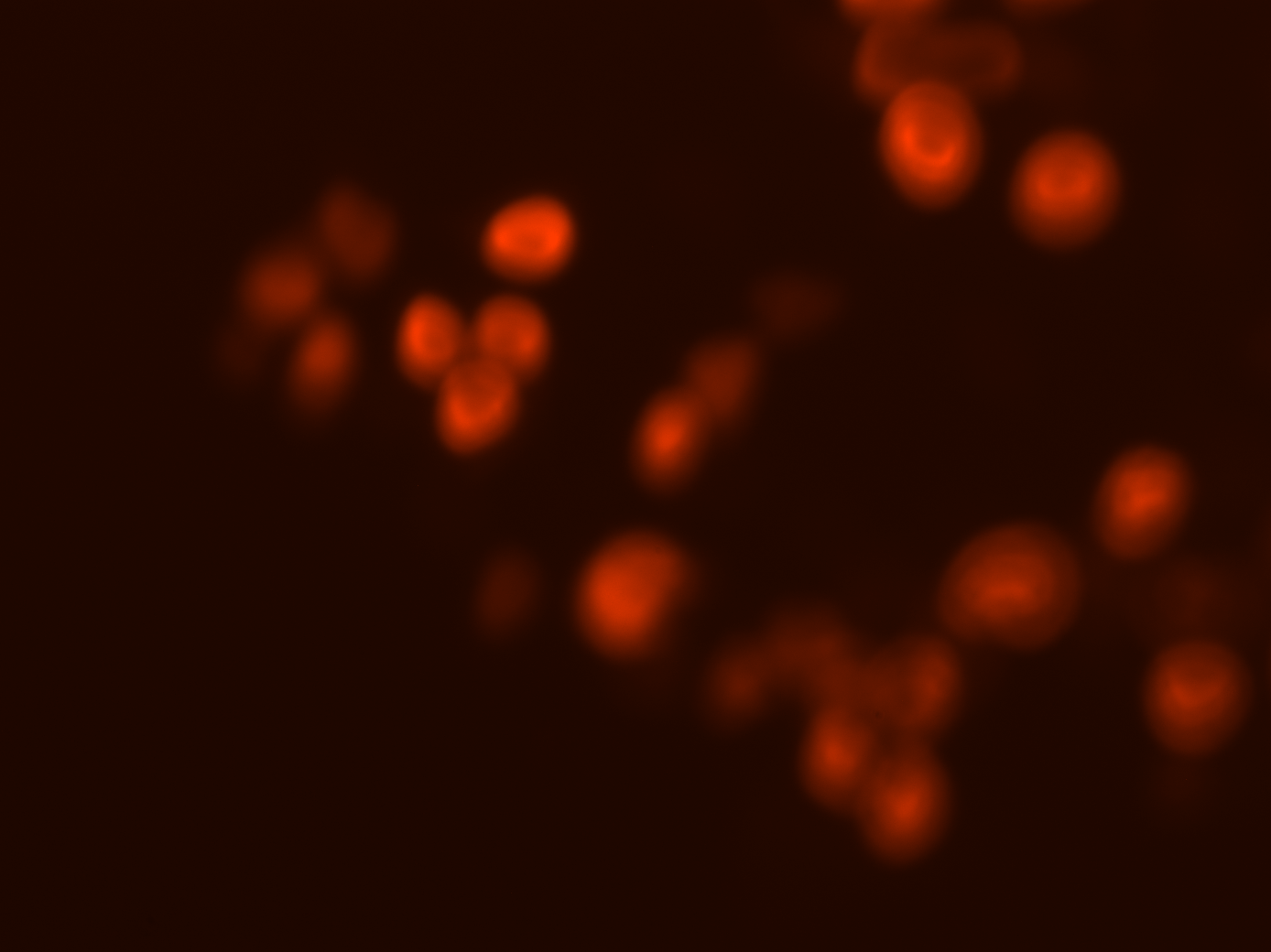

Supplement: S5 File — (ZIP) [file pone.0211924.s005.zip › Fig 1E top pannel/S9 T3fl 1_z01Cy3 5.TIF]

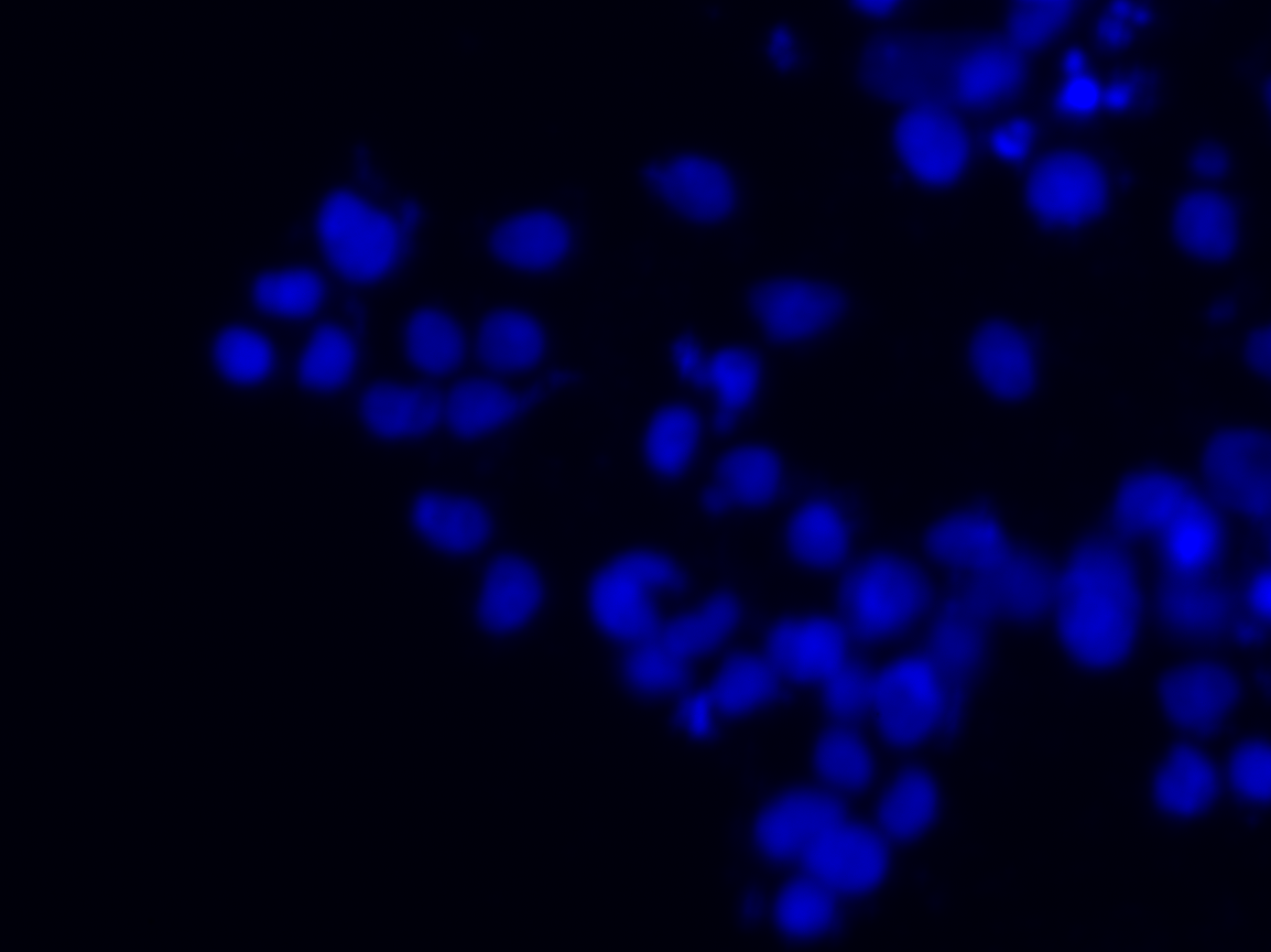

Supplement: S5 File — (ZIP) [file pone.0211924.s005.zip › Fig 1E top pannel/S9 T3fl 1_z01DAPI.TIF]

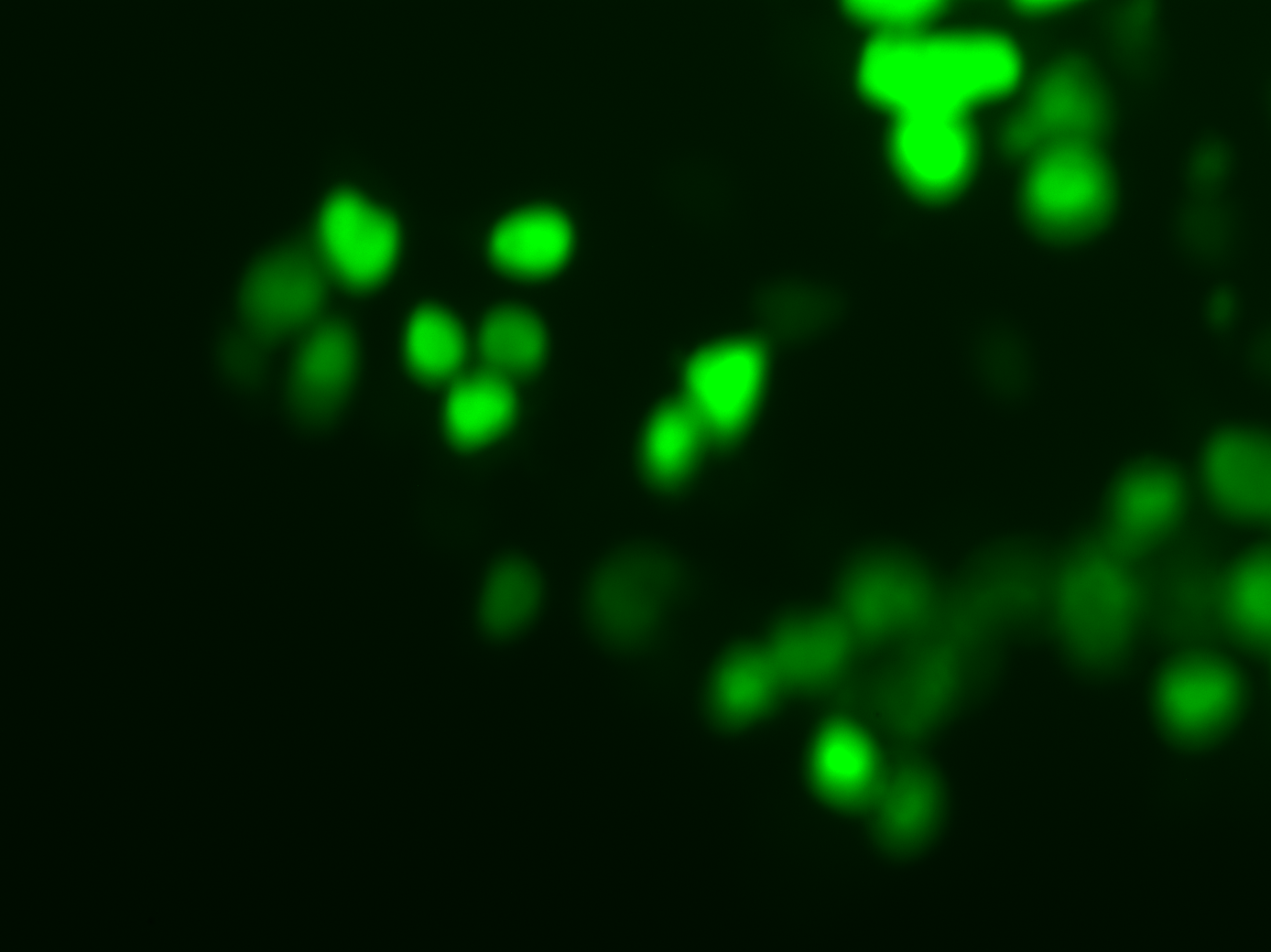

Supplement: S5 File — (ZIP) [file pone.0211924.s005.zip › Fig 1E top pannel/S9 T3fl 1_z01FITC.TIF]

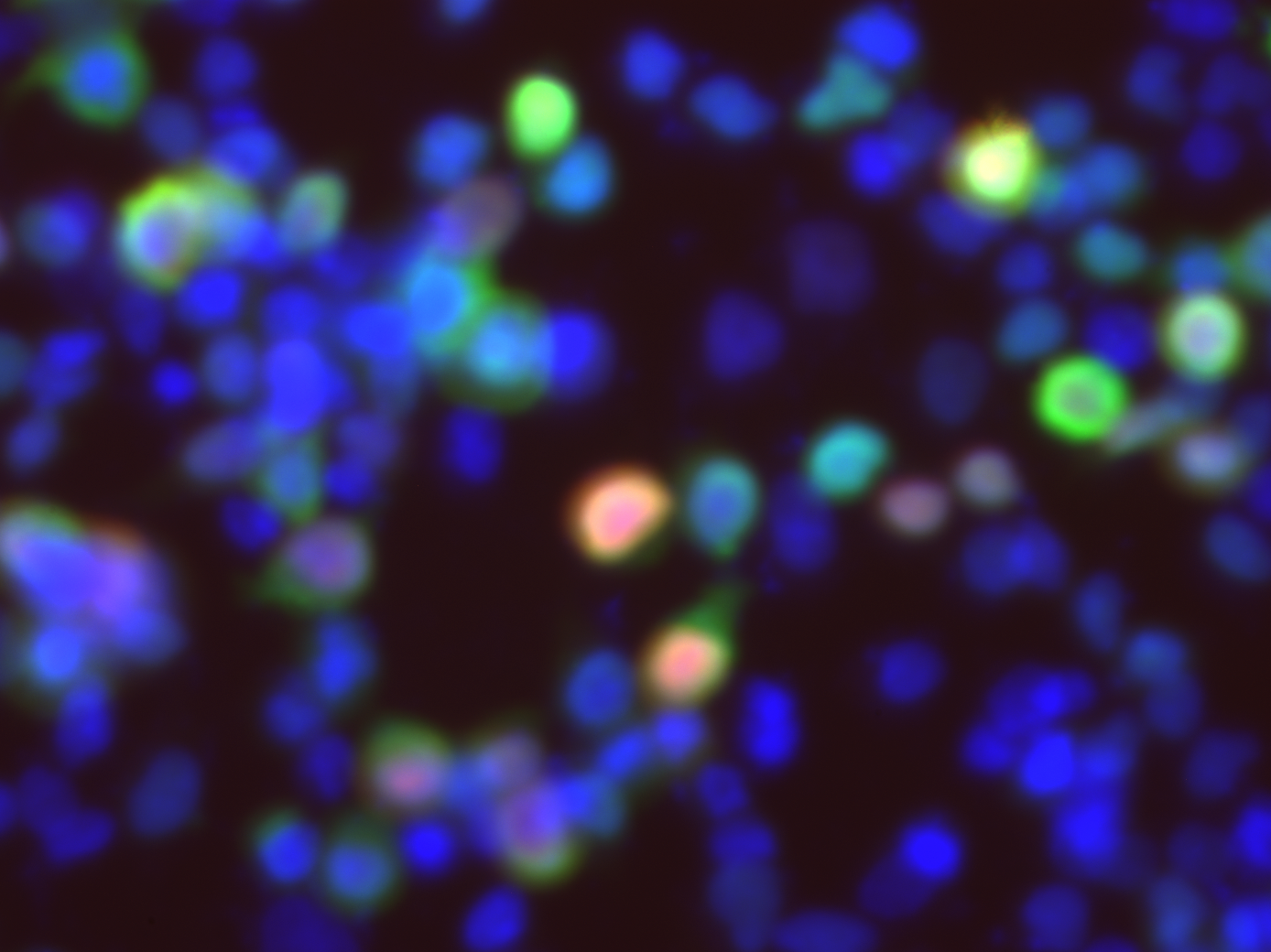

Supplement: S6 File — (ZIP) [file pone.0211924.s006.zip › Fig 1E lower pannel/S9 T3N 1_z01(DAPI+FITC+Cy3 5).TIF]

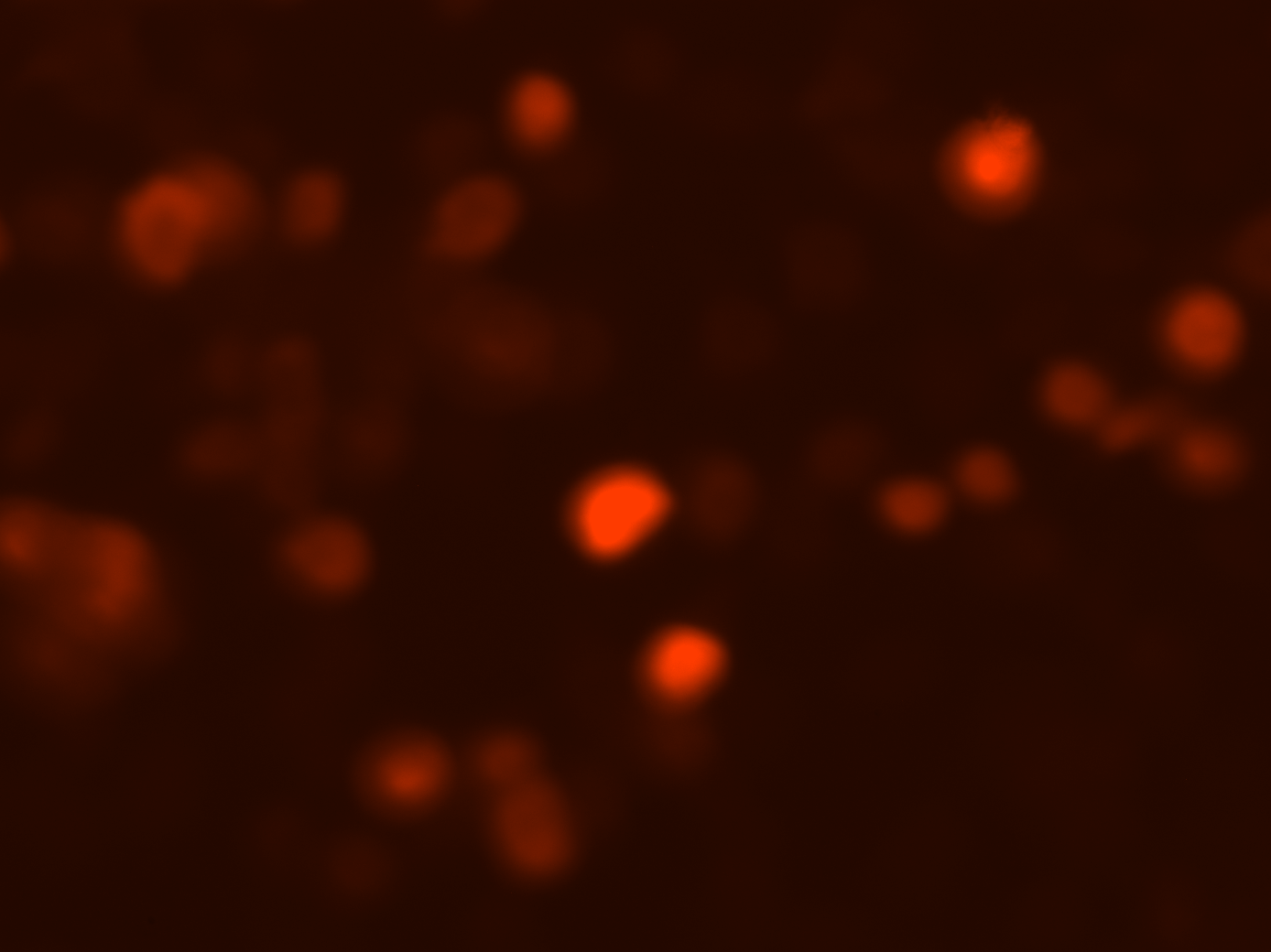

Supplement: S6 File — (ZIP) [file pone.0211924.s006.zip › Fig 1E lower pannel/S9 T3N 1_z01Cy3 5.TIF]

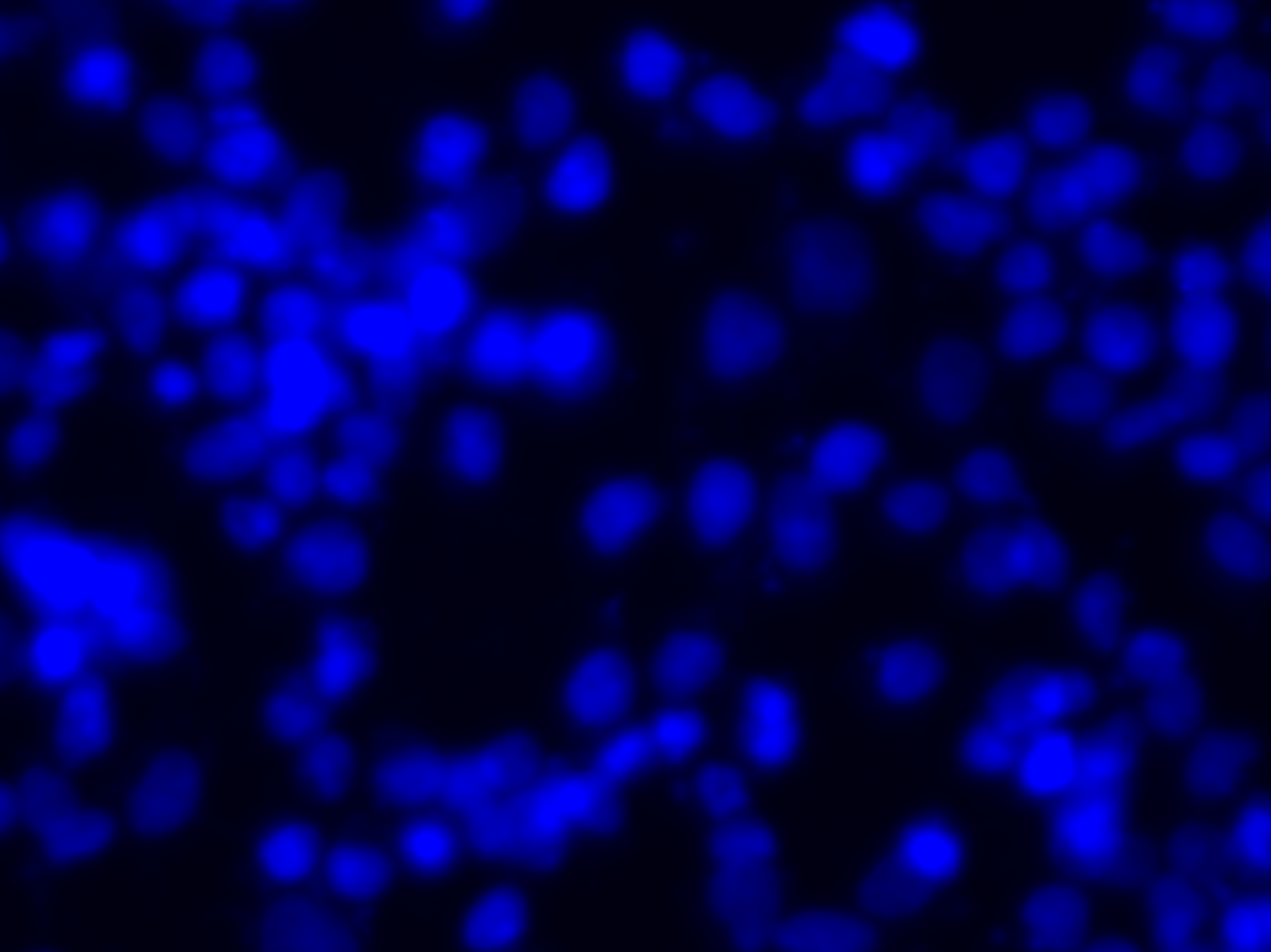

Supplement: S6 File — (ZIP) [file pone.0211924.s006.zip › Fig 1E lower pannel/S9 T3N 1_z01DAPI.TIF]

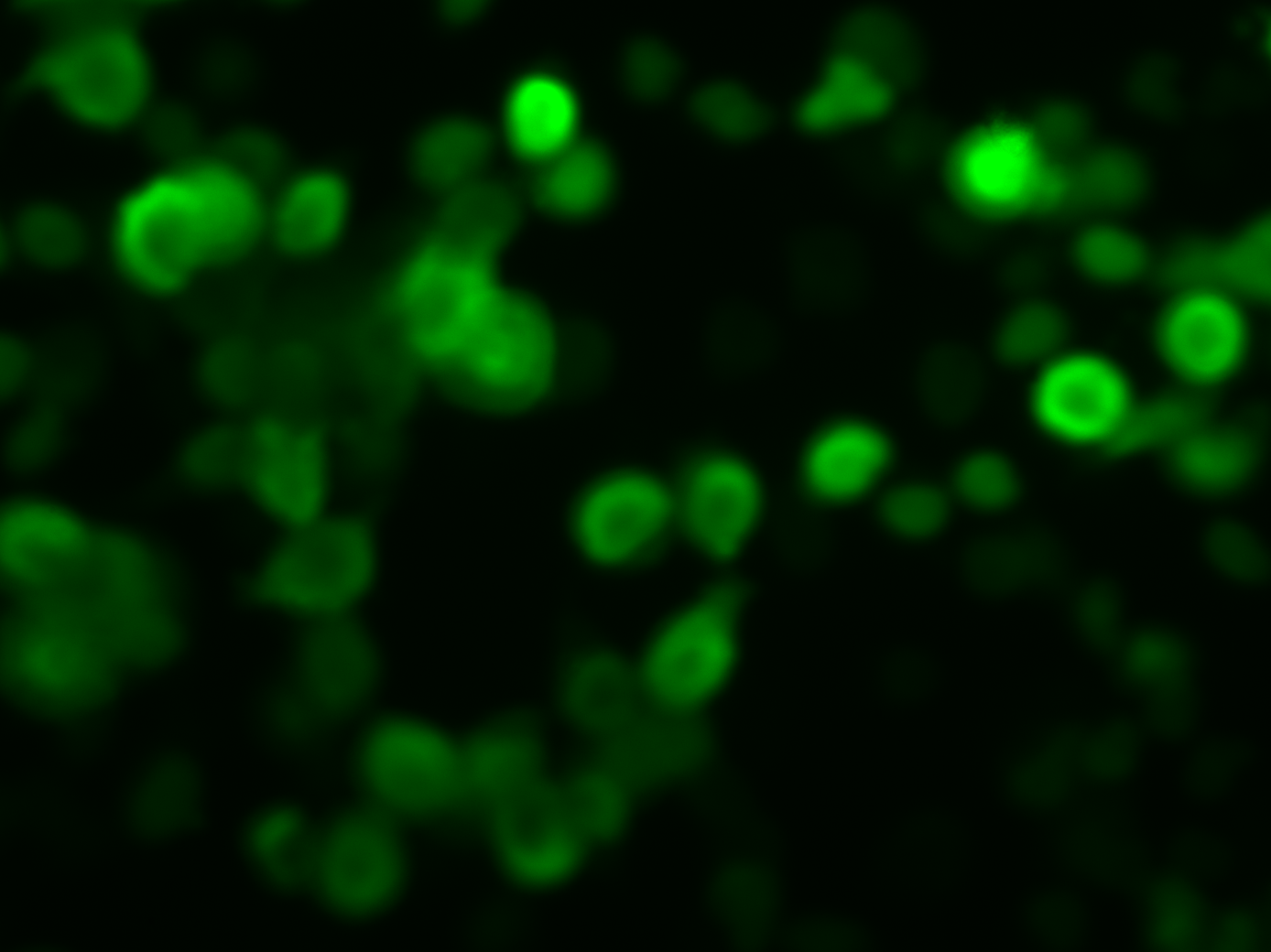

Supplement: S6 File — (ZIP) [file pone.0211924.s006.zip › Fig 1E lower pannel/S9 T3N 1_z01FITC.TIF]

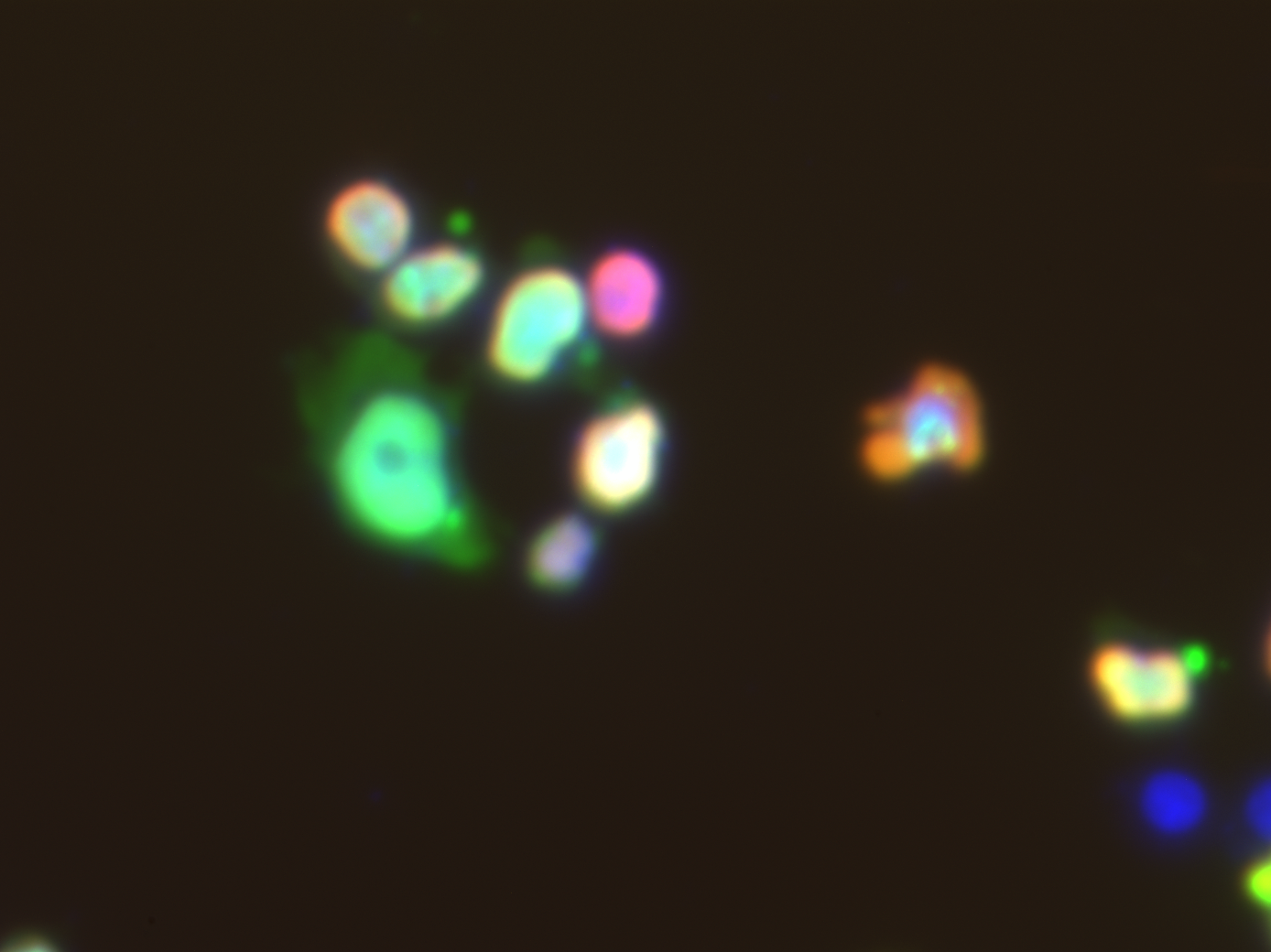

Supplement: S19 File — (ZIP) [file pone.0211924.s019.zip › Fig S4C Top pannel/mcd T3fl 1_z01(DAPI+FITC+Cy3 5).TIF]

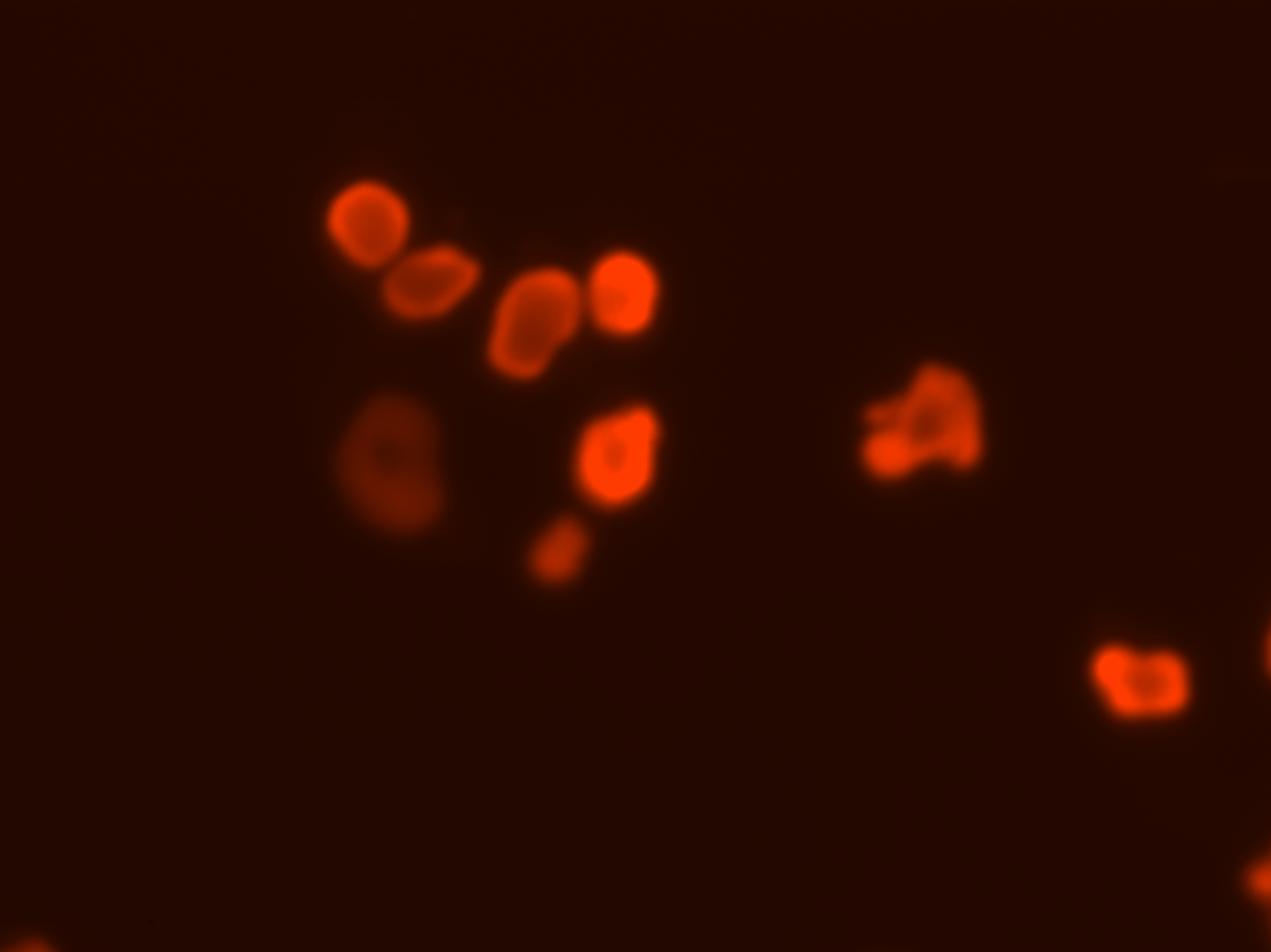

Supplement: S19 File — (ZIP) [file pone.0211924.s019.zip › Fig S4C Top pannel/mcd T3fl 1_z01Cy3 5.TIF]

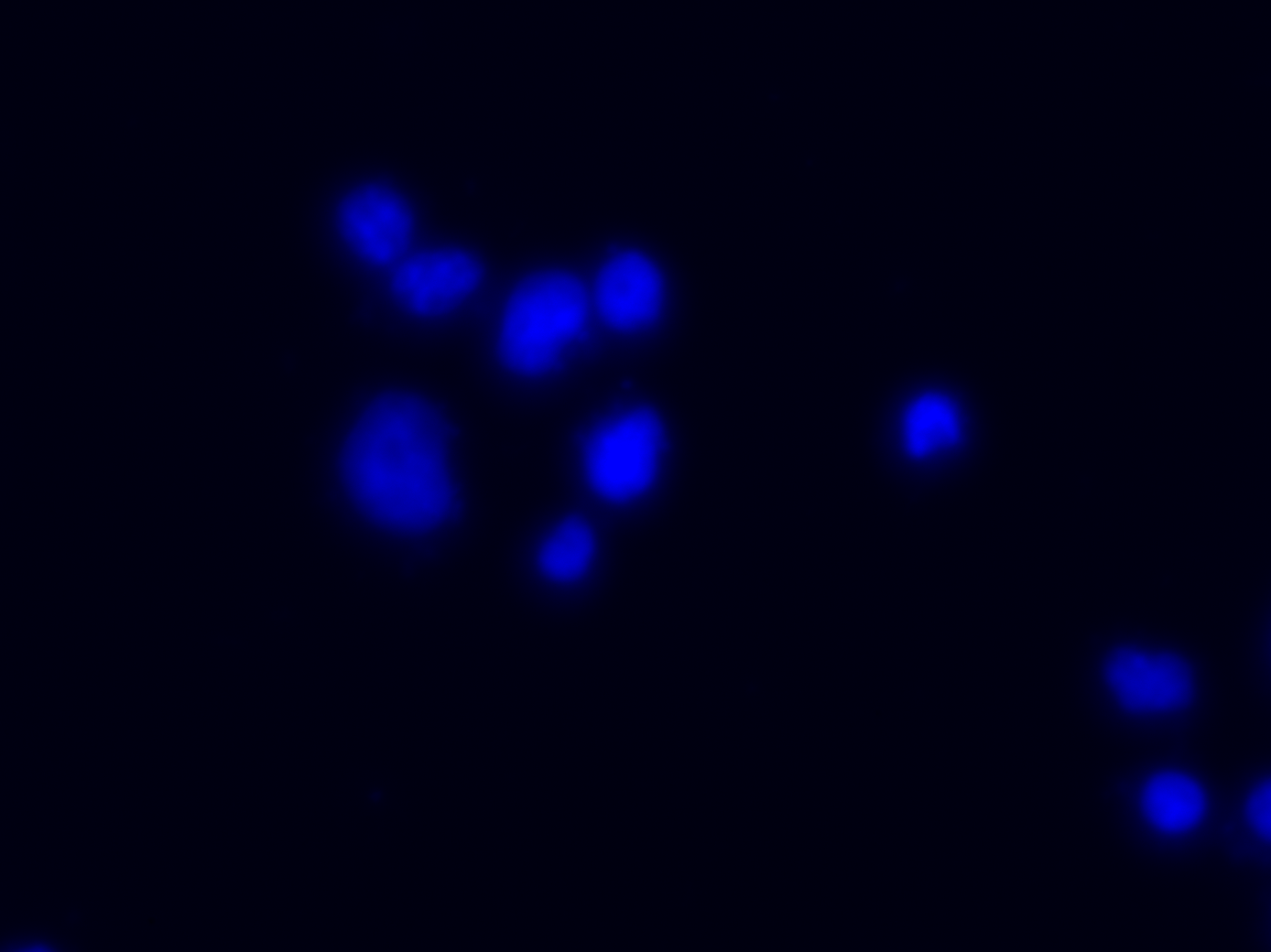

Supplement: S19 File — (ZIP) [file pone.0211924.s019.zip › Fig S4C Top pannel/mcd T3fl 1_z01DAPI.TIF]

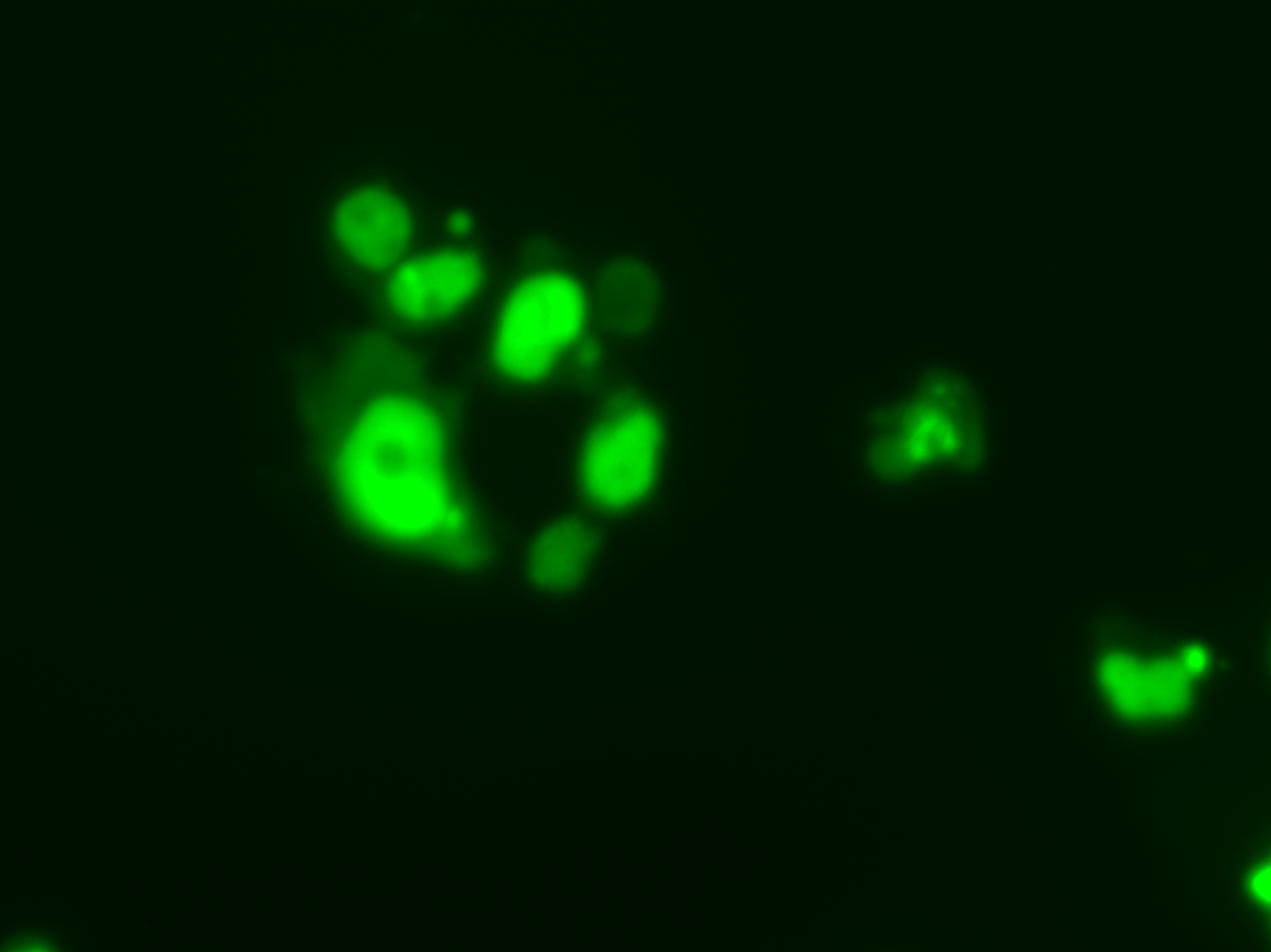

Supplement: S19 File — (ZIP) [file pone.0211924.s019.zip › Fig S4C Top pannel/mcd T3fl 1_z01FITC.TIF]

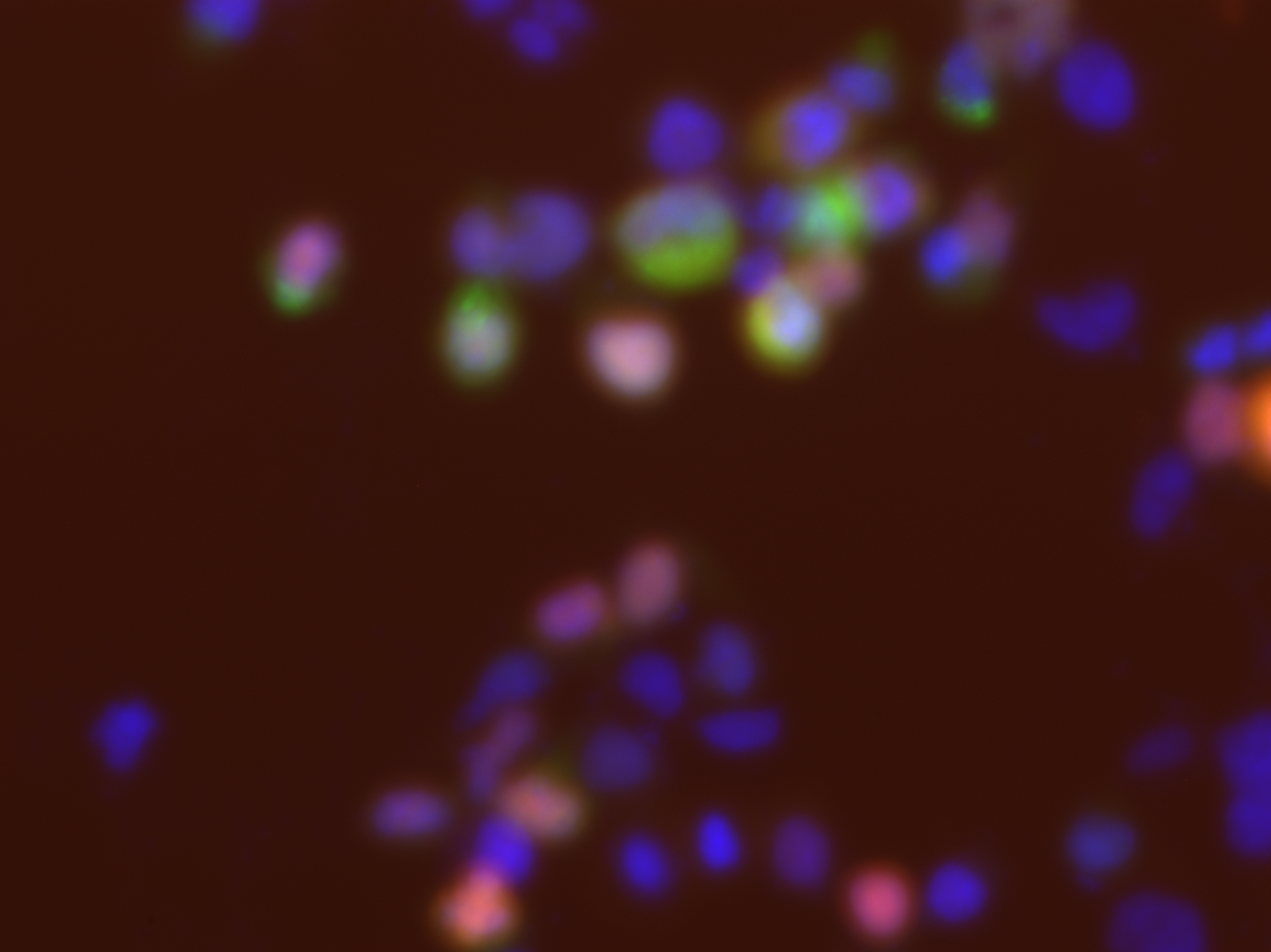

Supplement: S20 File — (ZIP) [file pone.0211924.s020.zip › Fig S4C Lower pannel/Mcd T3N 1_z01(DAPI+FITC+Cy3 5).TIF]

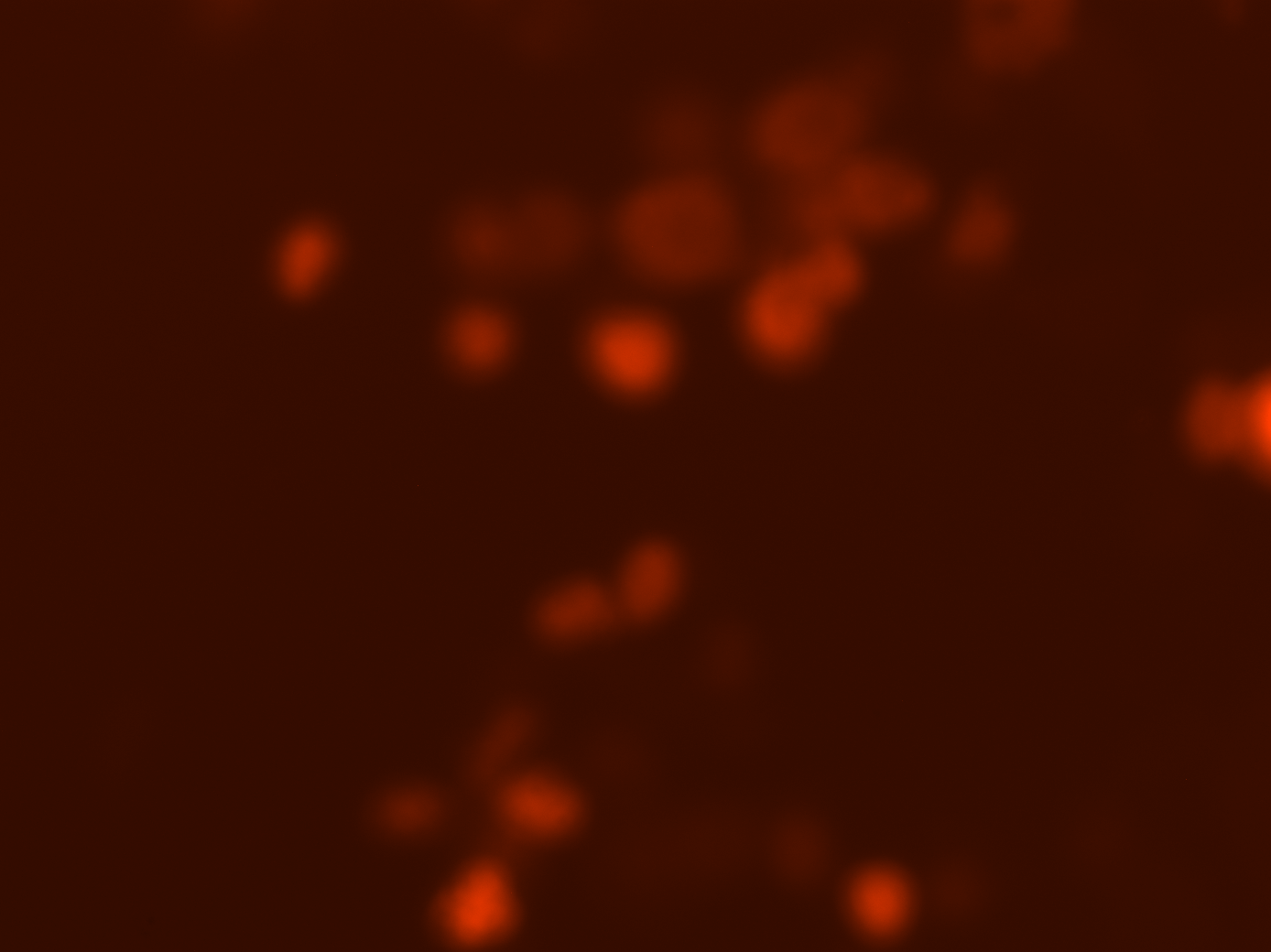

Supplement: S20 File — (ZIP) [file pone.0211924.s020.zip › Fig S4C Lower pannel/Mcd T3N 1_z01Cy3 5.TIF]

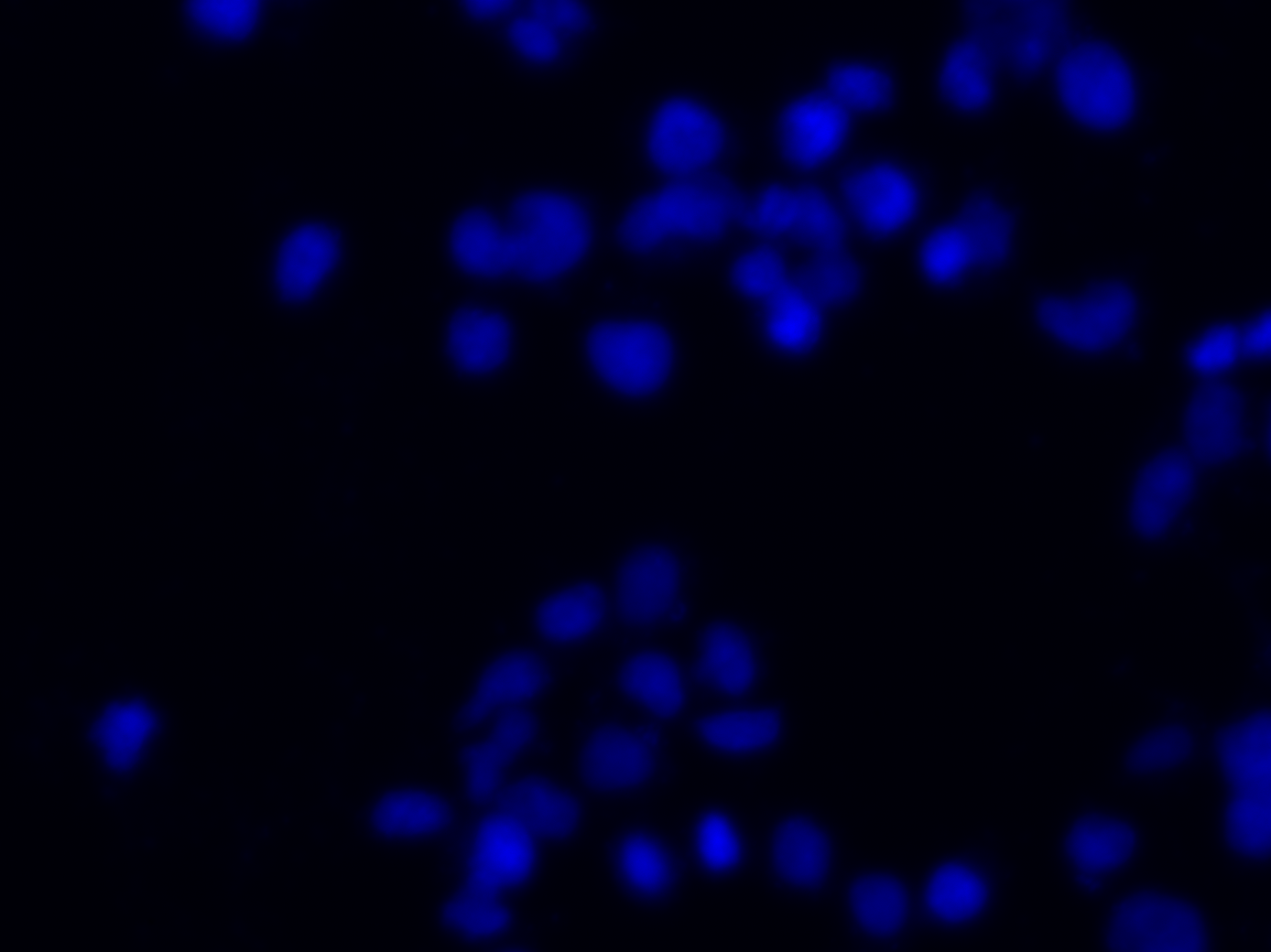

Supplement: S20 File — (ZIP) [file pone.0211924.s020.zip › Fig S4C Lower pannel/Mcd T3N 1_z01DAPI.TIF]

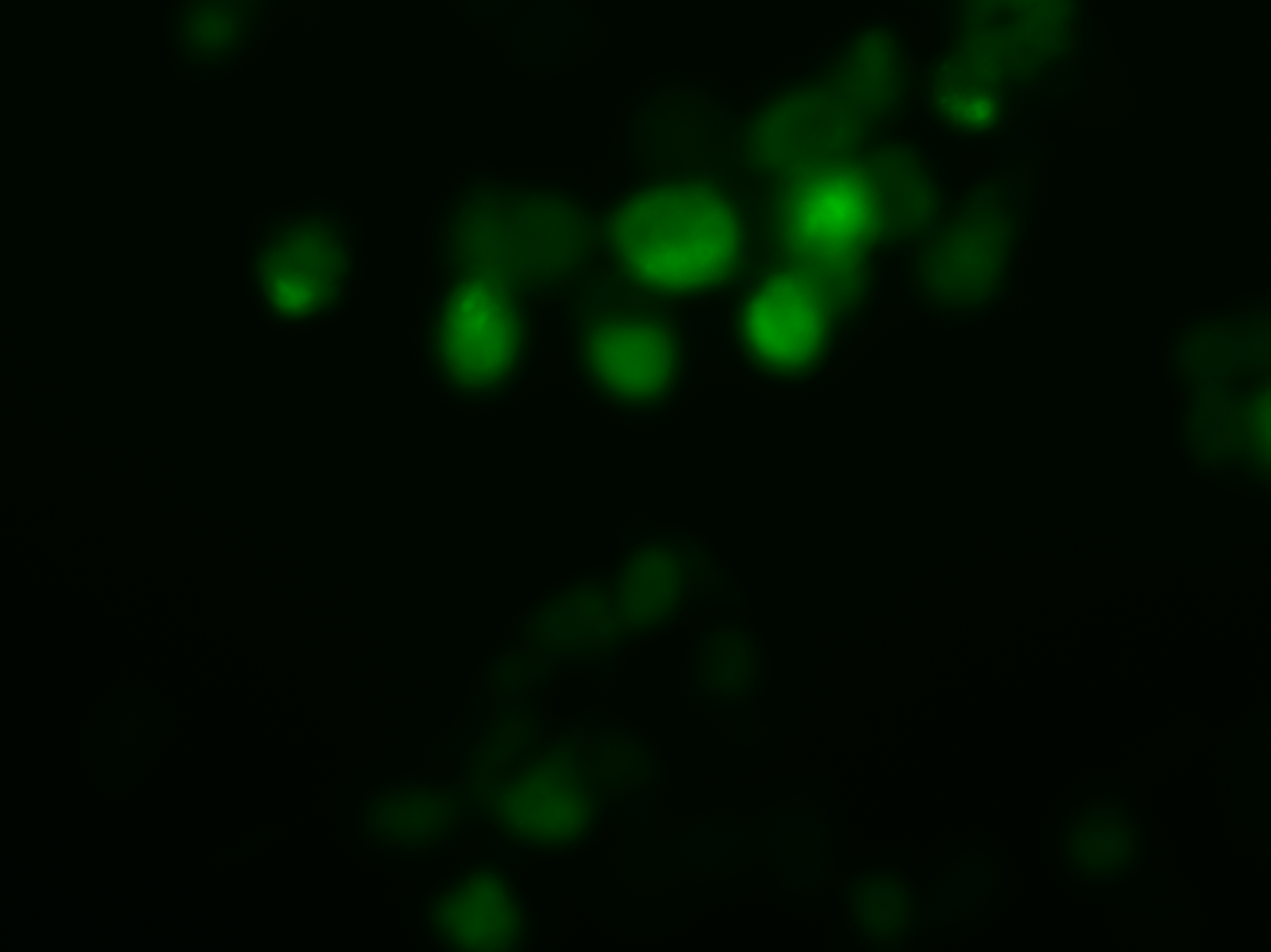

Supplement: S20 File — (ZIP) [file pone.0211924.s020.zip › Fig S4C Lower pannel/Mcd T3N 1_z01FITC.TIF]

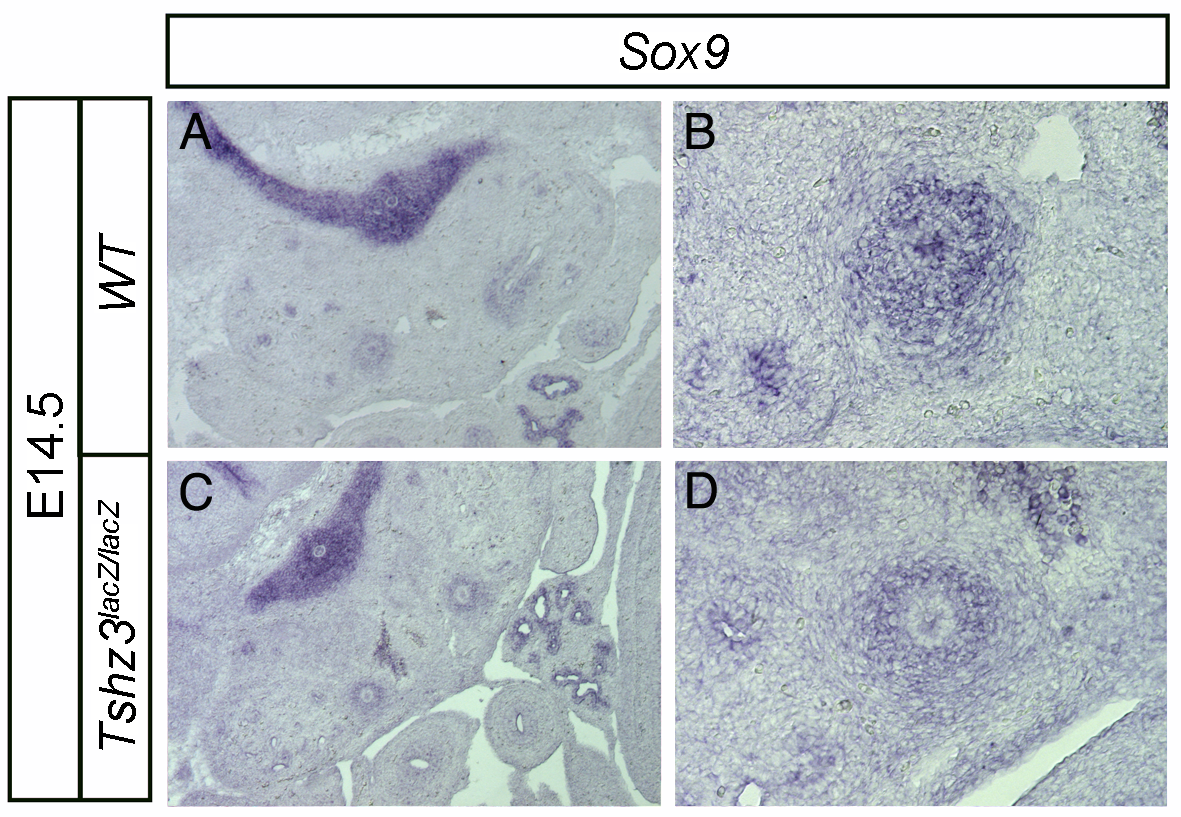

Supplement: S21 File — Expression of Sox9 is not affected in Tshz3 mutant ureters. (A, B) Sox9 expression in wild type ureter. (C, D) Sox9 expression in Tshz3 mutant ureter. (TIF) [file pone.0211924.s021.tif]

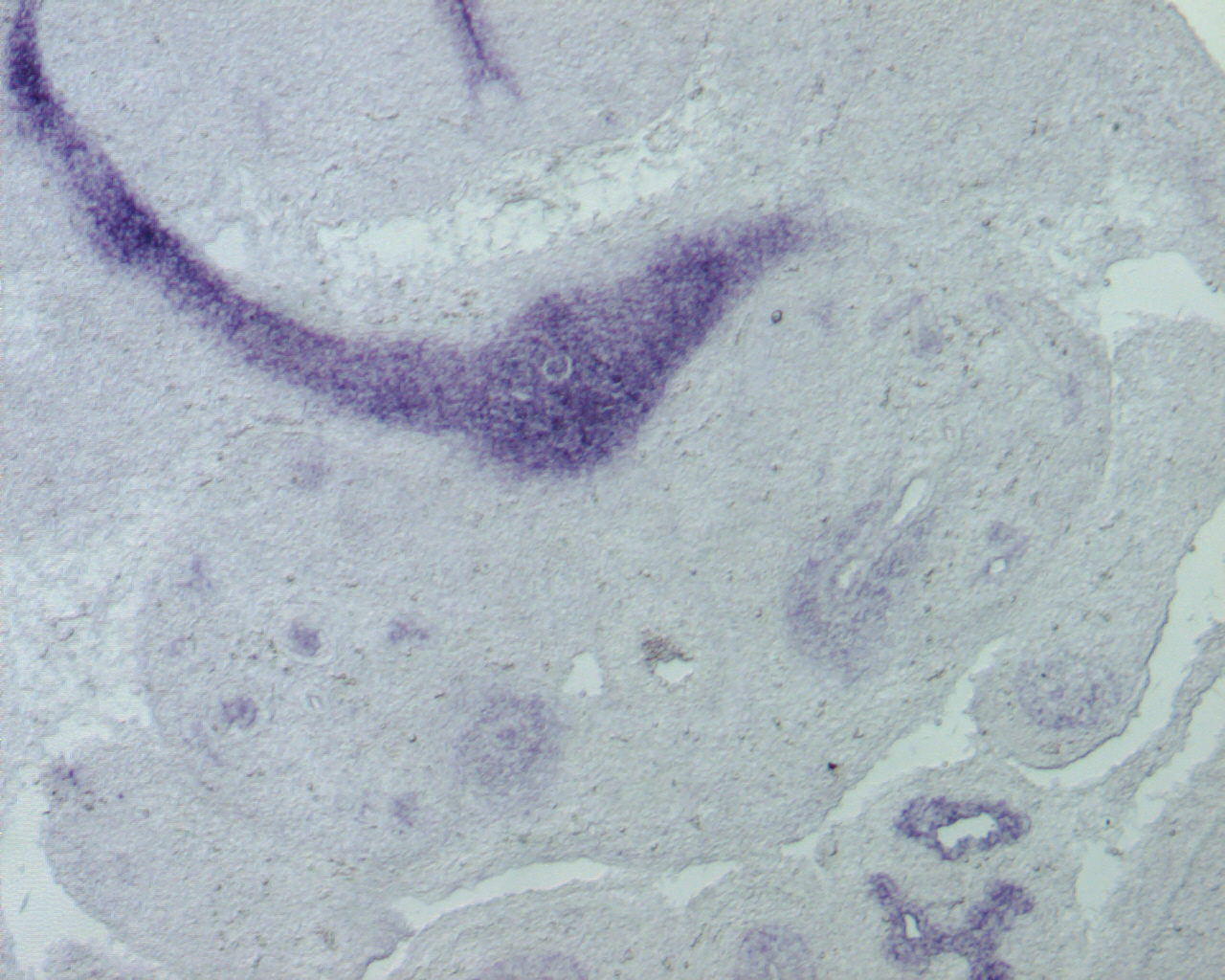

Supplement: S22 File — (TIF) [file pone.0211924.s022.tif]

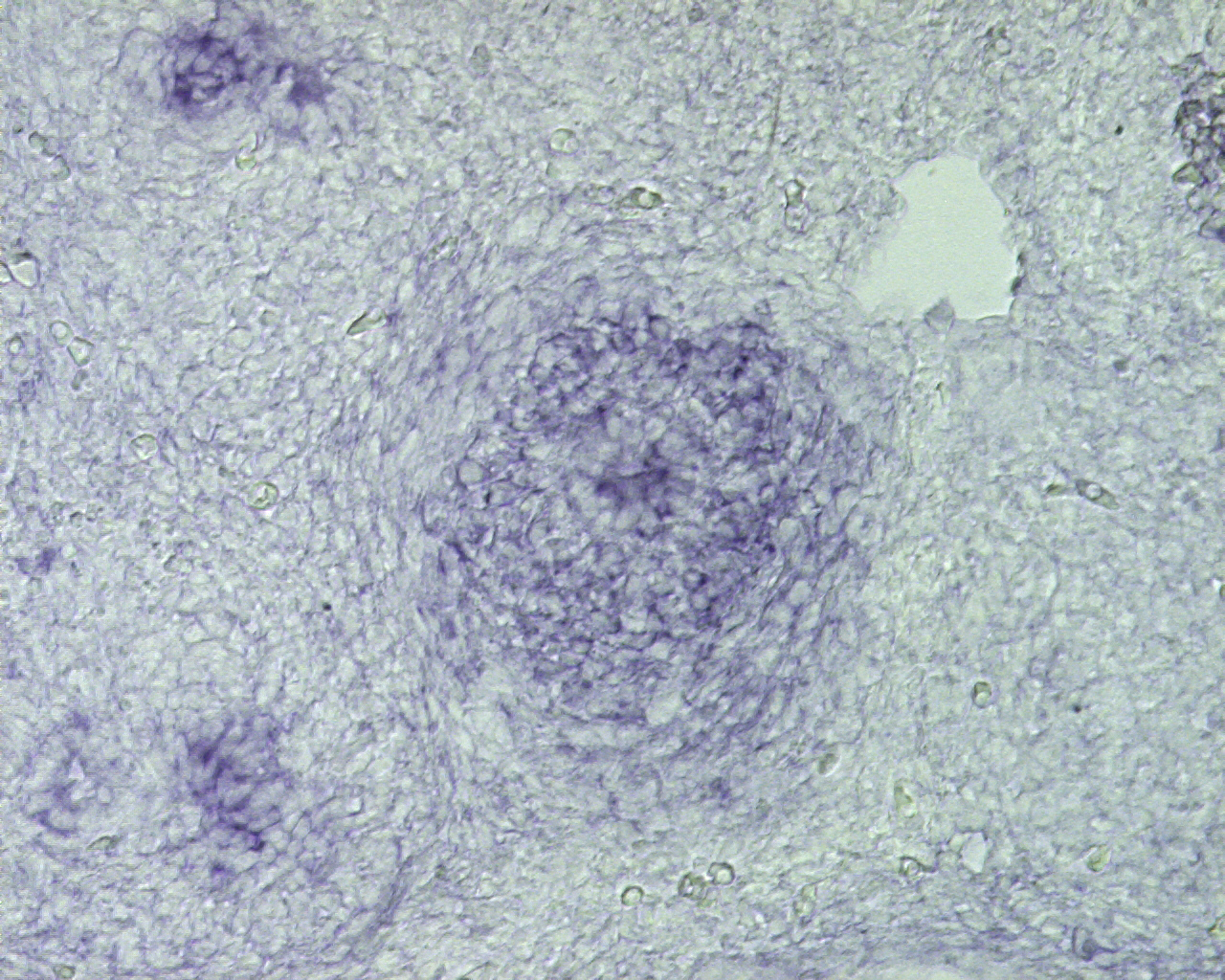

Supplement: S23 File — (TIF) [file pone.0211924.s023.tif]

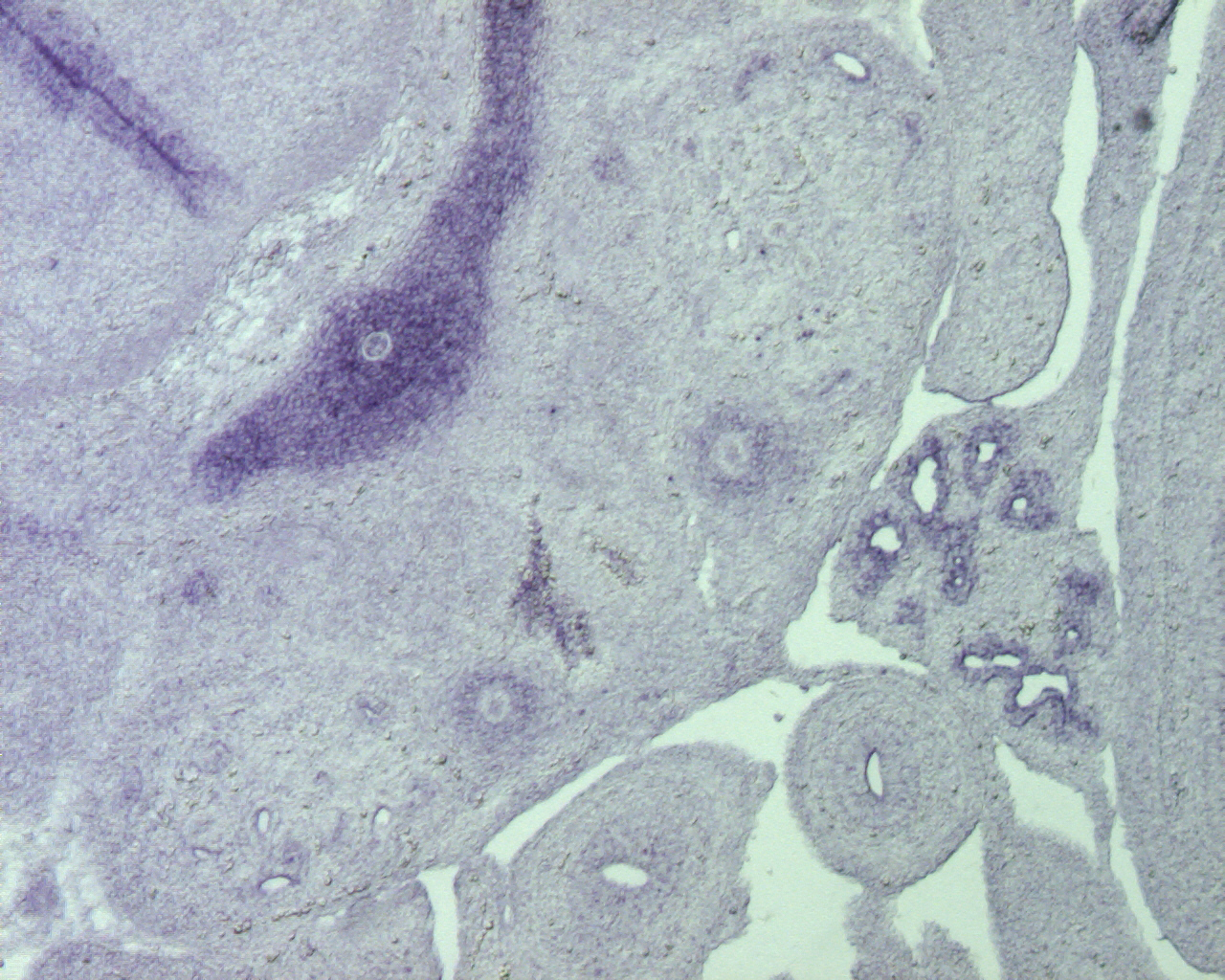

Supplement: S24 File — (TIF) [file pone.0211924.s024.tif]

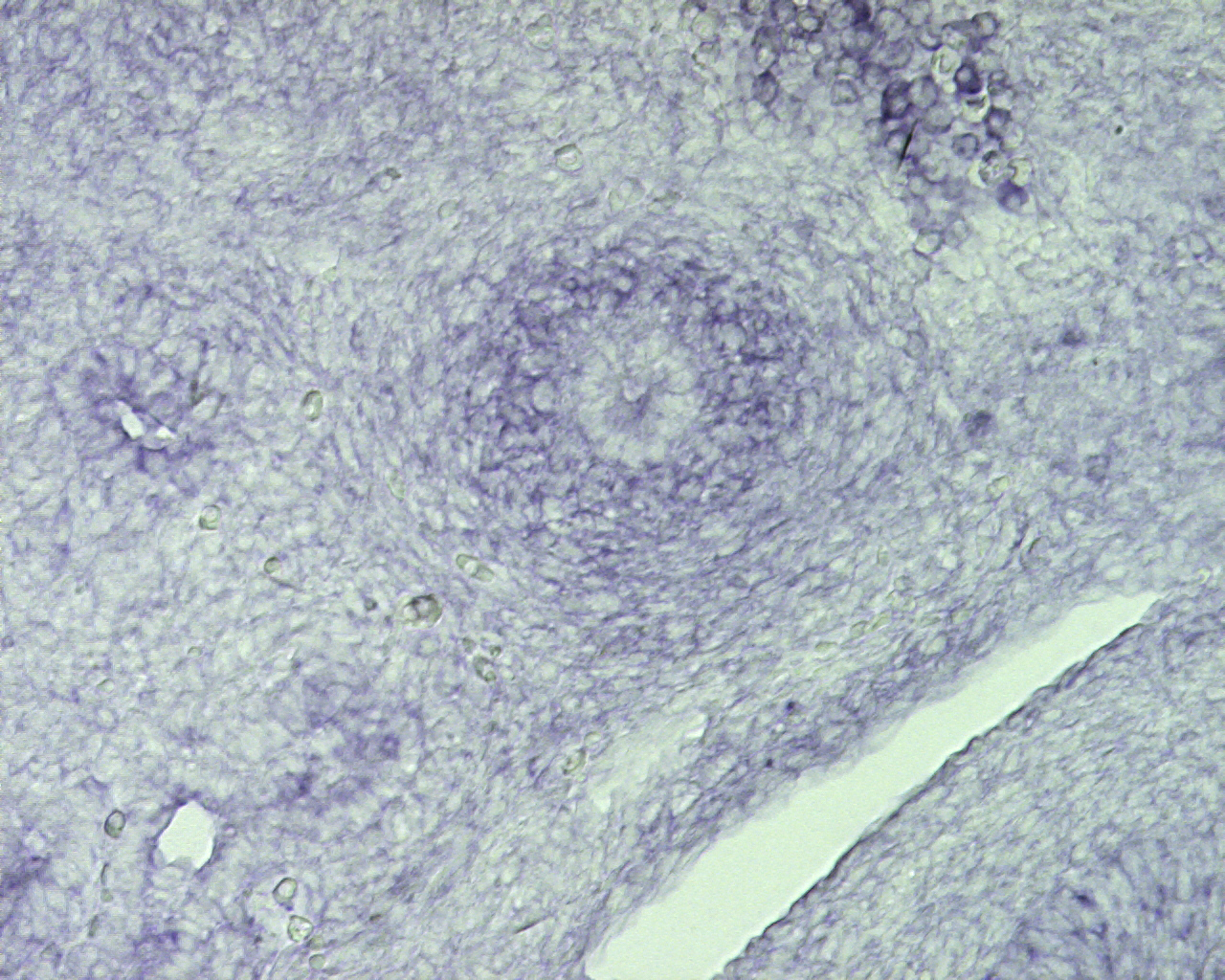

Supplement: S25 File — (TIF) [file pone.0211924.s025.tif]
